# The Complex History of Genome Duplication and Hybridization in North American Gray Treefrogs

**DOI:** 10.1093/molbev/msab316

**Published:** 2021-11-13

**Authors:** William W Booker, H Carl Gerhardt, Alan R Lemmon, Margaret B Ptacek, Alyssa T B Hassinger, Johannes Schul, Emily Moriarty Lemmon

**Affiliations:** 1 Department of Biological Science, Florida State University, Tallahassee, FL, USA; 2 Division of Biological Sciences, University of Missouri, Columbia, MO, USA; 3 Department of Scientific Computing, Florida State University, Tallahassee, FL, USA; 4 Department of Biological Sciences, Clemson University, Clemson, SC, USA; 5 Department of Evolution, Ecology, and Organismal Biology, Ohio State University, Columbus, OH, USA

**Keywords:** hybridization, speciation, polyploid, amphibian, Bayesian model testing, population genetics

## Abstract

Polyploid speciation has played an important role in evolutionary history across the tree of life, yet there remain large gaps in our understanding of how polyploid species form and persist. Although systematic studies have been conducted in numerous polyploid complexes, recent advances in sequencing technology have demonstrated that conclusions from data-limited studies may be spurious and misleading. The North American gray treefrog complex, consisting of the diploid *Hyla chrysoscelis* and the tetraploid *H. versicolor*, has long been used as a model system in a variety of biological fields, yet all taxonomic studies to date were conducted with only a few loci from nuclear and mitochondrial genomes. Here, we utilized anchored hybrid enrichment and high-throughput sequencing to capture hundreds of loci along with whole mitochondrial genomes to investigate the evolutionary history of this complex. We used several phylogenetic and population genetic methods, including coalescent simulations and testing of polyploid speciation models with approximate Bayesian computation, to determine that *H. versicolor* was most likely formed via autopolyploidization from a now extinct lineage of *H. chrysoscelis*. We also uncovered evidence of significant hybridization between diploids and tetraploids where they co-occur, and show that historical hybridization between these groups led to the re-formation of distinct polyploid lineages following the initial whole-genome duplication event. Our study indicates that a wide variety of methods and explicit model testing of polyploid histories can greatly facilitate efforts to uncover the evolutionary history of polyploid complexes.

## Introduction

Hybridization is universal. As the genomic and taxonomic breadth of systematic studies continues to increase, the tree of life less resembles a series of simple bifurcations and instead becomes better defined by a complex network of interactions, exchanges, and rearrangements. Understanding how to disentangle these networks, and how they translate into the formation of distinct and identifiable species, however, remains elusive.

Polyploidization represents a unique form of speciation defined by an increase in the number of chromosome sets a species has in comparison to its ancestral taxa. Though unique in mechanism, polyploidy has undoubtedly played a major role in shaping the tree of life. While only common in some animal clades (Mable et al. 2011), polyploidy is well known as a major driver of diversification in plants (Blischak et al. 2018; One Thousand Plant Transcriptomes Initiative 2019), has occurred at least twice in early vertebrate history (Gregory and Mable 2005; Mable et al. 2011), is likely common in prokaryotes and fungi (Albertin and Marullo 2012), and has recently been discovered to play a major role in the diversification of insects (Li et al. 2018).

Whereas allopolyploidy is defined by hybridization, both allopolyploids and autopolyploids frequently hybridize with relatives of different ploidies during and after their formation (Bogart and Bi 2013; Soltis et al. 2014). Although theory and empirical work demonstrate substantial fitness consequences to interploid hybridization (Ramsey and Schemske 1998), there are nonetheless scenarios where hybridization across ploidies may be adaptive—such as increasing genetic diversity and providing the genetic material for novel adaptations or the introgression of locally adapted alleles (Soltis and Rieseberg 1986; Rieseberg et al. 1996; Petit et al. 1999). The processes that may facilitate adaptive introgression from interploid hybridization are, however, poorly understood. In some cases, triploid offspring can produce functional gametes (Krahulcová and Krahulec 2000) that can result in tetraploid offspring (Peckert and Chrtek 2006), but in others there may be complete reproductive isolation between diploids and tetraploids (i.e., triploid-block, Marks 1966). In instances where there is a triploid-block but higher level ploidies in the complex exist, gene flow across ploidies can occur between other intermediates (e.g., pentaploids, Peskoller et al. 2021; Sutherland and Galloway 2021). Despite strong evidence of gene flow across ploidies in some animals (Bogart et al. 2020; Novikova et al. 2020), these processes are even further understudied. In general, many aspects of polyploidy may be poorly understood because the majority of our polyploid knowledge comes from research in just a few model systems (Soltis et al. 2016). To fill these gaps and to achieve a more general understanding of polyploidy and the consequences of interploid hybridization, more polyploid systems whose origins and evolutionary history are well-defined must be developed.

The North American gray treefrog complex, comprised the diploid *Hyla chrysoscelis* and tetraploid *H. versicolor* (Le Conte 1825; Johnson 1963; Wasserman 1970; Bogart and Wasserman 1972), is one polyploid system that has been used to study biological phenomena in a diverse fields (e.g., behavioral ecology, evolution, genetics, and neurobiology; Littlejohn et al. 1960; Blair 1962; Gerhardt 1974, 1978; Ralin and Selander 1979; Storey and Storey 1985; Wells and Taigen 1986; Gerhardt and Doherty 1988; Ptacek et al. 1994; Relyea and Mills 2001; Gerhardt and Huber 2002; Schul and Bush 2002; Endepols et al. 2004; Holloway et al. 2006), but whose origins and evolutionary history remain largely in question (Holloway et al. 2006; Bogart and Bi 2013; Bogart et al. 2020). This is not for lack of effort. Considerable work has been conducted in the attempt to disentangle this unique complex’s history since the discovery of its multiple “call-races” in 1936 (Noble and Hassler 1936). Though we now know these “call-races” are a direct result of the physiological changes related to polyploidization, namely an increase in cell size (Ueda 1993; Keller and Gerhardt 2001; Tucker and Gerhardt 2012), the largely identical morphology and ecology of *H. chrysoscelis* and *H. versicolor* along with the mosaic distribution of the two species (i.e., call races) has posed a particularly challenging problem for researchers trying to define their relationships.

The discovery of the complex’s diploid–tetraploid nature by Wasserman (1970) and Bogart and Wasserman (1972) provided substantial clarity to the complex and a sufficient explanation for its call variation, but the origins of tetraploidy in *H. versicolor*, alongside the systematic relationships of gray treefrogs and their relatives, remained unknown. In their original description, Bogart and Wasserman (1972) first proposed an effectively autopolyploid origin of *H. versicolor* through an intermediary triploid bridge. Since this hypothesis was proposed, however, numerous competing hypotheses proposing various scenarios of auto- or allopolyploidy, the number of origins, origin timing, hybridization, and the populations and species involved have been suggested. To date, the two most recent studies addressing the origin of polyploidy in gray treefrogs come to largely different and incompatible conclusions including whether *H. versicolor* formed via allo- (Holloway et al. 2006) or autopolyploidy (Bogart et al. 2020).

Although our methodology for inferring the relationships between polyploid taxa and their relatives has drastically improved since Bogart and Wasserman (1972; Blischak et al. 2018), much of the disparity between the conclusions of subsequent studies are not necessarily the result of conflicting data, but rather because inferences made from pattern alone can be misleading for systems with complex evolutionary histories. For example, recovering evidence of novel alleles in a polyploid could be explained equally well by either allopolyploidy, whereby novel alleles descended from an extinct heterospecific taxon, or by autopolyploidy, where novel alleles originated from an extinct conspecific population. Gene conversion, interploid hybridization, and mixed chromosomal inheritance introduce further confoundments (Innan and Kondrashov 2010; Dufresne et al. 2014). As such, the phylogenies of Ptacek et al. (1994) and Holloway et al. (2006), and the unique *H. versicolor* alleles from Ralin and Selander (1979), are not conclusive evidence of multiple allopolyploid origins from multiple extant and extinct species as proposed by Holloway et al. (2006). Indeed, as Bogart and Bi (2013) suggest, the data of the Holloway et al. (2006) study are not explicitly incompatible with alternative conclusions such as an autopolyploid origin of *H. versicolor*, and Bogart et al. (2020) showed that hybridization across ploidies of the two species is likely—a condition Holloway et al. (2006) did not consider.

Here, we attempt to resolve the conflicting conclusions from previous research by taking a comprehensive look at the origins of the polyploid North American gray treefrog complex and their evolutionary history since formation. Using hundreds of loci and whole mitochondrial genomes captured by Anchored Hybrid Enrichment (Lemmon et al. 2012), we describe the population genetic diversity and structure of *H. chrysoscelis* and *H. versicolor* as well as their phylogenetic relationships to their relative species. Additionally, to gain confidence in our conclusions, we employ multiple methods of model selection to directly test previously proposed hypotheses of gray treefrog evolution along with new hypotheses that emerged from this study. In short, the goals of our study were to: 1) ascertain the mode of polyploidization in *H. versicolor*; 2) determine the identity and number of ancestor(s) that gave rise to tetraploid *H. versicolor*; 3) determine the number of independent origins of polyploidy and the timing of any whole-genome duplication events; and 4) characterize the population structure, hybridization, and demographic history of the complex.

## Results

### Sequencing Summary

Using Anchored Hybrid Enrichment, we targeted and sequenced loci that were chosen to be sufficiently conserved at the target site for the present phylogenetic depth but divergent enough at the sequence flanks to make meaningful inference (Lemmon et al. 2012). We successfully sequenced the target from all capture pools with minimal individual failures, producing a final data set with 117 individuals (35 *H.* versicolor, 71 *H. chrysoscelis*, seven *H. avivoca*, and the three outgroup taxa). Across all individuals, the average number of raw reads sequenced per individual was 7,723,547 (range: 2,490,670–25,206,362) and the average number of reads per locus was 1,984 (range: 16–6,039). After orthology assessment and manual trimming, the total number of locus alignments was 385, and after removing paralogs identified in *H. chrysoscelis*, we had a final number of 244 alignments. The average locus lengths for the 244 loci used were 1,380–1,383 sites depending on the data set. The amount of missing data for nuclear data sets ranged from 2.6% to 2.8%.

The concatenated nuclear data sets consisted of 374,071–374,891 sites depending on the data set, which included between 10,568 (2.83%) and 14,958 (3.99%) informative sites (table 1). Single-nucleotide polymorphism (SNP) extraction recovered 8,683 SNPs that were each flanked by five monomorphic sites with no missing data across *H. chrysoscelis* and *H. versicolor*. Whole mitochondrial genomes were recovered from all individuals sampled. The total number of sites recovered for the mitochondrial genome was 15,834, with 2,715 informative sites and 2.6% missing data.

**Table 1.** Summary Table of Genetic Data for the Analyses from Figure 1.

Analysis	Data Set Number	*N* Ind.	*N* Sites Concat.	*N* Var. Sites	*N* Inf. Sites	Avg. Locus Lengths	% Miss. Data
Full data set	1	152	374,345	26,852	14,950	1,381	2.7
*Hyla chrysoscelis*	2	82	374,891	22,164	11,982	1,383	2.8
VERS MAX and *H. chrysoscelis*	3	117	374,891	26,011	14,355	1,383	2.7
VERS MAX and *H. chrysoscelis*	4	117	374,891	23,896	12,924	1,383	2.7
VERS MAX	5	46	374,107	23,121	12,507	1,380	2.6
VERS MIN	6	46	374,071	20,056	10,568	1,380	2.6
Mitochondrial genome	NA	117	15,834	3,947	2,715	NA	2.6

Note.—*N* sites concatenated is the maximum length of the alignment for that specific analysis.

### Polyploid Data Processing

Both ploidy assessment methods nQuire (Weiß et al. 2018) and our own PloidyPal came to the same conclusions for each sample and identified three previously labeled *H. versicolor* that were assessed as diploids (supplementary fig. 1, Supplementary Material online). Upon a further review of the field notes, MP370 and MP717 were mislabeled after collection, and we then relabeled these samples as *H. chrysoscelis*. There were no field notes for the third misidentified sample (MP676), and that sample was therefore removed from the data set. Additionally, all three samples of unknown ploidy—ECM3053, ECM4330, and ECM4466—were identified as diploids by both methods and therefore labeled as *H. chrysoscelis* for further analyses.

Pairwise genetic distances between the average *H. chrysoscelis* and each *H. versicolor* sample in this study, separated into putative subgenomes defined as all phased alleles with the minimum (MIN) or maximum (MAX) pairwise genetic distance from the average *H. chrysoscelis* allele, demonstrate that on average *H. versicolor* is genetically very similar to *H. chrysoscelis*, with individual MAX subgenomes not exceeding a distance of 0.5% (supplementary fig. 2, Supplementary Material online). In comparison, *H. avivoca*, the sister species to the complex has an average distance of 1.23% to *H. chrysoscelis.* These results suggest that if *H. versicolor* were an allopolyploid derived from *H. chrysoscelis* hybridizing with an extinct species, at the very least the extinct species would have to have been a closer relative to *H. chrysoscelis* than *H. avivoca*, or that heterosomic inheritance in *H. versicolor* and/or hybridization between *H. versicolor* and *H. chrysoscelis* has eroded the genomic signal of a more distant relationship. Although we do see a difference between the putative MIN/MAX subgenomes in *H. versicolor* (average MAX = 0.40%, MIN = 0.27%), that difference is relatively small and less than the average distance of any individual *H. chrysoscelis* to the average *H. chrysoscelis* across all individuals (0.24%). Additionally, measurements of within subgenome diversity demonstrate a greater diversity in the MAX subgenome, likely because restricting the MIN subgenome to the minimum difference from the average *H. chrysoscelis* restricts sequences to similarity in a single radial direction in sequence similarity/dissimilarity space, whereas the MAX subgenome sequences can be dissimilar in any radial direction (supplementary table 3, Supplementary Material online).

Finally, it should be noted that as with all steps in processing and generating genomic data, our ability to phase for separating MIN/MAX sequences is limited by the data quality and sequence diversity. Using paired-end 150-bp sequencing, any two SNPs farther than 300 bp from each other are unable to be phased because no single read will overlap both SNPs. Although a rough estimate as SNPs are not exactly uniform in distribution across a locus, this translates to an average SNP density of 0.0067 SNPs per site. In *H. versicolor*, the median SNP density of an individual sample’s MIN/MAX sequence was lower than this threshold at 0.0032 SNPs per site, with only 26 of the 244 final loci surpassing the 0.0067 density threshold. Ultimately, these results suggest there is little difference between potential subgenomes, and few if any target loci would able to be phased at complete accuracy across all individuals with any amount of sequencing coverage. In order to not artificially select for the most divergent loci and bias results (Huang and Knowles 2016), amelioration of this issue requires the use of longer-read sequencing technologies.

### Nuclear Phylogenetic Relationships

**Fig. 1.**
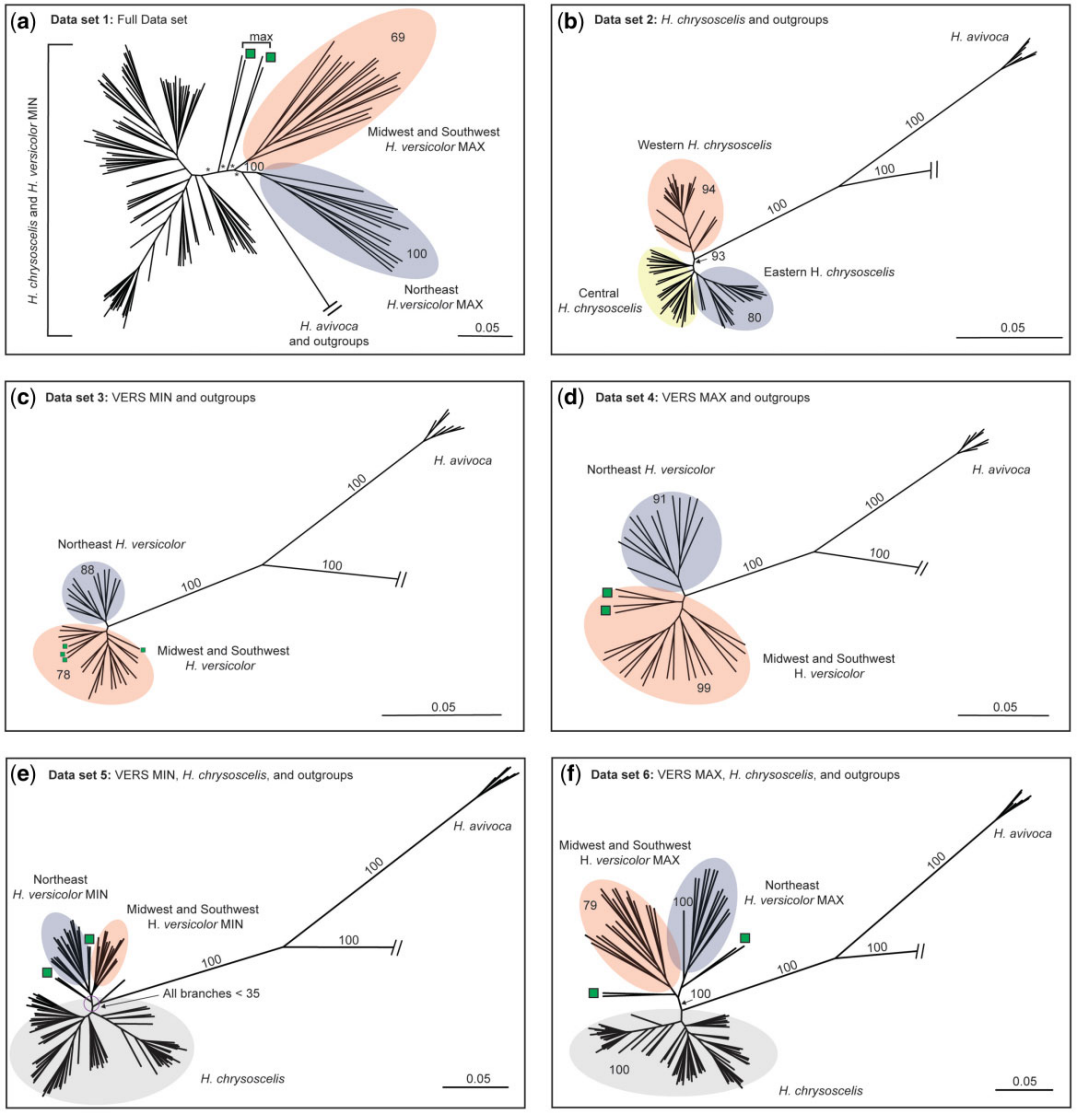
RAxML phylogenies for concatenated alignments across 244 AHE loci for the full data set (*a*) and the five subsets (*b–f*) analyzed. Colored ellipses highlight clades of interest. Bootstrap values are only reported on branches informative for this study (full trees with tip labels and bootstrap support at all nodes can be found in the figshare repository). Stars represent bootstrap support <50. Green squares indicate individuals that suggest potential past connectivity of *Hyla versicolor* NE and SW lineages (see Discussion). Scale bar and branch lengths represent substitutions per site. Outgroup relationships shown in supplementary figure 3, Supplementary Material online.

Phylogenetic analyses of nuclear data from RAxML provided support that *H. versicolor* may harbor alleles from an unsampled, apparently extinct population or species (fig. 1*a*, *e*, and *f*, *H. versicolor* sequences outside of the *H. chrysoscelis* clade). In addition, these data showed evidence of clear genetic breaks across geography for both *H. chrysoscelis* and *H. versicolor*. Individual gene trees exhibited low bootstrap support, likely due to the low information content at each locus at this shallow phylogenetic scale. Concatenated analyses of all subset nuclear data sets, however, generally had high bootstrap support for informative branches. Conversely, low branch bootstrap support for some analyses may also be informative for identifying a lack of variation between *H. versicolor* and *H. chrysoscelis* genes (e.g., fig. 1*e*).

**Fig. 2.**
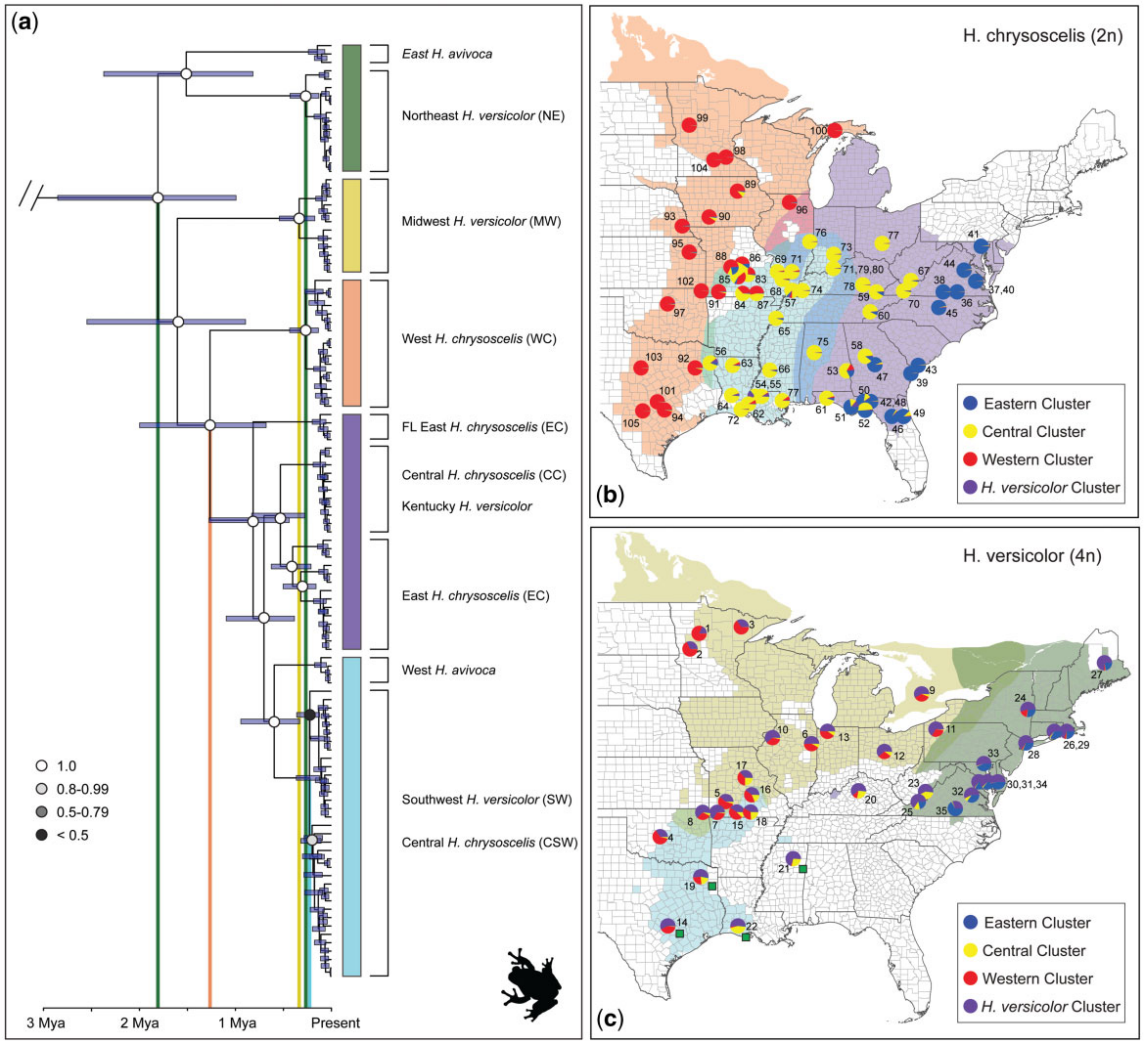
(*a*) Dated whole-genome mitochondrial phylogeny from BEAST analysis of all ingroup and outgroup taxa (*Hyla andersonii, H. arenicolor*, and *H. femoralis* not shown, see supplementary fig. 5, Supplementary Material online). Colored bars right of the phylogeny highlight mitochondrial clades. Circle shading on nodes represent posterior values for those nodes and are only reported for branches informative for this study (full tree with all tip and node labels supplementary fig. 4, Supplementary Material online). From left to right, vertical bars show mean timing of coalescence for: 1) *H. avivoca*, *H. versicolor*, and *H. chrysoscelis*; 2) Eastern/Central *H. chrysoscelis* and Western *H. versicolor*, 3) all MW *H. versicolor*; 4) all NE *H. versicolor*; and 5) all SW *H. versicolor* and the Central *H. chrysoscelis* with which they share a monophyletic mitochondrial clade. (*b*) Distribution map of *H. chrysoscelis*. Background colors indicate putative ranges of mitochondrial lineages, circles represent nuclear STRUCTURE results for *H. versicolor* and *H. chrysoscelis* analysis at *K* = 4 with one SNP per locus. *K* = 4 is visualized here because this analysis most reflected the topology from our phylogenetic analysis (fig. 1*b*), did not include additional clusters that were uninformative, and overall was most useful for visualization of the complex population structure. Numbers correspond to Map ID number in supplementary table 1 and supplementary figures 6 and 7, Supplementary Material online. Green squares next to Texas, Louisiana, and Tennessee samples correspond to individuals that had ambiguous relationships in RAxML analyses. (*c*) Distribution map of *H. versicolor*. Background colors indicate putative ranges of mitochondrial lineages, circles represent STRUCTURE results for *H. versicolor* and *H. chrysoscelis* analysis at *K* = 4 with one SNP per locus. Background colors indicate putative ranges of mitochondrial lineages, circles represent nuclear STRUCTURE results for *H. versicolor* and *H. chrysoscelis* analysis at *K* = 4 with one SNP per locus. Numbers correspond to Map ID number in supplementary table 1 and supplementary figures 6 and 7, Supplementary Material online.

The concatenated analysis using the full data set (data set 1; fig. 1*a*) recovered a topology similar to those of subset analyses (data sets 2–6; fig. 1*b*–*f*), but ultimately had low bootstrap support values at most branches. The two subset analyses with both *H. versicolor* and *H. chrysoscelis* (fig. 1*e* and *f*) resulted in topologies with high bootstrap support that provide evidence that *H. versicolor* has a large proportion of alleles that came from a population or species that was sister to all extant *H. chrysoscelis*. Additionally, within the two subset analyses including MIN or MAX *H. versicolor* and outgroups only, the recovered pattern suggests extant *H. versicolor* is separated into two general clades—one eastern nuclear genetic lineage consisting of all sampled individuals from WV, VA, MD, NJ, NY, CT, ME, and one western nuclear genetic lineage consisting of all other sampled *H. versicolor* (fig. 1*c* and *d*). Consistent with previous work (Ralin and Selander 1979; Holloway et al. 2006), our analysis of *H. chrysoscelis* and outgroups identifies two clades within *H. chrysoscelis*, a Western clade and an Eastern + Central clade, with a monophyletic Eastern clade nested within the Central lineage (figs. 1*b* and 2*c*).

Additionally, other patterns from our nuclear phylogenetic analysis suggest a more complex evolutionary history than can be explained by simple bifurcations. Green squares on the phylogenies in figure 1 and map in figure 2*b* indicate *H. versicolor* individuals from Texas, Louisiana, and southwest Tennessee that were consistently placed on the phylogeny nearby but outside the clades expected from their geographic location. These patterns may be evidence of hybridization between *H. chrysoscelis* and *H. versicolor*—a conclusion reached by several other analyses in this study.

Finally, the taxonomic relationships of the outgroup taxa from our nuclear analyses demonstrate a unique topology that conflicts with previous estimates (supplementary fig. 3, Supplementary Material online) (Faivovich et al. 2005; Duellman et al. 2016). For all concatenated analyses, we recovered species relationships that separate *H. versicolor* and *H. chrysoscelis* from *H. avivoca*, *H. andersonii*, *H. arenicolor*, and *H. femoralis* as two monophyletic clades with high bootstrap support. Within the outgroup clade, *H. avivoca* forms a monophyletic clade separate from the other outgroup taxa, and within *H. avivoca*, we recover a topology that separates East (AL, GA, TN) and West (MS, LA) *H. avivoca* with high support. However, the relationships between *H. avivoca* and ingroup or outgroup taxa is dependent on root placement of the present phylogeny. Midpoint-rooting places *H. avivoca* as sister to all other outgroup taxa (supplementary fig. 3*a*, Supplementary Material online), but because of the decreased pairwise genetic distance of *H. avivoca* from *H. chrysoscelis* in comparison to other outgroup taxa (supplementary fig. 2, Supplementary Material online), this rooting scheme may be spurious, and rooting on any other outgroup branch (not including within *H. avivoca*) places *H. avivoca* as sister to all *H. versicolor* and *H. chrysoscelis* (supplementary fig. 3*b*, Supplementary Material online).

### Mitochondrial Phylogenetic Relationships and Coalescent Timing

Whole mitochondrial genome analyses from BEAST recovered a topology similar to that found in the single-gene mitochondrial study of Ptacek et al. (1994), but our increased gene and taxon sampling improved phylogenetic estimates and clarified the origin of lineages (table 1; fig. 2*a*; supplementary fig. 4, Supplementary Material online). Five main clades were delineated: one containing *H. versicolor* only (Midwest, MW); one consisting of northeastern *H. versicolor* and eastern *H. avivoca* (Northeast, NE); one including western *H. avivoca*, southwestern *H. chrysoscelis* (CSW), and southwestern *H. versicolor* (Southwest, SW); a western *H. chrysoscelis* clade (West *chrysoscelis*, WC); and one paraphyletic group containing East *H. chrysoscelis* (EC), Central *H. chrysoscelis* (CC) and one *H. versicolor* from Meade County Kentucky (fig. 2*a*). All major branches had posterior probabilities of 1, apart from internal nodes within the SW/CSW clade where *H. versicolor* and *H. chrysoscelis* are not individually monophyletic.

The whole mitochondrial genome analysis for our outgroup taxa recovered species level relationships with high posterior probability, but a topology that conflicts with the topology estimated by our nuclear analyses (supplementary fig. 5, Supplementary Material online). Using mid-point rooting, we recovered a topology that places the ingroup (including *H. avivoca*) as sister to *H. andersonii*, and this clade is sister to *H. arenicolor*. Finally, this analysis delineates *H. femoralis* as sister to all other species used in this study.

We recovered mitochondrial coalescent timing estimates with narrow 95% credibility intervals for all nodes with a mean clock rate of 0.906% per lineage per million years. Date estimates of importance can be found in supplementary table 4, Supplementary Material online, but briefly, these estimates suggest coalescent timing of all identified *H. versicolor* lineages as sometime before 430 ka (NE: mean 0.262 Ma, 95% CI 0.125–0.426 Ma; MW: mean 0.338 Ma, 95% CI 0.131–0.430 Ma; SW: mean 0.223 Ma, 95% CI 0.120–0.360 Ma). This analysis placed the coalescent timing of all *H. chrysoscelis* before 1.99 Ma (mean 1.26 Ma, 95% CI 0.675–1.99 Ma).

### Population Genetic Analyses

To assess the population genetic structure of gray treefrogs, we used several analyses including STRUCTURE, genetic PCA, as well as investigated incomplete lineage sorting (ILS) in the complex. Within *H. chrysoscelis*, our STRUCTURE analysis across multiple *K* values suggest there are three distinct clusters representing Western, Central, and Eastern *H. chrysoscelis* (fig. 2*c* and supplementary figs. 6 and 7, Supplementary Material online). This result is consistent with our nuclear phylogenetic and whole mitochondrial genome phylogenetic results (fig. 1*b* and 2*a*), however the nuclear phylogeny places Eastern *H. chrysoscelis* nested within the Central lineage, and the mitochondrial phylogeny shows two divergent mitochondria segregating within the center of the Eastern/Central lineage’s range. Although individual *H. chrysoscelis* generally have a >90% identity match to a single cluster, some geographic locales contain several individuals with significant genetic contributions from neighboring or sympatric lineages, suggesting hybridization between *H. chrysoscelis* lineages in contact zones.

Within *H. versicolor*, all individuals show evidence of a unique genetic influence that is generally absent from *H. chrysoscelis* outside of minor frequencies (shown in purple, fig. 2*b* and supplementary figs. 6 and 7, Supplementary Material online). This pattern is consistent across several *K* values in both the single SNP per locus and all SNPs analyses. Interestingly, an additional analysis that included *H. avivoca* showed evidence that this distinct *H. versicolor* cluster might have descended from *H. avivoca* (purple cluster at *K* = 3–4, supplementary fig. 8, Supplementary Material online). This observation is further supported by the mitochondrial tree, which shows that eastern *H. avivoca* share a recent mitochondrial ancestor with NE *H. versicolor* (fig. 2*a*). Additionally, some support was found at *K* = 3 that alleles contributed to *H. versicolor* from *H. avivoca* were first integrated into the Eastern *H. chrysoscelis* genome. Although not conclusive, this pattern may suggest a single origin of *H. versicolor* from an ancestral population most closely related to Eastern *H. chrysoscelis*.

**Fig. 3.**
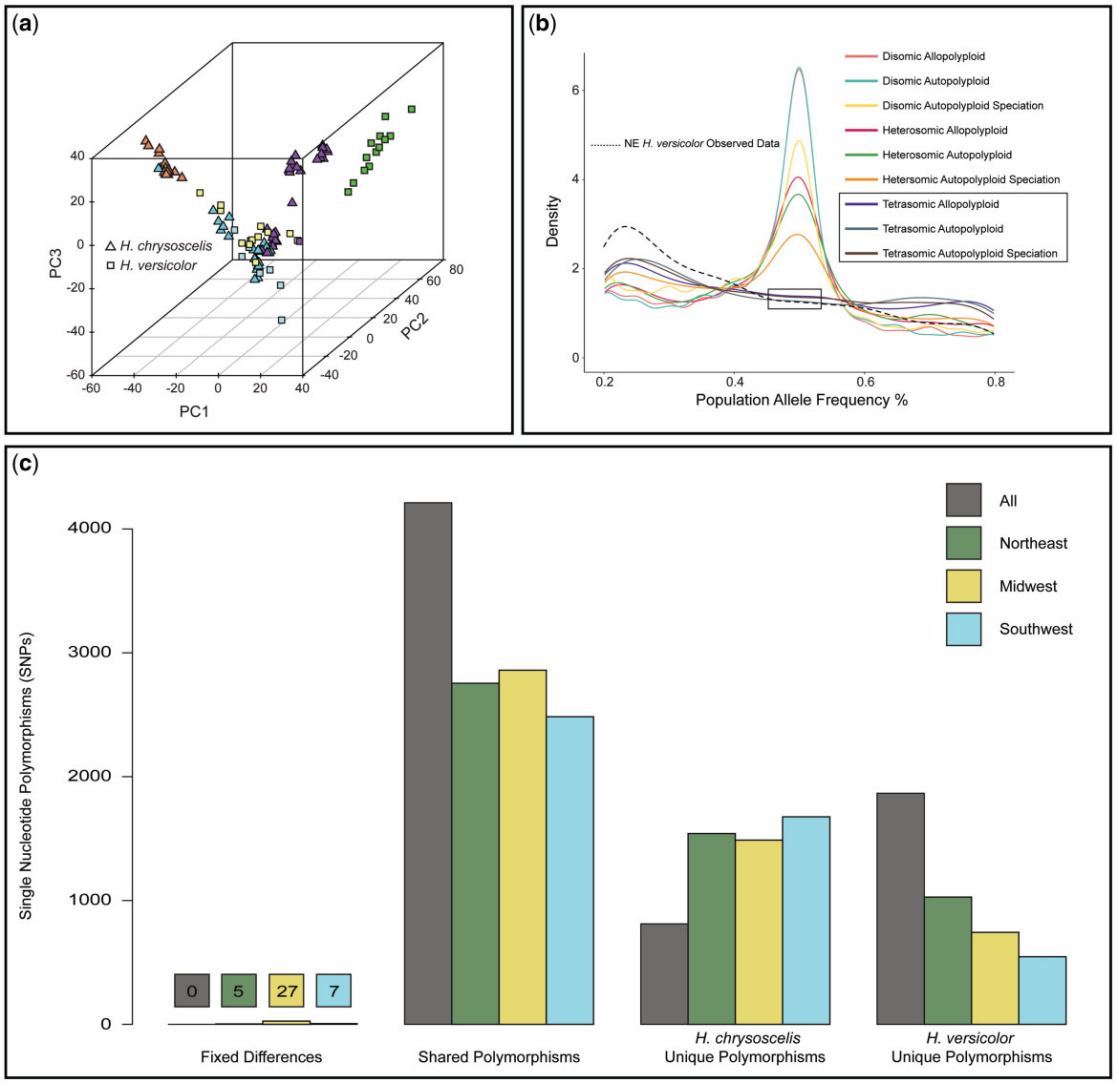
(*a*) Genetic PCA results for 8,683 SNPs across 244 loci. Squares and triangles represent *Hyla versicolor* and *H. chrysoscelis*, respectively. Fill color represents mitochondrial lineage and matches those presented in figure 2*a*–*c*. (*b*) Simulated and observed allele frequencies comparing nine speciation and inheritance mode combinations (one-way migration) to the observed allele frequencies of the NE *H. versicolor* lineage (shown as the dashed line). Simulations were done under a unidirectional migration model from the diploid to both A and B polyploid subgenomes. All heterosomic and disomic inheritance simulations produced allele frequencies with a significant number of alleles at 50% frequency, and tetrasomic inheritance simulations under all three polyploid speciation models did not produce a significant number of alleles at 50% frequency and were closest to the observed data. (*c*) SNPs with fixed differences between *H. chrysoscelis* and *H. versicolor*, polymorphisms shared between *H. chrysoscelis* and *H. versicolor*, polymorphisms unique to *H. chrysoscelis*, and polymorphisms unique to *H. versicolor*. Comparisons were conducted between all *H. chrysoscelis* and *H. versicolor*, as well as between all *H. chrysoscelis* and each *H. versicolor* mitochondrial lineage.

Additionally, we also found similar Western, Central, and Eastern clusters in *H. versicolor* as observed in *H. chrysoscelis*. Aside from the unique genetic influence in *H. versicolor* (purple cluster), other cluster proportions within individuals are remarkably similar to nearby *H. chrysoscelis* populations—especially so when *H. versicolor* and *H. chrysoscelis* are sympatric (fig. 2*c* and *b*). We found a similar pattern in our genetic PCA analysis (fig. 3*a*), with MW and SW *H. versicolor* being generally indistinguishable from Eastern and Central *H. chrysoscelis* across axes 1–3 (PCA axes 1–3 represent 4.8%, 3.2%, and 2.5% of the variability, respectively). Genetic PCA results also show NE *H. versicolor* as distinct from all other *H. versicolor* and *H. chrysoscelis* but most similar to Eastern *H. chrysoscelis*. The divergence of NE *H. versicolor* from MW and SW lineages is also seen at higher *K* values from our analysis that included *H. avivoca* (green cluster, supplementary fig. 8, Supplementary Material online.

**Fig. 4.**
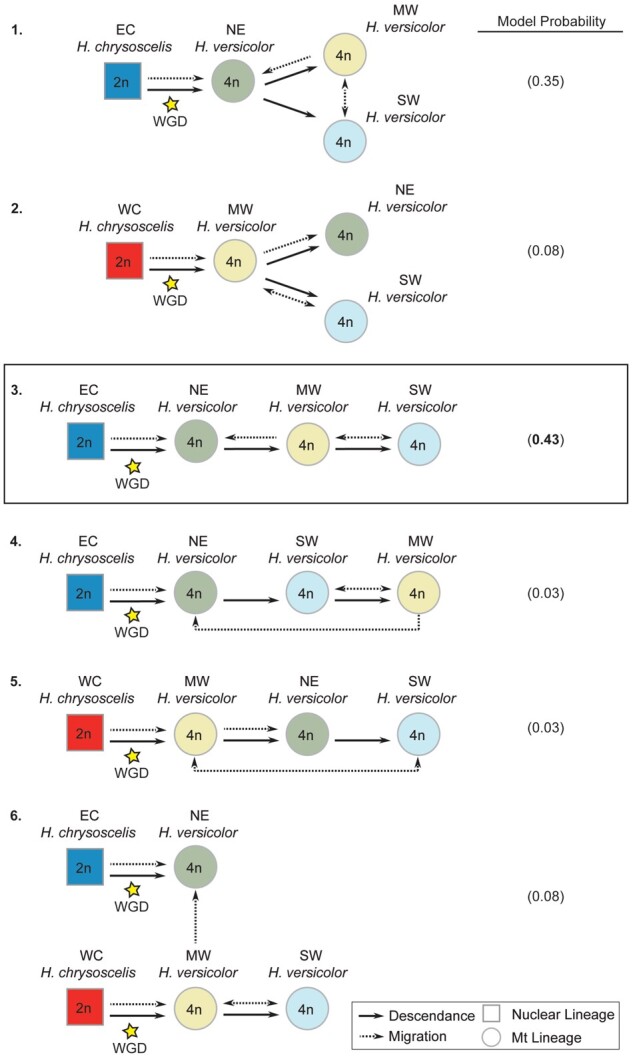
Models of *Hyla versicolor* descendance and migration used for the final migrate-n analysis and their model probabilities calculated using Bayes Factors from their Bezier approximation score. Boxed model demonstrates the model with the highest probability.

Our assessment of the distribution of SNPs demonstrates significant ILS between *H. chrysoscelis* and *H. versicolor* (fig. 3*c*). We found no fixed differences across the two species as a whole, and very few fixed differences between *H. chrysoscelis* and each individual *H. versicolor* lineage. These results also demonstrate the majority (>4,000) of SNPs we recovered represent shared polymorphisms between *H. chrysoscelis* and *H. versicolor*, indicating significant ILS in this complex. Finally, this assessment also shows greater nucleotide diversity within *H. versicolor* than in *H. chrysoscelis* when the two species are compared collectively, but the reverse relationship when comparing *H. chrysoscelis* to each *H. versicolor* lineage individually. The lineage level analysis also demonstrates a difference in nuclear diversity across lineages that support a NE origin and the stepping-stone model identified from our Migration and Descendance Model Testing Results (model 3; fig. 4).

### Migration and Descendance Model Testing

The first round of migration and descendance model testing using the population genetic model inference software migrate-n (Beerli 2006; Beerli and Palczewski 2010) suggest that NE *H. versicolor* originated from Eastern *H. chrysoscelis*, and that MW and SW *H. versicolor* are descendants of NE *H. versicolor*—providing support that *H. versicolor* arose from a single whole-genome duplication event (table 2). Indeed, the NE *H. versicolor* lineage was the only lineage that had a significant probability of being a descendant of any one *H. chrysoscelis* lineage, with descendance from Eastern *H. chrysoscelis* having the highest probability (0.47 model probability, table 2). For both the MW and SW *H. versicolor* lineages, the best supported model was one of descendance without current migration from the NE *H. versicolor* lineage (0.88 and 0.81 model probability, respectively). When we tested the final set of six models, however, we found higher support for a stepping-stone model, where MW *H. versicolor* descended from NE *H. versicolor*, and SW *H. versicolor* descended from MW *H. versicolor*. Importantly, the above model was not overwhelmingly supported (0.43 model probability; fig. 4, model 3), and a model of independent origination of MW and SW *H. versicolor* from NE *H. versicolor* also received considerable support (0.35 model probability; fig. 4, model 1). We found little support for any other models, including the model of multiple origins.

**Table 2.** Bezier Approximations of the Marginal Likelihood and the Model Probability from Migrate-N Descendance and Migration Analyses.

Marginal Likelihood			Model Probability			
Descendance Model	NE	MW	SW	NE	MW	SW
EC no migration	−117,680.5	−117,668.1	−117,668.1	0.00	0.01	0.01
EC migration	−117,664.6	NA	NA	0.47*	NA	NA
CC no migration	−117,697.1	−117,684.4	−117,684.1	0.00	0.00	0.00
CC migration	−117,665.7	−117,667.5	−117,666.2	0.14	0.01	0.07
WC no migration	−117,667.9	−117,710.0	−117,696.6	0.02	0.00	0.00
WC migration	NA	−117,666.6	−117,666.2	NA	0.03	0.06
NE no migration	NA	−117,663.2	−117,663.7	NA	0.88*	0.81*
NE migration	NA	−117,666.4	NA	NA	0.04	NA
MW no migration	−117,687.2	NA	−117,671.8	0.00	NA	0.00
MW migration	−117,665.2	NA	−117,666.3	0.25	NA	0.06
SW no migration	−117,665.9	−117,671.1	NA	0.12	0.00	NA
SW migration	NA	−117,666.4	NA	NA	0.04	NA

Note.—Each model (rows) of descendance and migration from the model lineage is evaluated for each *H. versicolor* mitochondrial lineage (columns). Starred numbers represent the best supported model probability.

When considering migration patterns, almost all models tested in our initial analyses had a higher log-likelihood when allowing for migration between any two lineages when appropriate. Specifically, all models improved when we allowed for unidirectional migration of diploid lineages into sympatric tetraploid lineages, and nearly all models improved when we allowed for migration between neighboring tetraploid lineages. The only model comparison that had worse support when allowing for migration was our MW *H. versicolor* descending from NE *H. versicolor* comparison, in which an asymmetric model that did not allow for migration from NE *H. versicolor* had higher support (table 2).

### Approximate Bayesian Computation Polyploid Speciation Model Testing

Our approximate Bayesian computation (ABC) analysis to evaluate the mode of polyploid formation suggests *H. versicolor* originated as an autopolyploid from an extinct *H. chrysoscelis* lineage, and that there is unidirectional migration from Eastern *H. chrysoscelis* into NE *H. versicolor* (model probability = 0.57; table 3). In addition, almost all model support is concentrated across both unidirectional migration models, with the allopolyploid unidirectional migration model also receiving a high level of support (model probability = 0.39). A linear discriminate analysis (LDA) of each of the six models shows that although models with different migration parameters can generally be distinguished in LDA space, when there is migration between diploids and tetraploids, autopolyploid and allopolyploid models become almost indistinguishable (supplementary fig. 9, Supplementary Material online). Importantly, however, our observed data (star in supplementary fig. 9, Supplementary Material online) are well within LDA space and therefore the priors used for our simulations. Our robustness assessment shows that our ABC analysis is able to distinguish allopolyploid and autopolyploid models when there is migration, but this assessment does not provide any additional confidence that the chosen model is the true model outside of the originally estimated probability (supplementary fig. 10, Supplementary Material online).

**Table 3.** Model Probabilities from our ABC Analysis under Different Polyploidization Mode and Migration Histories.

Formation	Inheritance	Migration History	Model Probability
Autopolyploid	Tetrasomic	No Migration	0.0002
Allopolyploid	Tetrasomic	No Migration	0.0002
Allopolyploid	Tetrasomic	Bidirectional AB	0.0124
Autopolyploid	Tetrasomic	Bidirectional AB	0.0250
Allopolyploid	Tetrasomic	Uni-directional AB	0.3940
Autopolyploid	Tetrasomic	Uni-directional AB	0.5682

Note.—Probabilities are summarized across 20 independent runs. AB distinction for some models refers to migration with the diploid and both tetraploid subgenomes.

**Fig. 5.**
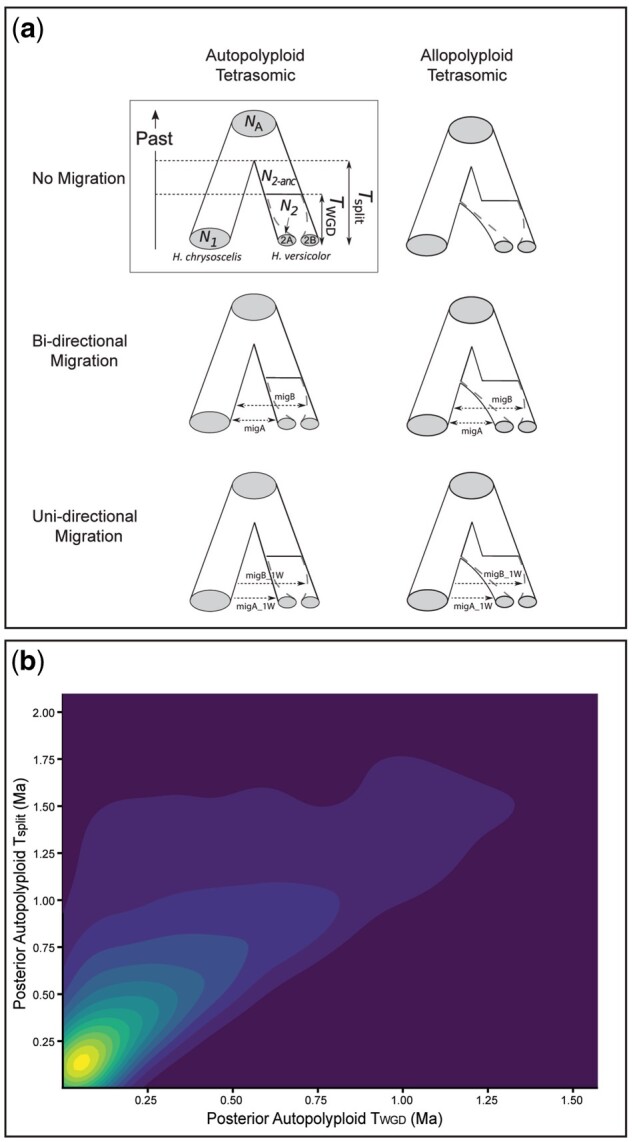
(*a*) The six polyploid speciation models and their parameters used in the ABC analysis (modified from Roux and Pannell [2015]). The model inside the square depicts the parameters used for creating each model. (*b*) 2D density plot of posterior distributions for *T*_split_ and *T*_WGD_ of the Autopolyploid Tetrasomic One-Way Migration model used in the ABC analysis. Times are in millions of years ago (Ma).

Posterior distributions for the best supported model suggest that the extinct lineage that led to the autopolyploid formation of *H. versicolor* split from Eastern *H. chrysoscelis* around 2.88×10^5^ years ago (peak distribution value [PDV]: 2.88×10^5^; 90% CI: 1.02×10^3^–3.22×10^6^), and that the whole-genome duplication event occurred 1.00×10^5^ years ago (PDV: 1.00×10^5^; 90% CI: 2.4×10^1^–1.84×10^6^) (fig. 5*b*; all population size and timing posteriors supplementary fig. 12, Supplementary Material online). Alternatively, under the second-best supported model, the peak posterior distribution value suggests much earlier *T*_split_ and *T*_WGD_ times at 4.19×10^6^ (PDV: 4.19×10^6^; 90% CI: 2.00×10^6^–8.11×10^6^) and 2.96×10^6^ (PDV: 2.96×10^6^; 90% CI: 9.07×10^5^–2.96×10^6^) years ago, respectively (supplementary fig. 12, Supplementary Material online). Though largely overlapping with the prior, the posterior values for the autopolyploid model are consistent with the coalescent times observed in our mitochondrial phylogenetic analysis (supplementary table 4, Supplementary Material online), whereas the posterior for *T*_split_ and *T*_WGD_ times under an allopolyploid model would predate the coalescent times of all *H. chrysoscelis* and *H. versicolor* from the same analysis (fig. 2*a*). Given that the nuclear clock rate we used to simulate the data was estimated given the coalescent times from our mitochondrial phylogenetic analysis, the dates estimated by the ABC analysis should be approximately within the estimated coalescent timing distribution, even if that estimate is incorrect. These results, in our view, add further support to an autopolyploid model, suggesting that for an allopolyploid model to be supported, the *T*_split_ and *T*_WGD_ times would need to be well outside of what is biologically reasonable.

### Site Frequency Spectra of Simulated Polyploid Models

To better understand the mode of inheritance in *H. versicolor* and its relation to polyploidization history and interploid hybridization, we compared the observed site frequency spectra to those simulated under different histories. Site frequency spectra at intermediate allele frequencies were similar across all simulated migration histories—suggesting that significant deviations from tetrasomic inheritance should be detectable as an excess of observed alleles at 50% frequency in the population regardless of migration history, given some differentiation has occurred between subgenomes since WGD for autopolyploid species with disomic or heterosomic inheritance (fig. 3*b* and supplementary fig. 11, Supplementary Material online). The observed frequencies for all mitochondrial *H. versicolor* lineages were nearly identical and most similar to those observed by the simulated tetrasomic models. Previous segregation studies have suggested that *H. versicolor* is not entirely tetrasomic, with some genes segregating in a disomic fashion (Danzmann and Bogart 1982, 1983). Importantly, the simulated heterosomic models are produced by randomly choosing some number of disomic loci from a uniform prior—meaning the density histogram of allele frequencies reflects a genome with ∼50% disomic inheritance. Genomes with only one or a few chromosome sets forming bivalents may be indistinguishable from completely tetrasomic genomes without significant sampling across the genome. Thus, it is possible there is some level of disomic inheritance in the *H. versicolor* genome; however, the overwhelming pattern of chromosomal inheritance is tetrasomic, and we are confident in eliminating heterosomy and disomy from our chosen set of models.

## Discussion

**Fig. 6.**
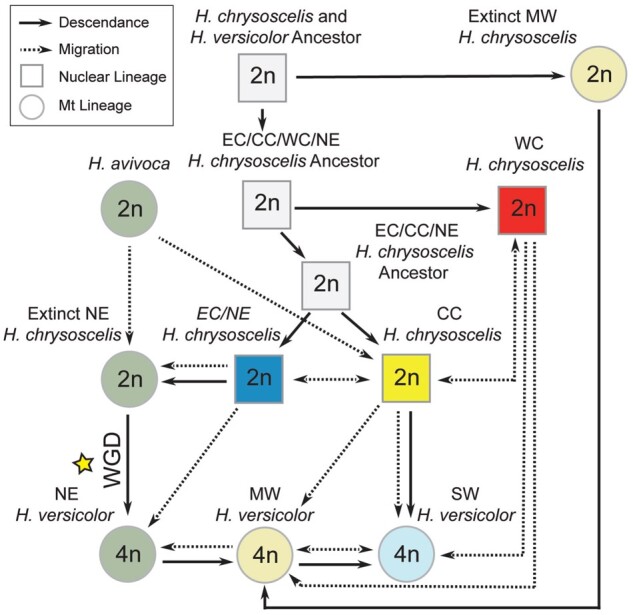
Network model of speciation and evolution proposed from the results of this study. Background colors of circles represent mitochondrial lineages apart from ancestral lineages which are colored white. Solid arrows point to descendants of a given lineage. Dashed lines demonstrate current migration for existing lineages or past migration for extinct lineages, with arrows indicating the direction of migration. Circles represent populations identified by their mitochondrial genome (*Hyla versicolor, H. avivoca*, and extinct lineages), squares represent populations defined by their nuclear genome (*H. chrysoscelis*). The yellow star indicates the proposed single whole-genome duplication event that led to the formation of *H. versicolor*.

As evidenced here, model selection is an important tool for testing alternative hypotheses to disentangle the complex relationships in polyploid systems. Ours is the first study that has not relied solely on descriptive methods to delineate the history of gray treefrogs, and in its totality, the evidence presented above largely disagree with the previous conclusions from Holloway et al. (2006). Specifically, rather than supporting multiple allopolyploid origins, our study instead suggests that *H. versicolor* most likely formed via a single autopolyploid whole-genome duplication event, and that current lineages of *H. versicolor* are a result of repeated backcrossing with extant and extinct lineages of *H. chrysoscelis* (fig. 6). Several lines of evidence from our study also corroborate previous observations that *H. versicolor* hybridizes with *H. chrysoscelis* when they are sympatric (Gerhardt et al. 1994; Bogart et al. 2020) and the hypothesis that *H. chrysoscelis* alleles regularly introgress into the *H. versicolor* genome to create high levels of heterozygosity (Ralin and Selander 1979; Bogart and Bi 2013). Finally, multiple results from this study suggest that the sister species to the complex, *H. avivoca*, has played an ongoing role in the genetic history of this group as well.

### Mode of Polyploid Formation

Multiple lines of evidence suggest that *H. versicolor* is an autopolyploid that formed via a single whole-genome duplication event from an extinct NE *H. chrysoscelis* lineage that was genetically most similar to present day Eastern *H. chrysoscelis*. Autopolyploidy is indicated through our observation of intermediate allele frequencies reflective of simulated tetrasomic polyploid models, the pairwise genetic distances of *H. versicolor* to *H. chrysoscelis* individuals, and our results from ABC polyploid speciation model selection (fig. 3*b*; supplementary fig. 2, Supplementary Material online; table 3). Although an allopolyploid model was also moderately supported from our ABC analysis, the posterior estimates of *T*_split_ and *T*_WGD_ times under this model (supplementary fig. 12, Supplementary Material online) fall well outside the times estimated by our mitochondrial coalescent analysis (supplementary table 4, Supplementary Material online) that were used to estimate the nuclear clock rate for the ABC analysis. Autopolyploidy is also most consistent with the phenotypic and ecological similarity of *H. versicolor* and *H. chrysoscelis*. The two species are indistinguishable apart from their calls and the associated female responses, and while there is some evidence to suggest the two species partition their calling sites when sympatric, no other study has been able to identify a reliable ecological or phenotypic difference between *H. versicolor* and *H. chrysoscelis* that is not a direct consequence of polyploidy itself (e.g., increased cell size) (Ralin 1968; Kamel et al. 1985; Ptacek 1992).

The Holloway et al. (2006) study is the only study of which we are aware that makes a definitive conclusion of allopolyploidy in *H. versicolor*. Here, we draw similar conclusions to those outlined by Bogart and Bi (2013)—namely that the Holloway et al. (2006) observation of alleles in *H. versicolor* not present in extant *H. chrysoscelis* is also compatible with an autopolyploid origin of *H. versicolor*. This conclusion suggests rather that *H. chrysoscelis* has long been a widespread species across eastern North America, and some historical populations that contributed genes to *H. versicolor* are now extinct. However, an additional result from our study could also suggest allopolyploidy—the phylogenetic separation of MAX subgenomes from a MIN and *H. chrysoscelis* clade shown in figure 1*a* despite the majority of loci having tetrasomic inheritance. From the totality of evidence presented in this study, we do not believe this is due to allopolyploidy, but rather the high levels of ILS observed (fig. 3*c*), the possibility that some loci are inherited disomically (e.g., Danzmann and Bogart 1982, 1983), recent gene flow from diploids into tetraploids (table 3), and the overall effect grouping alleles into two classes by divergence has on phylogenetic assessment. Finally, as Bogart and Bi (2013) and Bogart et al. (2020) suggest but was originally proposed by Bogart and Wasserman (1972), our results are not inconsistent with the hypothesis that *H. versicolor* formed via a triploid intermediary (i.e., triploid bridge). Given the highly reticulate nature of this complex, however, we are unable to provide any evidence to support or reject their hypothesis outside of our support for autopolyploidy.

### Identity of Ancestors and Number of Origins

Our results suggest that *H. versicolor* arose from a single whole-genome duplication event of a now extinct lineage of *H. chrysoscelis* that was most genetically similar to present day Eastern *H. chrysoscelis*. A single origin is supported by our migrate-n model testing, with support for descendance from any *H. chrysoscelis* population only occurring for the NE *H. versicolor* model (table 2). Similarly, our other single-lineage migrate-n model tests demonstrate high support that MW and SW *H. versicolor* are descendants of NE *H. versicolor* (table 2). However, a final model test suggests that SW *H. versicolor* is not a direct descendant of NE *H. versicolor*, but rather a descendant of MW *H. versicolor* populations which had descended from NE *H. versicolor* (fig. 4, model 3). This model is further supported from our ILS assessment, which demonstrates decreased nucleotide diversity in MW *H. versicolor* compared with NE *H. versicolor*, and decreased nucleotide diversity in SW *H. versicolor* compared with MW *H. versicolor*.

A single origin is also supported by the presence of a unique genetic cluster found in all *H. versicolor* from our STRUCTURE analyses that may be derived from *H. avivoca* through an intermediate Eastern *H. chrysoscelis* conduit (supplementary fig. 8, Supplementary Material online). Mitochondria from the NE *H. versicolor* clade are most closely related to eastern *H. avivoca* mitochondria (fig. 2*a*), but the migrate-n model tests (table 2), pairwise genetic distance of *H. versicolor* and *H. chrysoscelis* (supplementary fig. 2, Supplementary Material online), nuclear phylogenetic analysis (fig. 4), and genetic PCA (fig. 3*a*), all suggest NE *H. versicolor* descended from the much more recently derived Eastern *H. chrysoscelis* population. Incomplete lineage sorting (ILS) is not likely to produce this pattern, but hybridization can, if some Eastern *H. chrysoscelis* population hybridized with *H. avivoca* prior to the formation of *H. versicolor*. This conclusion would also explain why we see clustering of *H. avivoca* with Eastern *H. chrysoscelis* and NE *H. versicolor* at *K* = 3 (supplementary fig. 8, Supplementary Material online). The results reported here largely corroborate the Romano et al. (1987) study that suggested a single origin, but this study suggests an origination from NE *H. versicolor*, whereas their best estimate of the location of origin for *H. versicolor* was somewhere in the central part of the range (i.e., MW *H. versicolor*).

Although the bulk of the evidence outlined in this study best supports a single origin of *H. versicolor*, some aspects of this study instead could provide support for multiple autopolyploid origins of *H. versicolor*. First, our mitochondrial phylogenetic analyses confirm that in addition to an extinct population of NE *H. chrysoscelis*, there was at least one more population of *H. chrysoscelis* that is now extinct and whose genome is now at least partially retained in MW *H. versicolor* (fig. 2*a*). Second, MW and NE *H. versicolor* are relatively genetically distinct, and the coalescent times of MW and NE *H. versicolor* ancestors are nearly identical. However, the only direct test for the origins of polyploidy overwhelmingly support a single origin (fig. 4 and table 2). While future, more sophisticated analyses may suggest different conclusions than our own, patterns alone can be misleading and should not be solely relied on for drawing conclusions in polyploid systems.

### Complex Population Origin, Composition, Timing, and Hybridization

Despite the inherent postzygotic isolating mechanisms of interploid hybridization and previous work demonstrating strong (but not complete, see Gerhardt et al. [1994]) prezygotic isolation between diploids and tetraploids (Keller and Gerhardt 2001; Tucker and Gerhardt 2012), the totality of evidence from this study suggests that the North American gray treefrogs are a highly reticulate group with current ongoing exchange of alleles across polyploid and diploid lineages. Indeed, only one line of evidence from this study does not support the conclusion that any two lineages will hybridize whenever they come into contact—regardless of ploidy (migrate-n analysis suggests NE *H. versicolor* is not contributing migrants to MW *H. versicolor*, table 2). Although rare, triploids have been found in multiple sites where the two species are sympatric (Wiley et al. 1992; Gerhardt et al. 1994; Bogart and Bi 2013), and Gerhardt et al. (1994) observed roughly 3% of all amplexed pairs across six ponds were diploid-tetraploid mismatings. The strong evidence of *H. chrysoscelis* to *H. versicolor* gene flow from this study supports recent findings from Bogart et al. (2020) that demonstrated evidence of local gene flow between the two species, and our work in conjunction with previous observations of natural triploids strongly suggest gene flow across ploidies appears to occur range-wide where *H. versicolor* and *H. chrysoscelis* are sympatric. Furthermore, model support from our ABC analysis and the lack of the *H. versicolor* specific cluster in sympatric *H. chrysoscelis* populations provides strong evidence that this introgression is unidirectional with diploids contributing alleles to tetraploids but not vice versa. Finally, our ILS assessment demonstrates a similar pattern to the parallel allele frequencies reported in Ralin et al. (1983) and Romano et al. (1987), but our results suggest the parallel pattern they observed were likely due to gene flow from *H. chrysoscelis* into *H. versicolor*, rather than parallel evolution.

As evidenced here, this reticulate history has led to three general lineages of *H. versicolor* that are distinguished by their nuclear and mitochondrial genomic compositions. Results from our mitochondrial divergence date estimation and the posterior distribution from our ABC analysis places the WGD and origin of *H. versicolor* from extinct NE *H. chrysoscelis* as occurring sometime before 426 ka, with the best estimates from the mitochondrial phylogenetic and ABC analyses at 262 and 100 ka, respectively. These dates place the WGD event on either side of the Illinois glacial period (∼190–130 ka, Curry et al. 2011) and agrees with the previous Pre-Wisconsin estimates (roughly 150 ka, Blair 1965; Maxson et al. 1977; Ralin and Selander 1979). Coalescent times for MW *H. versicolor* from our mitochondrial phylogenetic analysis largely overlap with the NE *H. versicolor* coalescent time, suggesting that backcrossing of NE *H. versicolor* with the extinct MW *H. chrysoscelis* to form the MW *H. versicolor* lineage occurred shortly after the initial WGD event. Regarding SW *H. versicolor*, there is not a monophyletic mitochondrial clade for SW *H. versicolor* individually that allows for a date of the initial backcrossing of that group. However, evidence from this study suggests this lineage is the youngest of three major *H. versicolor* lineages. It is unclear whether the lack of monophyly is a result of mitochondria consistently migrating from *H. chrysoscelis* to SW *H. versicolor* or that differences in mitochondrial genomes between *H. chrysoscelis* and *H. versicolor* here are so small as to preclude phylogenetic resolution—though there is no evidence of mitochondrial introgression in MW and NE *H. versicolor* despite strong evidence of hybridization between *H. chrysoscelis* and *H. versicolor* in these populations. We do however see one other case of recent mitochondrial introgression in *H. versicolor*, wherein a single, highly disjunct population of *H. versicolor* in Meade county Kentucky has integrated novel mitochondria from Central *H. chrysoscelis* (CC) into at least one individual (Ind. 20; supplementary table 1, Supplementary Material online). Although it is possible this disjunct population of *H. versicolor* is from a unique WGD event, the presence of *H. versicolor* specific clusters from STRUCTURE as well as our RAxML concatenated phylogenetic analyses instead suggest this population became separated from already established *H. versicolor* populations (figs. 1*c*, 1*d*, 1*f*, and 2*b*; supplementary fig. 8, Supplementary Material online).

Results from this study suggest there are three lineages of *H. chrysoscelis* that each encompass large geographic areas across eastern North America. Although previous studies have recognized Eastern and Western *H. chrysoscelis* lineages with populations on the periphery of the two having an intermediate genetic makeup, our study instead suggests that intermediate *H. chrysoscelis* are a separate, paraphyletic Central *H. chrysoscelis* lineage that encompasses a monophyletic Eastern *H. chrysoscelis* lineage (figs. 1*b* and 2*c*), and that the combined monophyletic clade of Eastern and Central *H. chrysoscelis* is sister to Western *H. chrysoscelis*. There is also evidence for ongoing hybridization between the lineages that overlap, particularly between Eastern and Central *H. chrysoscelis* in the Florida panhandle and between Central and Western *H. chrysoscelis* in southern Missouri. The case of hybridization in southern Missouri is particularly interesting, as our results suggest two diploid and two tetraploid lineages are in contact and hybridizing in this area.

### Hyla Versicolor in Comparison to Other Polyploid Systems

Though there are significant barriers to introgression across species of different ploidies, the high levels of hybridization observed in this study are not unusual. Among plants, hybridization between species and populations of different ploidies appears to occur regularly (Soltis PS and Soltis DE 2009; Alix et al. 2017). In anurans, the complexes of *Phyllomedusa tetraploidea*, *Odontophrynus americanus*, and *Bufo viridis* show strong evidence that hybridization between tetraploids and diploids regularly produces triploid individuals (Stöck et al. 2002, 2005, 2010; Brunes et al. 2010, 2015; Grenat et al. 2018), and there is some evidence that several populations of the latter complex are entirely composed of hybrid triploids that are now sexually reproducing (Stöck et al. 2002, 2012). Similarly, the Australian *Neobatrachus* complex is composed of several autotetraploid and diploid species who have each likely hybridized with multiple diploid and tetraploid congeners several times since the initial polyploids were formed (Mable and Roberts 1997; Novikova et al. 2020). In *Xenopus*, though there are no extant diploids with which polyploids can hybridize, polyploid species have regularly hybridized in the past, and this hybridization has resulted in the evolution of species with elevated ploidy levels ranging from 4*n* to 12*n* (Evans 2008). Outside of bisexual polyploid amphibians, in more unique systems such as the unisexual *Ambystoma* and the hybridogenetic *Rana esculenta* complexes, hybridization is a de facto requirement for their existence. As suggested by Bogart and Bi (2013), hybridization between animal polyploids and with their lower-ploidy progenitors appears to confer an evolutionary advantage to polyploid lineages and facilitate their persistence. The extensive hybridization in the *H. versicolor* complex outlined here, though not direct evidence of this claim, provides further justification for its scholarly pursuit.

Finally, an important result from this study was the support for a singular origin of *H. versicolor.* Multiple origins are considered common in polyploidy, with some suggesting this is the rule rather than the exception (Soltis et al. 2004). Although this claim may be supported given the current synthesis of polyploid research, results from this study suggest studies concluding multiple origins that do not explicitly test this alongside alternative hypotheses should be re-evaluated (see also, Arnold et al. [2015]). Using this system as an example, the presence of several paraphyletic mitochondrial lineages associated with unique allelic variants was previously used as evidence that *H. versicolor* originated independently multiple times (Ptacek et al. 1994; Holloway et al. 2006), and similar evidence appears to be consistently used to support multiple origins in other systems (Segraves et al. 1999; Soltis et al. 2004, 2014). Our results suggest that simply the observation of patterns in the data that appear to support multiple origins of a polyploid species is in itself insufficient evidence for multiple origins, and that for systems where there are patterns that support multiple origins, explicitly testing this hypothesis is necessary before drawing any definite conclusions.

## Conclusions

In the present study, we have outlined the evolutionary history of *H. versicolor* and *H. chrysoscelis* in relation to polyploid formation as suggested by the data and analyses at hand. Although any single method of analysis in polyploid systems is subject to limitations, our work demonstrates multiple lines of evidence, along with direct model testing of polyploid histories, is necessary to uncover the evolutionary origins of a polyploid complex. Our study has addressed several key questions but also identified several puzzling new patterns in this complex that merit additional research. Although extant examples of polyploids are rare in animals compared with other groups such as angiosperms, polyploidy and gene/chromosomal duplications have undoubtedly played a major role in the evolutionary history of many animal groups (Otto and Whitton 2000; Gregory and Mable 2005; Blomme et al. 2006). Polyploid evolution in animals, however, has received little attention compared with the body of work in plants. Here, we demonstrate with the gray treefrog complex that animal polyploids can provide intriguing systems with which to answer important questions regarding the origins of polyploidization and the consequences of this process for diversification and speciation.

## Materials and Methods

### Polyploid Data Processing

To create species trees for polyploid complexes when the mode of polyploid formation is allopolyploid or otherwise unknown, whether using quartet-based or concatenated methods, it is first necessary to assign alleles to individual subgenomes. To begin this subgenomic assignment, alleles were phased using the allele phaser described in Pyron et al. (2016, but see supplementary methods, Supplementary Material online). Initially, all samples (35 *H. versicolor* and 71 *H. chrysoscelis*) were phased for four alleles, and specimen ploidy validation was conducted using our R package PloidyPal (www.github.com/wbooker/PloidyPal, last accessed November 11, 2021). The package PloidyPal was developed for the present study and uses pairwise genetic distances of the four phased alleles to determine the differential signal present from a known ploidy training sample (i.e., polyploids and diploids that are allopatric or that were otherwise confirmed using acoustic data). This signal is then used to predict ploidy for unknown individuals (full outlining of the algorithm can be found in the package README). To confirm our ploidy assessment, we also used the program nQuire (Weiß et al. 2018) to assess the ploidy of all specimens. nQuire takes a different approach to ploidy estimation by utilizing the distribution of raw sequences mapped to reference sequences. After this two-step process, samples were phased as their determined ploidy. Paralogs present in diploids that were identified during orthology assessment were then removed from any downstream analyses to prevent spurious assignment of diploid-tetraploid orthologs.

Similar to St. Onge et al. (2012), haplotypes of *H. versicolor* were then assigned to one of two putative subgenomes for phylogenetic analysis. Because we know *H. chrysoscelis* (or its recent ancestor) is at least one of the progenitors of *H. versicolor*, we calculated pairwise genetic distances of all *H. versicolor* haplotypes to the consensus *H. chrysoscelis* sequence and assigned the haplotype with the smallest distance to the VERS MIN subgenome (*H. chrysoscelis* subgenome) and the greatest distance haplotype to the VERS MAX subgenome (putative other subgenome). The intermediate two haplotypes in tetraploids were discarded for phylogenetic analyses. We realize this MIN/MAX characterization might predispose the phylogenetic analyses against autopolyploidy, but a variety of other methods we employed are not subject to this bias or usage of MIN/MAX delimitation and will help us understand the effect and limitations of this approach.

### Nuclear Phylogenetic Analyses

To reconstruct the nuclear evolutionary history of the gray treefrog complex, we estimated phylogenies (including sequences 35 *H. versicolor*, 71 *H. chrysoscelis*, seven *H. avivoca*, one *H. andersonii*, two *H. arenicolor*, and one *H. femoralis*) based on several different data sets. A total of 244 loci were used for phylogenetic analyses after removing sequences with evidence of paralogy in *H. chrysoscelis*. Phylogenetic analyses were conducted using RAxML under a GTR + G model (Lanave et al. 1984; Tavaré 1986; Lewis 2001) and for all analyses both gene and concatenated supermatrix trees were estimated. In the supermatrix analysis, the substitution model was partitioned by locus. We used the full data set along with various subsets of the data to fully characterize the evolutionary history of the system. These data sets included: 1) full data set, 2) *H. chrysoscelis* and outgroups only, 3) VERS MIN and outgroups only, 4) VERS MAX and outgroups only, 5) VERS MIN plus *H. chrysoscelis*, and 6) VERS MAX plus *H. chrysoscelis*.

### Population Genetic Analyses

To characterize the population structure of the gray treefrog complex, we conducted STRUCTURE (version 2.3.4) (Pritchard et al. 2000; Falush et al. 2003) analyses to understand the population structure of *H. versicolor* and *H. chrysoscelis*. We conducted analyses with *H. chrysoscelis, H. versicolor* and with a data set that included the complex sister species *H. avivoca.* We included all four SNP genotypes in *H. versicolor* and two SNP genotypes for each polymorphic site in diploids—coding the remaining two SNPs as missing data. Importantly, SNP identification is done prior to phasing and are not restricted to any subgenome. Each analysis was conducted both with 8,683 SNPs from 244 loci as well as one SNP per locus (244 total) with ten replications for each *K* value from 2 to 7. MCMC chains were run for 150k samples with a 50k sample burn-in period and verified for consistency across each replicate. Analyses were input into STRUCTURE HARVESTER (version 0.6.94) (Earl and vonHoldt 2012) and CLUMPP (version 1.1.2) (Evanno et al. 2005; Jakobsson and Rosenberg 2007) to summarize across all runs. Final STRUCTURE plots were created with distruct (version 1.1) (Rosenberg and Nordborg 2002). We did not determine the optimal *K* value based on any one method, instead investigating all *K* values analyzed, because results derived from suboptimal *K* values can be highly informative and choosing a single *K* value by any method can be misleading (Pritchard et al. 2000; Evanno et al. 2005; Meirmans 2015). In addition to STRUCTURE analyses, we also conducted a principle components analysis (PCA) as implemented in the R package “hierfstat” using the 8,683 SNPs previously generated. Finally, we estimated the distribution of polymorphisms across species and lineages as an assessment of incomplete lineage sorting (ILS). Specifically, we looked at the number of SNPs with fixed differences between *H. versicolor* and *H. chrysoscelis*, polymorphisms shared between the two species, and polymorphisms unique to each species. In addition to comparing polymorphisms across species, we also compared the number of polymorphisms for each category between *H. chrysoscelis* and each *H. versicolor* mitochondrial lineage individually (see Mitochondrial Phylogenetic Relationships and Coalescent Timing Results).

### Mitochondrial Phylogenetic Analysis and Coalescent Timing Estimation

To reconstruct the history of mitochondrial evolution in the gray treefrog complex, we used a published molecular clock estimate of a gene sequenced in this study to infer coalescent times and the potential dates of any whole-genome duplication events. We conducted analyses in BEAST 2.5.0 (Drummond and Rambaut 2007; Bouckaert et al. 2014) to estimate divergence times of *H. versicolor* progenitors from *H. chrysoscelis* and the approximate time of clade divergences within each species. We used a random local clock model with a coalescent constant population tree prior. Due to the paucity of available fossils for calibration at the scale of our study and the lack of genes with direct clock rate measurements, we generated a distribution for the molecular clock rate based on published results from related taxa of the *ND2* gene—defined with a gamma prior bounded from 0.005 to 0.015 with *α* = 0.001 and *β* = 5,000 to account for variability in the clock rate found across studies (Macey et al. 1998; Crawford 2003a; Barrow et al. 2017). We ran the BEAST analysis with a single partition under a GTR substitution model and a gamma distribution for among-site rate variation (Lanave et al. 1984; Tavaré 1986; Lewis 2001). Finally, we conducted all analyses with a random initial starting tree for 10 million MCMC generations with 1 million generations for burn in.

### Migration and Descendance Model Testing

To determine the identity and number of ancestors that gave rise to tetraploid *H. versicolor*, we used the program migrate-n (version 4.2.14) (Beerli 2006; Beerli and Palczewski 2010) to test if *H. versicolor* originated a single or multiple times and to identify who the progenitors of *H. versicolor* were. More specifically, we investigated the probability of descendance with and without migration of each *H. versicolor* mitochondrial lineage from each *H. chrysoscelis* nuclear genetic lineage (as determined by STRUCTURE) and the other *H. versicolor* mitochondrial lineages. Due to computational constraints, we subsampled 50 random loci from the five individuals from each lineage that best encompassed the entire geographic and genetic diversity of that lineage (bolded individuals in supplementary table 1, Supplementary Material online, lineage in parentheses). We restricted the analyses to the MIN alleles in order to directly test whether these putative *H. chrysoscelis* alleles (present in the tetraploid *H. versicolor*) were in fact directly descended from *H. chrysoscelis* or instead descended from another *H. versicolor* lineage. We recognize that using MIN alleles alone may presuppose the selection of models including migration with *H. chrysoscelis*, but several other aspects of our study assess gene flow with *H. chrysoscelis* and any conclusions are not drawn from this analysis alone.

For each analysis, we assumed an HKY mutation model with a uniform prior of mutation scaled population sizes (*θ*) between 0 and 0.05 for each population and a uniform migration rate prior distribution between 0 and 1,500. Prior distributions were chosen based on results from shorter preliminary analyses to ensure posterior distributions could be accurately estimated. We summarized each analysis across 50 replicates with four heated chains (chain temperatures: 1.0, 1.5, 3.0, 10^6^), and each replicate consisted of 4×10^7^ MCMC steps for each locus of which the first 1×10^7^ steps were discarded as burn-in. Finally, we used the Bezier approximation of log-marginal likelihoods calculated from each analysis to assess the probability of each model analyzed.

We conducted migrate-n analyses using two steps. The objective of the first step was to determine the number of *H. versicolor* origins, such that if *H. versicolor* had a single origin, we would expect to observe only one *H. versicolor* lineage with a high probability of descendance from any *H. chrysoscelis* lineage. Conversely, if *H. versicolor* originated multiple times, we would expect to observe multiple *H. versicolor* lineages with a high probability of descendance from *H. chrysoscelis* lineages. To conduct this analysis, we tested a set of divergence and migration models for each *H. versicolor* lineage individually, only altering which population was providing migrants into the chosen lineage for migration. To test descendance, we altered which *H. versicolor* or *H. chrysoscelis* population gave rise to the chosen lineage, and we allowed for migration if those two populations had any area of sympatry. The migration model was constrained to allow only migration between lineages in contact, with migration across ploidies restricted to diploid into tetraploid populations. We chose to allow for only unidirectional migration from diploid to tetraploids as polyploids are generally more tolerant of hybridization especially when involving differing ploidy levels and diploid to tetraploid gene flow is most commonly observed in nature (results from our ABC model testing and STRUCTURE analyses also support this limitation) (Stebbins 1947, 1971; Petit et al. 1999; Bogart and Bi 2013).

The objective of the second step was to test specific hypotheses about the evolutionary history of all *H. versicolor* lineages together. After running all analyses in the first step, we chose a final set of six different models to test different hypotheses of the history and formation of the complex. The final models chosen for this analysis were included based on the initial individual lineage analyses described above, but when two lineages had high support for descendance from the same population, we tested whether or not those lineages were independently formed from the source population or if they were formed through a stepping-stone migration pattern (fig. 4; models 1–5). Additionally, we also tested these models against an independent polyploid formation model as suggested by Holloway et al. (2006) and Ptacek et al. (1994) (fig. 4; model 6).

Although our estimate of the inheritance mode suggested the majority of loci are inherited tetrasomically (fig. 3*b*), previous work has demonstrated inheritance polymorphisms in *H. versicolor* (Danzmann and Bogart 1982, 1983). We restricted migrate-n analyses to MIN alleles to ensure we were, to the best of our ability, only testing whether a proportion of the *H. versicolor* genome originally descended from *H. chrysoscelis* or *H. versicolor* lineages and not whether the whole-genome descended from a single lineage due to the probable contributions of extinct lineages to *H. versicolor* (see Mitochondrial Phylogenetic Analysis and Coalescent Timing Estimation Results). Though we were unable to directly test whether *H. versicolor* MIN alleles descended from extinct or extant *H. chrysoscelis* lineages here, the present analysis should identify which extant *H. chrysoscelis* lineage most similar to the extinct lineage contributed, if any. Indeed, results from individual lineage analyses support this assertion (i.e., detecting NE *H. versicolor* descendance from EC *H. chrysoscelis*, table 2). A test of the possible contribution of extant and extinct *H. chrysoscelis* lineages to the original formation of *H. versicolor* is shown in the ABC and Polyploid Speciation Model Testing Methods and Results.

### ABC and Polyploid Speciation Model Testing

To identify the mode of polyploidization in the gray treefrogs, we used a modification of the framework outlined in Roux and Pannell (2015) in order to compare different models of polyploid evolution using ABC. In brief, to conduct this analysis we: 1) subselected loci and individuals and calculated summary statistic means and SDs to generate our observed data, 2) estimated a nuclear molecular clock rate to be used for sequence simulation, 3) simulated multilocus genetic data from biologically realistic prior parameter distributions for a selection of polyploid speciation models, 4) calculated summary statistic means and SDs for each simulation, 5) estimated model probability with ABC, and 6) estimated the posterior distributions of the parameters for the best supported models. The parameters used for this analysis are explained in greater detail in supplementary methods, Supplementary Material online, and the following sections (Generating Observed Data through Assessment of Model Selection Robustness) outline the details of this analysis.

### Generating Observed Data

We randomly selected 50 loci to use as the basis for our simulations and for calculating our observed summary statistics. Results from Roux and Pannell (2015) suggest that 20 loci are sufficient for distinguishing among polyploid speciation models, but their study only considers models without migration. We chose to use 50 loci to increase our power to correctly identify the best supported model when including more complex migration histories. We subsampled to 50 loci to accommodate computational limitations, and because randomization tests of the observed 50 locus estimate against a null of 1,000 randomly generated 50 loci estimates demonstrates no test statistic calculated for 50 loci is outside of the null expectation (supplementary table 2, Supplementary Material online).

Alignments were first filtered to remove any columns with missing data to match the output of simulated sequences and accurately calculate the summary statistics for the observed data (full list of summary statistics and values for the observed data, supplementary table 2, Supplementary Material online; but also see Use of the D3 Statistic for Polyploid Inference in supplementary methods, Supplementary Material online). We used alignments that included a random haplotype from each diploid *H. chrysoscelis* and the MIN and MAX alleles from *H. versicolor*. The ABC approach is best suited to using these data generated from our MIN/MAX separation, as the simulated evolutionary histories are conducted where the subgenomes are treated as their own populations, but the final summary statistics are calculated with the subgenomes treated as a single population (i.e., blind to any demarcation). Thus, our MIN and MAX assignment of observed haplotypes is most similar to the simulated data; however, because our summary statistic calculations do not assume any subgenomic assignment, this analysis is not biased toward any one model (from the model in fig. 5*a*, MIN and MAX correspond to subgenomes A and B, respectively).

### Molecular Clock Rate Estimation

In practice, simulating the data for our models requires knowledge of the mutation rate for the chosen loci. Because we do not have molecular clock-rate estimates for our targeted loci, we used the software BEAST (version 2.5.0) (Drummond and Rambaut 2007; Bouckaert et al. 2014) to estimate a clock rate distribution for AHE loci (Barrow et al. 2018; Heinicke et al. 2018) in this group. We randomly selected 20 loci to calculate this distribution. For each locus, we set a gamma prior (*α* = 8.0, *X* = 0.88, offset = 2.1) on the coalescence time for the entire set of ingroup and outgroup samples based on the 95% credibility interval distribution of the coalescence time of that group as determined by our mitochondrial coalescent timing analysis. We used a GTR site model with a gamma distribution, a strict clock, and a Yule tree prior for each analysis. Each analysis was run for 1×10^7^ MCMC chains with a 2×10^6^ burn-in period. We used the mean value of the molecular clock rate across the 20 loci (multiplied by 10^−6^ to convert from mutations per site per million years to mutations per site per year) for our simulations, but allowed each simulated locus clock rate to be randomly chosen from a normal distribution with the mean being the mean clock rate across all loci (estimated as 8.62×10^−10^ from our BEAST analysis, a rate similar to that found previously in frogs, e.g., Crawford 2003b) and with a SD of 1×10^−10^ to allow variability across loci.

### Generating Simulated Data

We conducted 10^6^ multilocus simulations for six chosen models of polyploid evolution (possible model parameters fig. 5*a*). Based upon results from our nuclear and mitochondrial phylogenetic analyses, we designed the simulated models to allow for either allopolyploidy (formation by hybridization of EC *H. chrysoscelis* and an extinct lineage) or autopolyploidy (whole-genome duplication of an extinct lineage that split off from EC *H. chrysoscelis*) as the speciation mode. Chromosomal inheritance was limited to tetrasomic (all four tetraploid copies segregating freely) based on observed and simulated allele frequencies (see Site Frequency Spectrum Analysis Methods; fig. 3*b*); migration was either nonexistent, asymmetric bidirectional to both tetraploid subgenomes, or unidirectional from the diploid to both subgenomes. Prior distributions for population size, *T*_split,_*T*_WGD_, and migration parameters were kept identical across all models. We used the NE *H. versicolor* mitochondrial lineage and the Eastern *H. chrysoscelis* nuclear genetic lineage to generate our observed data and the parameters for simulating each model based on results from our migrate-n analyses. We only included individuals whose genome contained only a small admixture portion from neighboring conspecific populations as determined from our STRUCTURE analysis. We chose this approach to ensure our observed data were most similar to the simulated data, which do not account for migration between additional outside populations.

To generate our priors, we used a modified version of the prior distribution script from Roux and Pannell (2015). To simulate sequences, we used the program msnsam (Hudson 2002; Ross-Ibarra et al. 2008). To calculate the summary statistics for each simulated data set, we used a modified version of mscalc (Ross-Ibarra et al. 2009; Roux et al. 2011, 2014; Roux and Pannell 2015). All scripts and complete instructions for their use are available in the figshare repository or can be downloaded from www.github.com/wbooker/GTF_Polyploid_ABC (last accessed November 11, 2021).

### Estimating Model Probability and Posterior Distributions

We used the R package “abc” to estimate the probability of each model given our observed data (Csilléry et al. 2012). We conducted each model probability estimation using a feed-forward neural network by nonlinear multivariate regression where the model itself is considered as an additional parameter to be inferred (scripts were modified from Leroy et al. [2017]). We selected 0.5% of the replicate simulations closest to the observed values of the summary statistics with a weighted Epanechnikov kernel. Computations were performed with 35 neural networks and ten hidden networks in the regression. We then estimated the posterior distribution of the parameters for the three models with the highest probabilities with a neural network by nonlinear multivariate regression. For each model estimated, we conducted a logit transformation of the parameters for the 1,000 replicate simulations closest to the observed data (1.0% of the total number of simulations), and the posterior distribution of the parameters was inferred using 35 trained and ten hidden neural networks.

### Assessment of Model Selection Robustness

To determine if the results from our ABC analysis provided more or less support for the chosen model, we assessed the robustness of ABC to accurately identify the correct model for a given model probability. To assess robustness, we simulated 1,000 pseudo-observed data sets under both autopolyploid and allopolyploid models with tetrasomic and one-way AB migration (the two overwhelmingly supported models) and conducted ABC analyses for each of the pseudo-observed data sets. Robustness was assessed as P(M1|M1)P(M1|M1)+P(M1|M2).

### Site Frequency Spectrum Analysis

To better ascertain the mode of polyploid formation and chromosomal inheritance patterns, we estimated the distribution of allele frequencies, or the site frequency spectrum, for each *H. versicolor* lineage and compared these distributions to distributions generated from simulated data. The site frequency spectrum can be informative when analyzing genomic data and has been widely used to estimate demography and evolutionary histories (Gutenkunst et al. 2009). Because of the lack of recombination between subgenomes in the case of disomic inheritance (tetraploid subgenomes remain distinct, with segregation only between distinct subgenomic copies) and to a lesser extent heterosomic inheritance (mixed inheritance, with segregation between all four copies of some chromosomes, but only subgenomic copies for other chromosomes), the site frequency spectrum of a disomic or heterosomic polyploid should have an overabundance of alleles with a 50% frequency in the population as compared with tetrasomic polyploids (Hollister et al. 2012; Arnold et al. 2015). However, although one might observe such a pattern in an isolated population, it is unclear how various migration histories between diploids and tetraploids might affect our ability to discern a polyploid’s chromosomal inheritance pattern.

We simulated sequences and recorded allele frequencies using the same scripts, input files, and prior values as our ABC analysis for 45 polyploid speciation models (All combinations of inheritance, speciation, and migration patterns; outlined in Polyploid Speciation Model Testing Parameters in supplementary methods, Supplementary Material online). As in the ABC analysis, we assigned individuals to a genomic population only if their genome contained the few alleles from neighboring conspecific populations as determined from our STRUCTURE analysis. We ran 50,000 50-locus simulations for each model, using the same prior distributions of the parameters as our ABC analysis.

## Supplementary Material

Supplementary data are available at *Molecular Biology and Evolution* online.

## Supplementary Material

msab316_Supplementary_Data
