# Supplementary material for: The Complex History of Genome Duplication and Hybridization in North American Gray Treefrogs
Source: Mol Biol Evol. 2021 Nov 13;39(2):msab316. doi: 10.1093/molbev/msab316 (PMC8826561; doi:10.1093/molbev/msab316)
Supplement: msab316_Supplementary_Data [file msab316_supplementary_data.zip › Complex_History_of_GTF_Supplement.pdf]

Supplementary Methods

Sampling

For molecular data, a total of 35 *Hyla versicolor* and 71 *H. chrysoscelis* were sampled, including nearly all samples from the Ptacek et al. (1994) and Holloway et al. (2006) studies (Supp. Table 1). We also included 7 *H. avivoca,* the closest known relative of the gray treefrogs which often hybridizes with the diploid *H. chrysoscelis*, to examine their genomic contribution to this complex (Gerhardt 1974). Finally, 1 *H. andersonii*, 2 *H. arenicolor*, and 1 *H. femoralis*—three closely related species previously assigned to the *H. versicolor* group, but whose exact relationships remain unclear (Blair 1962; Faivovich et al. 2005; Duellman et al. 2016)—were used as outgroup taxa (see Supplementary Methods for information on sequencing and bioinformatic protocols). Field collections were conducted in accordance with appropriate state collection permits and Animal Care and Use Committee Protocols. Treefrogs were collected by hand and either euthanized, dissected, and vouchered for deposition at the Florida Museum of Natural History or toe-clipped and released at their original locality. Tissues (heart, liver, leg muscle, and/or toe clips) were preserved in tissue buffer (20% DMSO, 0.25 M EDTA, salt-saturated) and stored at -80°F at Florida State University. Additional samples were obtained via museum loans from the University of Texas at Austin Biodiversity Center (formerly Texas Natural History Collections), and the Illinois Natural History Survey.

Anchored Hybrid Enrichment and Sequencing

Genomic DNA was extracted using an E.Z.N.A. Tissue DNA Kit (Omega Bio-Tek). After extraction, genomic DNA was sonicated to a fragment size of ~300-800 bp using a Covaris E220 Focused-ultrasonicator with Covaris microTUBES. Subsequently, library preparation and indexing were performed on a Beckman-Coulter Biomek FXp liquid-handling robot following a protocol modified from Meyer and Kircher (2010). A size-selection step was also employed after blunt-end repair using SPRI select beads

(Beckman-Coulter Inc.; 0.9x ratio of bead to sample volume). Indexed samples were then pooled at equal quantities (16-18 samples per pool), and then each pool was enriched using amphibian-specific probes (Agilent Technologies Custom SureSelect XT kit) described elsewhere (Barrow et al. 2018; Heinicke et al. 2018). Capture probes were designed based on 15x raw genomic data (Illumina 2500 paired-end 150bp) derived from *Rana sphenocephala*, *Pseudacris feriarum*, and *Pseudacris nigrita* mapped to 364 target loci in the *Xenopus tropicalis* genome (xenTro3). The 364 loci have been used broadly in vertebrate phylogenomic studies (e.g., Lemmon et al. 2012; Ruane et al. 2015; Prum et al. 2015; Barrow et al. 2018). After enrichment, 3-4 enrichment reactions were pooled in equal quantities for each sequencing lane and sequenced on two PE150 Illumina HiSeq 2500 lanes at the Translational Science Laboratory in the College of Medicine at Florida State University.

Anchored Hybrid Enrichment Bioinformatics

The raw sequencing reads were processed following methods previously described (Lemmon et al. 2012; Rokyta et al. 2012; Prum et al. 2015; Ruane et al. 2015; Hamilton et al. 2016). Reads were demultiplexed with no mismatches tolerated and pairs were merged as in Rokyta et al. (2012). Merged reads were assembled using a quasi-denovo assembly approach described in Prum (2015) and Hamilton et al. (2015) using reference sequences for *Xenopus*, *Rana*, and *Pseudacris*. Alleles were phased using assembled read overlap information in a Bayesian statistical framework and orthology was assessed using sequence similarity (details in Pyron et al. 2016).

Paired-read merging In order to increase read accuracy and length, paired reads were merged prior to assembly following Rokyta et al. (2012). In short, for each degree of overlap each read pair was evaluated with respect to the probability of obtaining the observed number of matches by chance. The overlap with the lowest probability was chosen if the p-value was less than 10^{-10}. This low p-value avoids chance matches in

repetitive regions. Read pairs with a p-value below the threshold were merged and quality scores were recomputed for overlapping bases (Rokyta et al. 2012). Read pairs failing to merge were utilized but left unmerged during the assembly.

Read assembly Divergent reference assembly was used to map reads to the probe regions and extend the assembly into the flanking regions (Prum et al. 2015). More specifically, a subset of the taxa used during probe design were chosen as references for the assembly: *Pseudacris nigrita* (Hylidae) and *Gastrophryne carolinensis* (Microhylidae). Matches were called if 17 bases matched a library of spaced 20-mers derived from the conserved reference regions (i.e., those used for probe design). Preliminary reads were then considered mapped if the 55 matches were found over 100 consecutive bases in the reference sequences (all possible gap-free alignments between the read and the reference were considered). The approximate alignment position of mapped reads were estimated using the position of the spaced 20-mer, and all 60-mers existing in the read were stored in a hash table used by the de-novo assembler. The de-novo assembler identifies exact matches between a read and one of the 60-mers found in the hash table. Simultaneously using the two levels of assembly described above, the three read files were traversed repeatedly until a pass through the reads produced no additional mapped reads. For each locus, a list of all 60-mers found in the mapped reads was compiled and 60-mers were clustered if found together in at least two reads. Contigs were estimated from 60-mer clusters. In the absence of contamination, low coverage, or gene duplication each locus should produce one assembly cluster. Consensus bases were called from assembly clusters as unambiguous base calls if polymorphisms could be explained as sequencing error (assuming a binomial probability model with the probability of error equal to 0.1 and alpha equal to 0.05). Otherwise ambiguous bases were called following IUPAC codes (e.g., 'R' was used if 'A' and 'G' were observed). Called bases were soft-masked (made lowercase) for sites with coverage lower than 5. Assembled contigs derived from less than 400 reads were removed to reduce the effects of cross contamination and rare sequencing

errors in index reads (Assembly code available at doi:10.5061/dryad.51v22).

Allele phasing Using the Bayesian approach developed by Pyron et al. (2016), we took the identified polymorphic sites within each assembly and used the read overlap information to determine the posterior distribution of allele phasing under assumptions of diploidy and separately under the assumption of tetraploidy. Markov chains were run for 20,000 generations, sampling every 100 generations after the first 10,000 generates were discarded as burnin. Haplotypes for each individual locus combination were identified by drawing a sample from the posterior distribution for the relevant assembly.

Orthology assessment After grouping homologous sequences obtained by enrichment and whole genomes, orthology was determined for each locus following (Prum et al. 2015; Hamilton et al. 2016). For each locus, pairwise distances among homologs were computed using an alignment-free approach based on percent overlap of continuous and spaced 20-mers. Using the distance matrix, sequences were clustered using a Neighbor-Joining algorithm, but allowing at most one sequence per species to be in a given cluster. Note that flanks recovered through extension assembly contain more variable regions and allow gene copies to be sorted efficiently. Gene duplication before the basal ancestor of the clade results in two distinct clusters that are easily separated. Duplication within the clade typically results in two clusters, one containing all of the taxa and a second containing a subset of the taxa (missing data). Gene loss also results in missing data. In order to reduce the effects of missing data, clusters containing fewer than 50% of the species were removed from downstream processing.

Alignment and trimming Orthologous sequences were processed using a combination of automated and manual steps in order to generate high quality alignments in a reasonable amount of time. Sequences in each orthologous cluster were aligned using MAFFT v7.023b (Katoh et al. 2002; Katoh and Standley 2013), with --genafpair and

--maxiterate 1000 flags utilized. The alignment for each locus was then trimmed/masked using the steps from Prum et al. (2015). First, each alignment site was identified as “conserved” if the most commonly observed character was present in >50% of the sequences. Second, using a 20bp window, each sequence was scanned for regions that did not contain at least 12 characters matching to the common base at the corresponding conserved site. Characters from regions not meeting this requirement were masked. Third, sites with fewer than 32 unmasked bases were removed from the alignment. A visual inspection of each masked alignment was carried out in Geneious version 7 . Regions of sequences identified as obviously misaligned or paralogous were removed. SNPs were extracted from alignments using a custom Java script that identified polymorphic sites flanked on either side by 5 base pairs that were conserved across all taxa.

PloiydyPal

PloidyPal takes advantage of phased allele data by taking as input 4 phased sequences at each locus for each individual, regardless of actual ploidy, and compares the pairwise genetic distances between the four “alleles”. For tetraploids, phasing under a four-allele assumption produces four different alleles reflecting their actual state. For diploids, however, phasing under a four-allele assumption tends to produce four alleles with a bimodal pairwise genetic distance distribution. In an ideal situation, all six of the pairwise genetic distances are non-zero for each tetraploid, whereas only four of the pairwise genetic distances are non-zero for each diploid.

After computing pairwise genetic distances, loci are evaluated as to how well alleles from these loci can be used to distinguish the control diploids from the control tetraploids. The loci identified as diagnostic are then used to determine the ploidy of the remaining individuals. Importantly, control individuals are only used to identify diagnostic loci; final ploidy determination is done without influence of the controls.

Mitochondrial Genome Assembly

Although we enriched for nuclear loci only, non-target mitochondrial (mtDNA) reads are normally very high in copy number relative to the nuclear genome and so were also sequenced as bycatch, allowing us to extract mtDNA genomes from the enriched sequence data. We used SeqMan NGEN v. 13.0.1 (DNASTAR, Inc., Madison, WI) to map mtDNA reads to reference sequences. To develop reference sequences, we started with closely related species using a minimum match percentage of 70%. We first assembled *H. chrysoscelis* and *H. andersonii* mtDNA genomes using a previously assembled *H. andersonii* mtDNA genome as a reference (Warwick 2016). The assembled *H. chrysoscelis* reference was then used to develop mtDNA genomes for *H. versicolor*, *H. avivoca, H. arenicolor,* and *H. femoralis.* After developing the reference sequence for each species, individuals were mapped to the appropriate reference using a minimum match percentage of 85%.

Polyploid Speciation Model Testing Parameters

The framework for simulating data for our ABC analysis allows for 45 total possible models given the same prior distributions of parameters. The 45 models are possible given three modes of polyploidization, three chromosomal inheritance patterns, and 5 different migration histories. The three polyploidization modes and their parameters that could be simulated under this framework are: Allopolyploidization (two ancestral lineages split at time T_{split}, combined to form a polyploid at time T_{WGD}), Autopolyploidization (Two lineages split at time T_{split}, one lineage splits into two subgenomes at time T_{WGD}; in effect, this is autopolyploidization of a sister lineage to the sampled diploid lineage), and Autopolyploid Speciation (lineages and subgenomes both split from a single ancestral population at time $T_{split} = T_{WGD}$; in effect, this is autpolyploidization of the sampled diploid lineage). Chromosomal inheritance is simulated by allowing either no migration across subgenomes (disomic inheritance), 10 migrants per generation between subgenomes

across all loci (tetrasomic inheritance), or 10 migrants per generation between subgenomes for a random number of loci (heterosomic inheritance). Ten migrants were chosen as this is sufficient to prevent differentiation between the subgenomes at the chosen locus (Templeton 2006; Roux and Pannell 2015). Finally, migration was simulated as occurring either asymmetrically between both diploids and tetraploids or in a single direction with only the tetraploid receiving migrants from the diploid. Migration patterns can also be divided subgenomically, with migration occurring either between the diploid and both subgenomes (migAB, Fig. 5) of the tetraploid or between the diploid and only one subgenome (migA, Fig. 5). The number of migrants in any one simulation was generated by sampling from a beta distribution, whose parameters themselves were drawn from a uniform distribution. Prior distributions for these parameters were: scalar (0,10), α (0,10), and β (0,100).

Use of the D3 Statistic for Polyploid Inference

In addition to summary statistics already reported in the literature, we also used a modified version of the D3 statistic (Hahn and Hibbins 2019) for our ABC analysis. The D3 statistic was developed as a metric to detect introgression using only three samples, comparing pairwise distances of two sister lineages to a relative of those two lineages. In brief, significant deviations from a D3 value of zero likely indicates introgression between one of the ingroup lineages and the sister of the two ingroup lineages.

For use in our ABC analysis, we calculated the D3 statistic with the two tetraploid (*H. versicolor*) alleles as our ingroup lineages and a random diploid (*H. chrysoscelis*) allele as the outgroup lineage. Calculating the D3 statistic this way would allow us to distinguish if one *H. versicolor* allele was more similar to *H. chrysoscelis*—potentially indicating an allopolyploid hybird origin of *H. versicolor.* Additionally, differing chromosomal inheritance patterns should also affect the D3 statsitic—as increasing tetrasomic inheritance should erode any differences between the two tetraploid alleles and push D3 to zero. Finally, we calculated only the absolute value of every D3 value at each locus because

we did not want to bias our results by having separated the MIN/MAX subgenomes (below referred to as D3a; 'a' referring to absolute value).

In addition to calculating this statistic to use in our ABC analysis, we were also interested in the behavior of this statistic under various heteroploidy migration histories. To evaluate the utility of this statistic, we conducted 1000 20-locus simulations using the same scripts, software, input files, and prior values as we did for the ABC model testing analysis. We reported the mean and standard deviation D3a value calculated for each 20 locus simulation, resulting in a final dataset of 1000 values for each of the models.

Supplementary Results

D3a Statistic

Simulations of the D3a statistic under each of the possible models show that this statistic can be useful in several ways, particularly in conjunction with other statistics to distinguish among polyploid speciation, inheritance, and migration models. Overall, when there is no migration between diploids and tetraploids, the average and standard deviation D3a values are both near zero except under allopolyploid disomic and heterosomic models (Supp. Fig. 13,14). Migration between diploids and tetraploids tends to produce higher average D3a values—with bidirectional migration models having the highest D3a values. Similarly, the standard deviation of D3a values across loci also distinguishes between migration models in the same fashion. Increasing levels of disomic inheritance tends to elevate average D3a values across all models as well as the standard deviation of D3a for models of no migration or bidirectional migration. While there is a large amount of variation within specific models, our results suggest that the D3a statistic can be generally useful for informing migration models, and if migration models are known, can be informative for determining chromosomal inheritance patterns and distinguishing allo vs. autopolyploidy in some cases.

References

Barrow, L. N., A. R. Lemmon, and E. M. Lemmon. 2018. Targeted sampling and target capture: Assessing phylogeographic concordance with genome-wide data. Systematic Biology 67:979–996.

Blair, W. F. 1962. Non-morphological data in anuran classification. Systematic zoology 11:72.

Duellman, W. E., A. B. Marion, and S. B. Hedges. 2016. Phylogenetics, classification, and biogeography of the treefrogs (*Amphibia*: *Anura*: Arboranae). Zootaxa 4104:1–109.

Faivovich, J., C. F. Haddad, P. C. Garcia, D. R. Frost, J. A. Campbell, and W. C. Wheeler. 2005. Systematic review of the frog family hylidae, with special reference to hylinae: phylogenetic analysis and taxonomic revision. Bulletin of the American Museum of Natural History 294:1.

Gerhardt, H. C. 1974. The vocalizations of some hybrid treefrogs: acoustic and behavioral analyses. Behaviour 49:130–151.

Hahn, M. W. and M. S. Hibbins. 2019. A three-sample test for introgression. Molecular biology and evolution 36:2878–2882.

Hamilton, C. A., A. R. Lemmon, E. M. Lemmon, and J. E. Bond. 2016. Expanding anchored hybrid enrichment to resolve both deep and shallow relationships within the spider tree of life. BMC Evolutionary Biology 16:212.

Heinicke, M. P., A. R. Lemmon, E. M. Lemmon, K. McGrath, and S. B. Hedges. 2018. Phylogenomic support for evolutionary relationships of new world direct-developing frogs (*Anura*: *Terraranae*). Molecular Phylogenetics and Evolution 118:145–155.

Holloway, A. K., D. C. Cannatella, H. C. Gerhardt, and D. M. Hillis. 2006. Polyploids with different origins and ancestors form a single sexual polyploid species. The American Naturalist 167:E88–101.

Katoh, K., K. Misawa, K.-i. Kuma, and T. Miyata. 2002. MAFFT: a novel method for rapid multiple sequence alignment based on fast fourier transform. Nucleic Acids Research 30:3059–3066.

Katoh, K. and D. M. Standley. 2013. MAFFT multiple sequence alignment software version 7: improvements in performance and usability. Molecular Biology and Evolution 30:772–780.

Lemmon, A. R., S. A. Emme, and E. M. Lemmon. 2012. Anchored hybrid enrichment for massively high-throughput phylogenomics. Systematic Biology 61:727–744.

Meyer, M. and M. Kircher. 2010. Illumina sequencing library preparation for highly multiplexed target capture and sequencing. Cold Spring Harbor Protocols 2010:pdb.prot5448.

Prum, R. O., J. S. Berv, A. Dornburg, D. J. Field, J. P. Townsend, E. M. Lemmon, and A. R. Lemmon. 2015. A comprehensive phylogeny of birds (*Aves*) using targeted next-generation DNA sequencing. Nature 526:569–573.

Ptacek, M. B., H. C. Gerhardt, and R. D. Sage. 1994. Speciation by polyploidy in treefrogs: Multiple origins of the tetraploid, *Hyla versicolor*. Evolution 48:898.

Pyron, R. A., F. W. Hsieh, A. R. Lemmon, E. M. Lemmon, and C. R. Hendry. 2016. Integrating phylogenomic and morphological data to assess candidate species-delimitation models in brown and red-bellied snakes (*Storeria*). Zoological journal of the Linnean Society 177:937–949.

Rokyta, D. R., A. R. Lemmon, M. J. Margres, and K. Aronow. 2012. The venom-gland transcriptome of the eastern diamondback rattlesnake (*Crotalus adamanteus*). BMC Genomics 13:312.

Roux, C. and J. R. Pannell. 2015. Inferring the mode of origin of polyploid species from next-generation sequence data. Molecular Ecology 24:1047–1059.

Ruane, S., C. J. Raxworthy, A. R. Lemmon, E. M. Lemmon, and F. T. Burbrink. 2015. Comparing species tree estimation with large anchored phylogenomic and small sanger-sequenced molecular datasets: an empirical study on malagasy pseudoxyrhophiine snakes. BMC Evolutionary Biology 15:221.

Templeton, A. R. 2006. Population Genetics and Microevolutionary Theory. John Wiley & Sons, Inc., Hoboken, NJ, USA.

Warwick, A. 2016. Ecology, Evolution, and Conservation of the Pine Barrens Treefrog (*Hyla andersonii*). Retrieved from http://purl.flvc.org/fsu/fd/FSU_2016SU_Warwick_fsu_0071E_13389. Doctoral thesis Florida State University.

Supplementary Tables and Figures

Supplemental Table 1. Locality and specimen details for samples used in this study. Bold specimens indicate samples used in our migrate-n analysis.

Species	Map ID	Collab ID	State	County	Latitude	Longitude
Hyla andersonii	NA	WED54451	NJ	Burlington	39.8559	-74.6869
Hyla arenicolor	NA	DCC3043	AZ	Coconino	35.0596	-111.719
Hyla arenicolor	NA	TJL 1286	TX	Presidio	29.9895	-104.104
Hyla avivoca	NA	MP710	AL	Macon	32.4253	-85.7476
Hyla avivoca	NA	DCC3857	GA	Chatham	31.9994	-81.1196
Hyla avivoca	NA	HCG21	LA	Grant	31.6944	-92.5396
Hyla avivoca	NA	H146	LA	Jefferson	29.8237	-90.1402
Hyla avivoca	NA	HCG84	LA	Madison	32.4437	-91.2876
Hyla avivoca	NA	MP607	MS	Hinds	32.2889	-90.8041
Hyla avivoca	NA	HCG28	TN	Obion	36.3469	-89.1706
Hyla chrysoscelis	36	MP816	VA	Mecklenberg	36.5929	-78.3428
Hyla chrysoscelis	37	MP 584	VA	Charles City	37.36	-77.06
Hyla chrysoscelis	**38**	**VA11**	**VA**	**Halifax**	**36.717**	**-78.8042**
Hyla chrysoscelis	**39**	**MP002**	**GA**	**Chatham**	**32.0288**	**-81.2694**
Hyla chrysoscelis	40	MP 674	VA	Charles City	37.36	-77.06
Hyla chrysoscelis	**41**	**MY01**	**MD**	**Harford**	**39.512**	**-76.4358**
Hyla chrysoscelis	42	ECM7850	FL	Leon	30.4914	-84.1134
Hyla chrysoscelis	43	MP205	SC	Jasper	32.47	-81.02
Hyla chrysoscelis	44	MP273	VA	Goochland	37.67	-77.84
Hyla chrysoscelis	45	NC11	NC	Orange	35.9536	-79.063
Hyla chrysoscelis	**46**	**FL08**	**FL**	**Alachua**	**29.6556**	**-82.3272**
Hyla chrysoscelis	47	MP649	GA	Houston	32.24	-83.37
Hyla chrysoscelis	48	MP732	FL	Leon	30.479	-84.235
Hyla chrysoscelis	49	FL06	FL	Alachua	29.6728	-82.3469
Hyla chrysoscelis	50	FL72	FL	Leon	30.4379	-84.2457

Supplemental Table 1. Locality and specimen details for samples used in this study. Bold specimens indicate samples used in our migrate-n analysis.

Species	Map ID	Collab ID	State	County	Latitude	Longitude
Hyla chrysoscelis	**51**	**ECM4690**	**FL**	**Liberty**	**30.2409**	**-85.0151**
Hyla chrysoscelis	52	ECM4533	FL	Leon	30.3249	-84.4583
Hyla chrysoscelis	**53**	**MP458**	**AL**	**Russell**	**32.22**	**-85.15**
Hyla chrysoscelis	54	MP139	LA	East Baton Rouge	30.3438	-91.0865
Hyla chrysoscelis	55	MP138	LA	East Baton Rouge	30.3438	-91.0865
Hyla chrysoscelis	**56**	**MP647**	**TX**	**Harrison**	**32.53**	**-94.35**
Hyla chrysoscelis	57	MP753	KY	Carlisle	36.8128	-89.0082
Hyla chrysoscelis	58	MP859	GA	Upson	32.9559	-84.4616
Hyla chrysoscelis	59	14-1	KY	Bell	36.7281	-83.7409
Hyla chrysoscelis	60	MP692	TN	Monroe	35.5233	-84.3628
Hyla chrysoscelis	61	MP670	FL	Okaloosa	30.7529	-86.6341
Hyla chrysoscelis	62	MP263	LA	St. Martins	30.2211	-91.958
Hyla chrysoscelis	63	MP847	LA	Lincoln	32.5345	-92.6899
Hyla chrysoscelis	64	MP195	LA	Allen	30.67	-92.79
Hyla chrysoscelis	65	MP696	TN	Shelby	35.29	-89.908
Hyla chrysoscelis	66	MP249	MS	Hinds	32.27	-90.42
Hyla chrysoscelis	**67**	**MP651**	**WV**	**Summers**	**37.5282**	**-80.9949**
Hyla chrysoscelis	68	MSC02	MO	Cape Giradeau	37.2403	-89.4988
Hyla chrysoscelis	69	ECM3053	IL	Jackson	37.7592	-89.2706
Hyla chrysoscelis	70	MP701	VA	Smyth	32.9476	-84.4568
Hyla chrysoscelis	71	MP 453	KY	Meade	37.93	-86.05
Hyla chrysoscelis	72	MP693	LA	St. Martins	30.2211	-91.958
Hyla chrysoscelis	73	DCC3751	IN	Monroe	39.187	-86.5
Hyla chrysoscelis	74	ECM4466	KY	Graves	36.8393	-88.5264
Hyla chrysoscelis	**75**	**DCC3883**	**AL**	**Bibb**	**32.9563**	**-87.1423**
Hyla chrysoscelis	76	INHS131T	IL	Edgar	39.7734	-87.6847
Hyla chrysoscelis	77	DCC3829	OH	Ross	39.395	-82.9432

Supplemental Table 1. Locality and specimen details for samples used in this study. Bold specimens indicate samples used in our migrate-n analysis.

Species	Map ID	Collab ID	State	County	Latitude	Longitude
Hyla chrysoscelis	78	MP630	KY	Laurel	37.13	-84.1
Hyla chrysoscelis	79	MP 454	KY	Meade	37.93	-86.05
Hyla chrysoscelis	80	MP452	KY	Meade	37.93	-86.05
Hyla chrysoscelis	81	ECM4330	IL	Jefferson	38.15	-88.847
Hyla chrysoscelis	82	MP724	MS	Hancock	30.2625	-89.5424
Hyla chrysoscelis	**83**	**MP350**	**MO**	**Phelps**	**37.7**	**-91.89**
Hyla chrysoscelis	84	MP386	MO	Howell	36.77	-91.87
Hyla chrysoscelis	85	P112	MO	Phelps	37.6184	-91.9983
Hyla chrysoscelis	86	14-6	MO	Phelps	37.6187	-92.0004
Hyla chrysoscelis	87	MP370	MO	Oregon	36.69	-91.41
Hyla chrysoscelis	**88**	**14-7**	**MO**	**Phelps**	**37.6187**	**-92.0004**
Hyla chrysoscelis	89	DCC3874	IA	Butler	42.7524	-92.8562
Hyla chrysoscelis	**90**	**MP706**	**IA**	**Clarke**	**41.029**	**-93.4506**
Hyla chrysoscelis	**91**	**MP773**	**MO**	**Barry**	**36.8838**	**-93.6648**
Hyla chrysoscelis	92	MP717	TX	Smith	32.6334	-95.3569
Hyla chrysoscelis	93	MP804	NE	Otoe	40.7272	-96.1212
Hyla chrysoscelis	94	MP723	TX	Bastrop	30.0797	-97.238
Hyla chrysoscelis	95	T-1	KS	Douglas	38.9181	-95.2311
Hyla chrysoscelis	96	INHS924T	IL	Ogle	42.0431	-89.3841
Hyla chrysoscelis	97	MP327	OK	Payne	36.1	-96.99
Hyla chrysoscelis	98	MP632	MN	Hennepin	45.0293	-93.5148
Hyla chrysoscelis	**99**	**MP802**	**MN**	**Ottertail**	**46.4265**	**-95.5577**
Hyla chrysoscelis	100	14-5	MI	Schoolcraft	46.2895	-85.9476
Hyla chrysoscelis	101	MP135	TX	Travis	30.32	-97.69
Hyla chrysoscelis	102	MP329	OK	Ottawa	36.85	-94.8
Hyla chrysoscelis	103	MP686	TX	Eastland	32.3354	-98.8294
Hyla chrysoscelis	104	C10	MN	Carver	44.8789	-93.7187

Supplemental Table 1. Locality and specimen details for samples used in this study. Bold specimens indicate samples used in our migrate-n analysis.

Species	Map ID	Collab ID	State	County	Latitude	Longitude
Hyla chrysoscelis	**105**	**TX25**	**TX**	**Kendall**	**29.9794**	**-98.7231**
Hyla femoralis		DCC3858	GA	Chatham	32.0054	-81.122
Hyla versicolor	1	MP702	MN	Clearwater	47.25	-95.25
Hyla versicolor	**2**	**MP795**	**MN**	**Ottertail**	**46.4265**	**-95.5577**
Hyla versicolor	3	DCC3864	MN	St. Louis	47.7681	-92.3931
Hyla versicolor	**4**	**MP099**	**OK**	**Cleveland**	**35.1595**	**-97.3985**
Hyla versicolor	5	MP045	MO	Greene	37.1868	-93.1264
Hyla versicolor	**6**	**INHS399T**	**IL**	**Iroquois**	**40.8791**	**-87.6876**
Hyla versicolor	7	MP759	MO	Barry	36.8838	-93.6648
Hyla versicolor	8	MP793	OK	Ottawa	36.8012	-94.71
Hyla versicolor	9	MP625	Ontario	Guelph	43.5497	-80.1784
Hyla versicolor	10	INHS950T	IL	Hancock	40.5864	-91.3065
Hyla versicolor	**11**	**DCC3832**	**PA**	**Crawford**	**41.5667**	**-80.45**
Hyla versicolor	**12**	**DCC3828**	**OH**	**Ross**	**39.395**	**-82.9432**
Hyla versicolor	13	DCC3768	IN	Porter	41.5167	-87.0833
Hyla versicolor	**14**	**MP019**	**TX**	**Bastrop**	**30.13**	**-97.41**
Hyla versicolor	15	MP359	MO	Ozark	36.7601	-92.1537
Hyla versicolor	16	MP524	MO	Phelps	37.7	-91.89
Hyla versicolor	**17**	**BCHV02**	**MO**	**Boone**	**38.7611**	**-92.1995**
Hyla versicolor	**18**	**MP409**	**MO**	**Howell**	**36.77**	**-91.87**
Hyla versicolor	**19**	**TXHV03**	**TX**	**Wood**	**32.5887**	**-95.3249**
Hyla versicolor	20	MP 825	KY	Meade	37.93	-86.05
Hyla versicolor	**21**	**MP700**	**TN**	**Shelby**	**35.29**	**-89.908**
Hyla versicolor	22	MP162	LA	Allen	30.67	-92.79
Hyla versicolor	23	MP678	WV	Summers	37.56	-80.92
Hyla versicolor	**24**	**DCC3787**	**NY**	**Rensselaer**	**42.55**	**-73.625**
Hyla versicolor	25	MP020	VA	Giles	37.3591	-80.5353

Supplemental Table 1. Locality and specimen details for samples used in this study. Bold specimens indicate samples used in our migrate-n analysis.

Species	Map ID	Collab ID	State	County	Latitude	Longitude
Hyla versicolor	26	DCC3807	CT	Tolland	41.8075	-72.2621
Hyla versicolor	**27**	**MP576**	**ME**	**Penobscot**	**44.9024**	**-68.6598**
Hyla versicolor	28	DCC3795	NY	Westchester	41.2728	-73.8942
Hyla versicolor	29	DCC3800	CT	Windham	41.7903	-71.9422
Hyla versicolor	**30**	**DCC3817**	**MD**	**Prince Georges**	**39.0572**	**-76.816**
Hyla versicolor	**31**	**DCC3816**	**MD**	**Prince Georges**	**39.0572**	**-76.816**
Hyla versicolor	**32**	**MP296**	**VA**	**Goochland**	**37.67**	**-77.84**
Hyla versicolor	33	MYHV05	MD	Harford	39.512	-76.4358
Hyla versicolor	34	DCC3823	MD	Ann Arundel	39.0572	-76.816
Hyla versicolor	35	MP809	VA	Mecklenberg	36.5929	-78.3428

Supplemental Table 2. Summary statistics used for the ABC analysis and the observed statistics estimated from 50 loci comparing Eastern *H. chrysoscelis* (A) Northeast*H. versicolor* (B). *p*-values were generated from randomization tests of the observed estimate for 50 loci against a null of 1000 50 random loci estimates.

**Statistic**	**Estimate (50 loci)**	***p*-value**
bialsites_avg	20.4600	0.694
bialsites_std	13.1411	0.564
sf_avg	0.00005	0.507
sf_std	0.00031	0.525
sxA_avg	0.00119	0.645
sxA_std	0.00125	0.590
sxB_avg	0.00986	0.432
sxB_std	0.00883	0.258
ss_avg	0.00309	0.693
ss_std	0.00262	0.627
successive_ss_avg	1.10000	0.499
successive_ss_std	1.44568	0.216
piA_avg	0.00152	0.606
piA_std	0.00124	0.302
piB_avg	0.00300	0.600
piB_std	0.00294	0.297
pearson_r_pi	0.55675	0.583
thetaA_avg	0.00158	0.737
thetaA_std	0.00117	0.562
thetaB_avg	0.00369	0.501
thetaB_std	0.00289	0.219
pearson_r_theta	0.52557	0.576
DtajA_avg	-0.21521	0.178
DtajA_std	0.72769	0.738
DtajB_avg	-0.83063	0.875
DtajB_std	0.71572	0.402
divAB_avg	0.00320	0.267
divAB_std	0.00436	0.116
netdivAB_avg	0.00094	0.138
netdivAB_std	0.00287	0.122
minDivAB_avg	0.00039	0.188
minDivAB_std	0.00227	0.228
maxDivAB_avg	0.00709	0.438
maxDivAB_std	0.00629	0.146
Gmin_avg	0.02343	0.316
Gmin_std	0.09375	0.429
Gmax_avg	2.93673	0.614
Gmax_std	1.37469	0.230
FST_avg	0.14819	0.458
FST_std	0.14471	0.061
D3a_avg	0.37378	0.851
D3a_std	0.11024	0.496
pearson_r_divAB_netDivAB	0.94684	0.079
pearson_r_divAB_FST	0.55624	0.333
pearson_r_netDivAB_FST	0.63705	0.572
ss_sf	0.02000	0.483
ss_noSf	0.86000	0.921
noSs_sf	0.02000	0.382
noSs_noSf	0.10000	0.244

Supplemental Table 3. Estimates of the average and standard deviation for Tajima's θ (π), Watterson's θ, and Tajima's D across all loci in *H. chrysoscelis*, all *H. versicolor* with MIN/MAX sequences combined or separated, and in individual *H. versicolor* lineages with MIN/MAX sequences combined or separated.

	H. chrysoscelis	All_MIN_MAX	All_MIN	All_MAX	NE_MAX	NE_MIN	MW_MIN	MW_MIN	SW_MAX	SW_MIN
pi_avg	0.00214	0.00293	0.00193	0.00373	0.00359	0.00198	0.00357	0.00204	0.00353	0.00187
pi_std	0.00133	0.00261	0.00218	0.00285	0.00271	0.00223	0.00292	0.00225	0.00288	0.00235
theta_avg	0.00321	0.00476	0.0027	0.00491	0.00383	0.00222	0.00379	0.00219	0.00389	0.00209
theta_std	0.00160	0.00312	0.00256	0.00301	0.00266	0.00244	0.00295	0.00237	0.00292	0.00233
Dtaj_avg	-1.08906	-1.32586	-0.97056	-0.93785	-0.33412	-0.46295	-0.32657	-0.31600	-0.52141	-0.56075
Dtaj_std	0.60000	0.61249	0.69021	0.59352	0.60692	0.74537	0.68073	0.76209	0.62047	0.69027

Supplemental Table 4. Coalescent timing for clades of interest estimated from BEAST analysis. Colors correspond to clade and background colors in Fig. 2

Clade	Fig. 2 Color	Mean	95% CI
All *H. chrysoscelis*/*H. versicolor*/*H. avivoca*	NA	1.80 Ma	0.989-2.85 Ma
All *H. chrysoscelis*	NA	1.26 Ma	0.675-1.99 Ma
NE *H. versicolor*/East *H. avivoca*	Green	1.51 Ma	0.812-2.37 Ma
NE *H. versicolor*	Green	0.262 Ma	0.125-0.426 Ma
MW *H. versicolor*	Yellow	0.338 Ma	0.131-0.430 Ma
Western *H. chrysoscelis*	Orange	0.262 Ma	0.130-0.430 Ma
FL Eastern/Eastern/Central *H. chrysoscelis*	Purple	0.811 Ma	0.431-1.27 Ma
Eastern/Central *H. chrysoscelis*	Purple	0.527 Ma	0.271-0.826 Ma
West *H. avivoca*, SW *H. versicolor*, Central *H. chrysoscelis* (CSW)	Light Blue	0.591 Ma	0.326-0.939 Ma
SW *H. versicolor*, Central *H. chrysoscelis* (CSW)	Light Blue	0.223 Ma	0.012-0.360 Ma

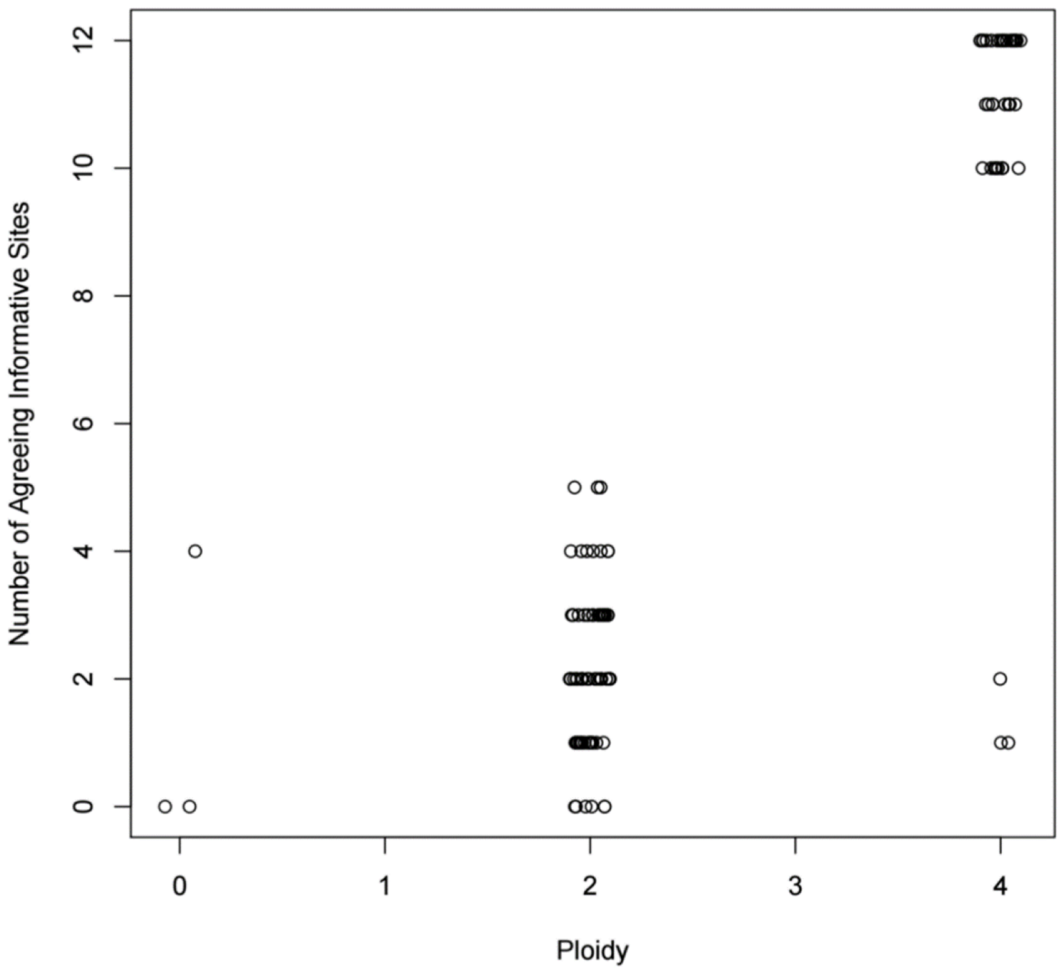

Supplemental Figure 1. Results from PloidyPal showing assessment of ploidy for all specimens. Samples with Ploidy of 0 were unidentified. The three samples with a low number of agreeing informative sites (loci) were misidentified and either corrected based on a reinvestigation of field notes or removed from the dataset.

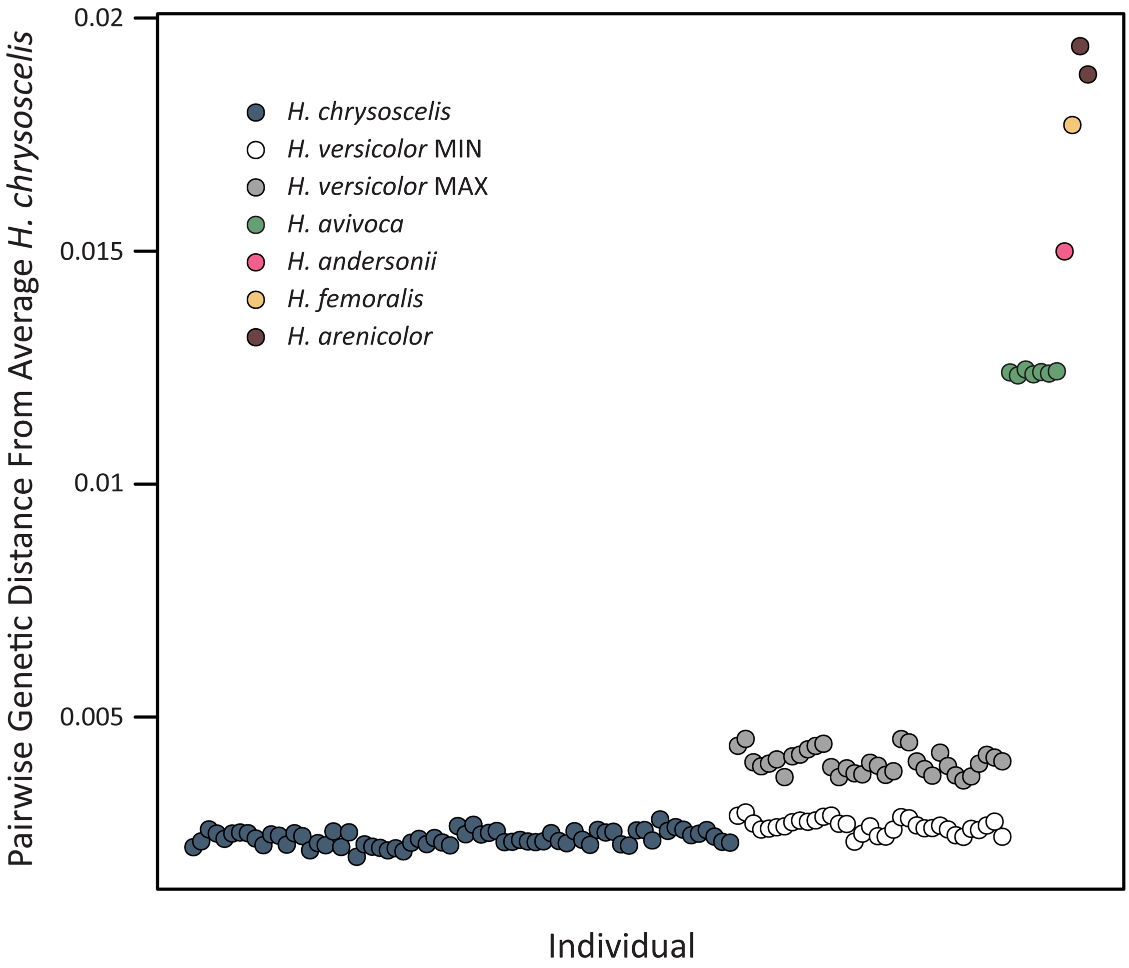

Supplemental Figure 2. Pairwise genetic distance for each sampled individual to the average *H. chrysoscelis*. Averages were generated by calculating the pairwise genetic distance for each individual to each *H. chrysoscelis* (excluding themselves if the sample was *H. chrysoscelis*) and averaging over the total. *H. versicolor* individuals are separated into putative MIN/MAX subgenomes on the same x-axis plane.

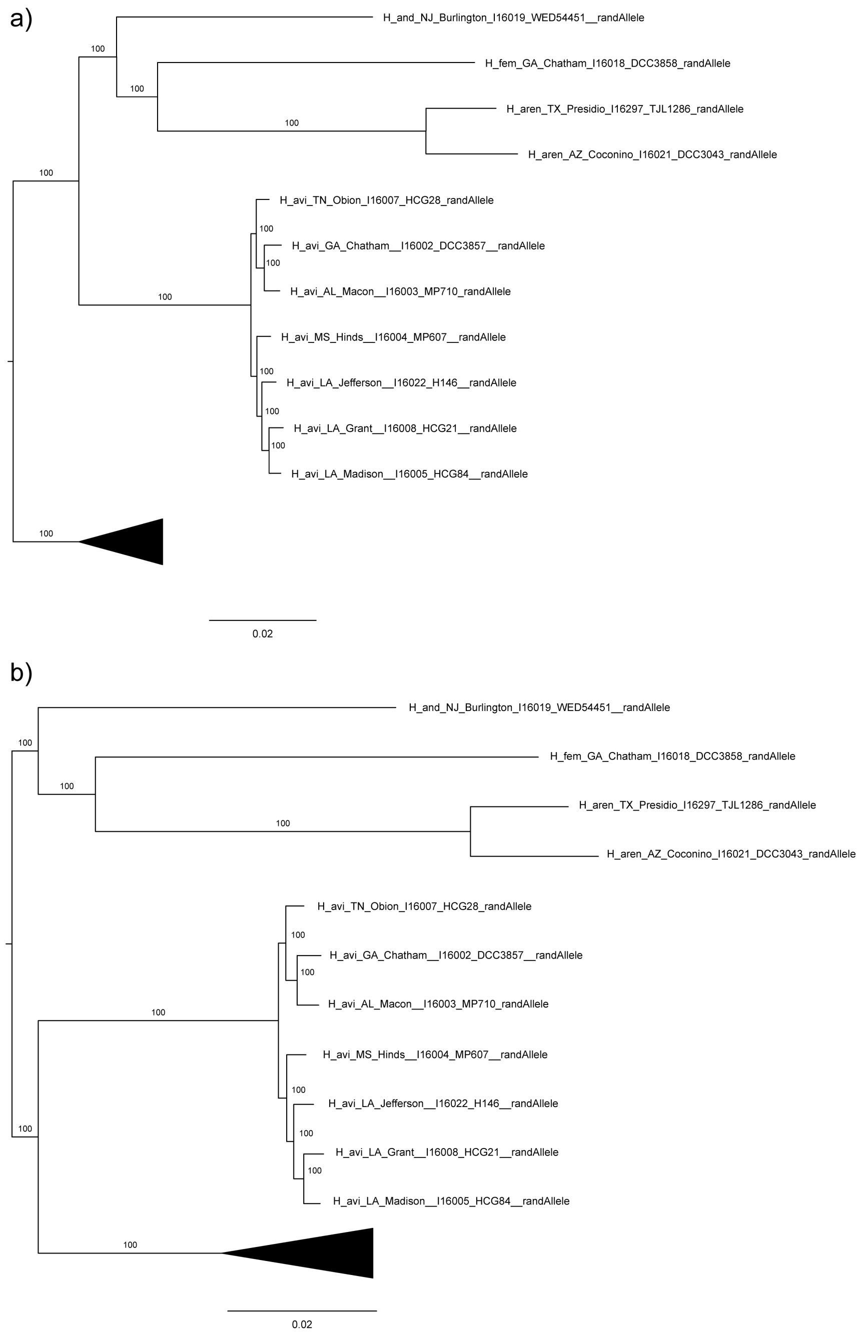

Supplemental Figure 3. Nuclear phylogenetic relationships of outgroup taxa (*H. andersonii*, *H. arenicolor*, and *H. femoralis*) and *H. avivoca* relative to *H. versicolor* and *H. chrysoscelis* (collapsed) with (a) mid-point rooting or (b) alternative rooting on non-*H. avivoca* outgroup branches from the RAxML concatenated analysis using 244 AHE loci (Dataset 1 shown; all datasets produced the same relationships and support). Branch labels show bootstrap support values. Scale bar and branch lengths represent substitutions per site.

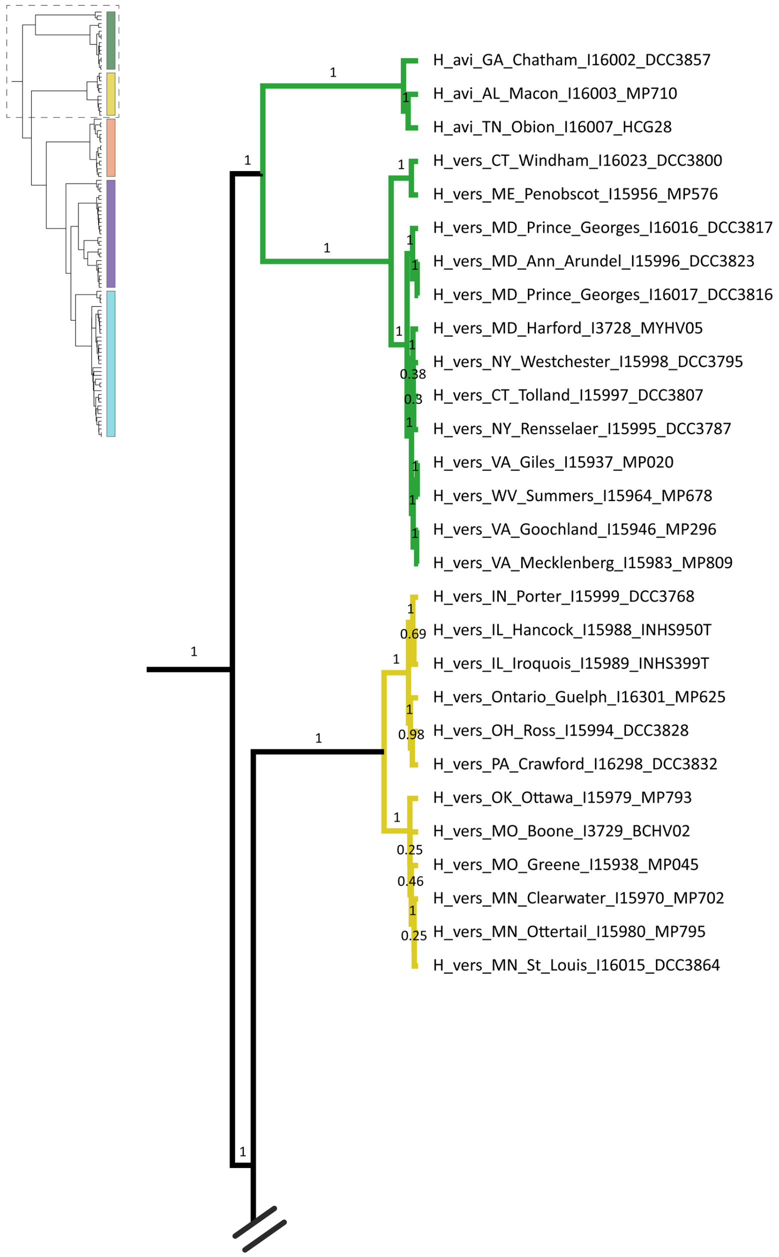

Supplemental Figure 4. Expanded mitochondrial tree shown in Fig. 2a from the BEAST 2 analysis. Branch labels show posterior probability.

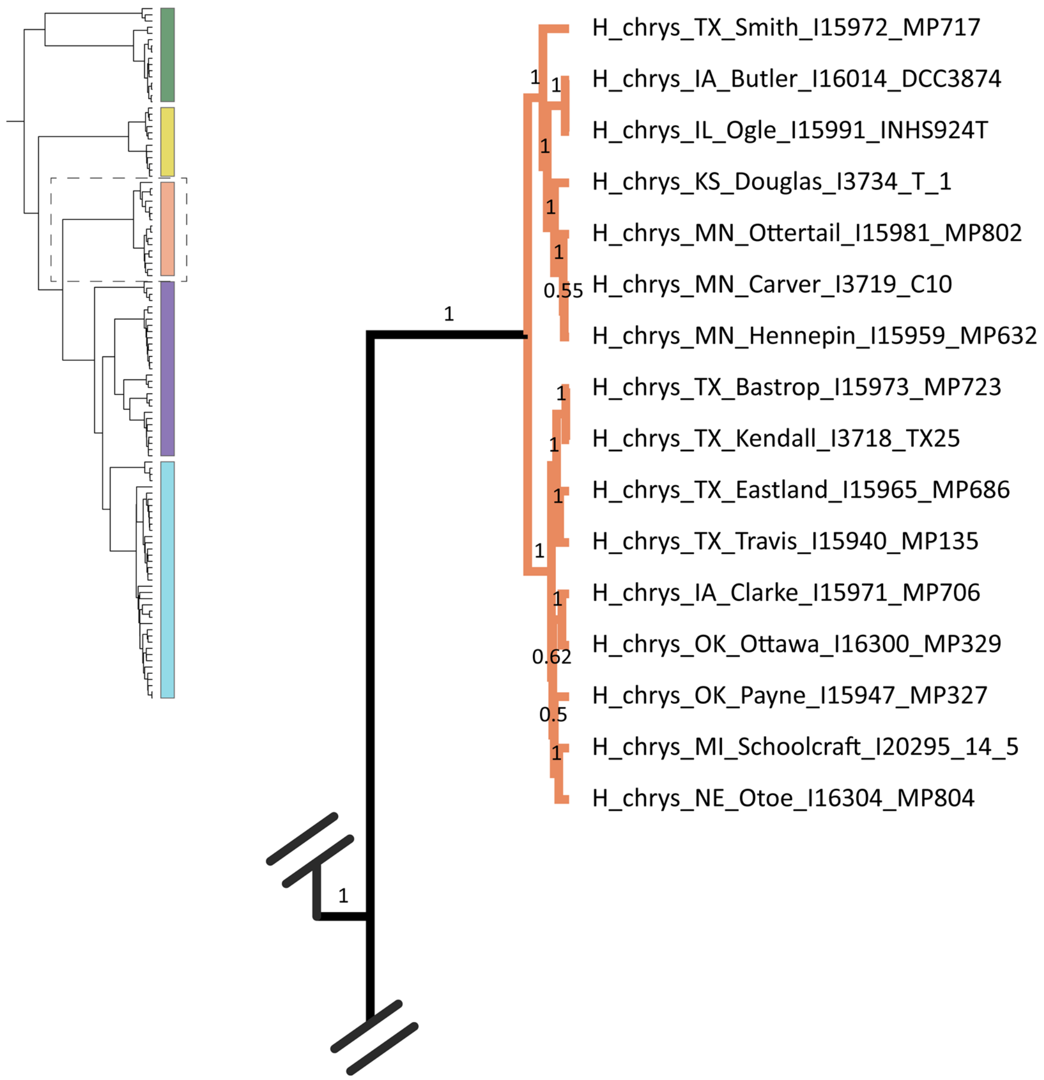

Supplemental Figure 4. Expanded mitochondrial tree shown in Fig. 2a from the BEAST 2 analysis. Branch labels show posterior probability.

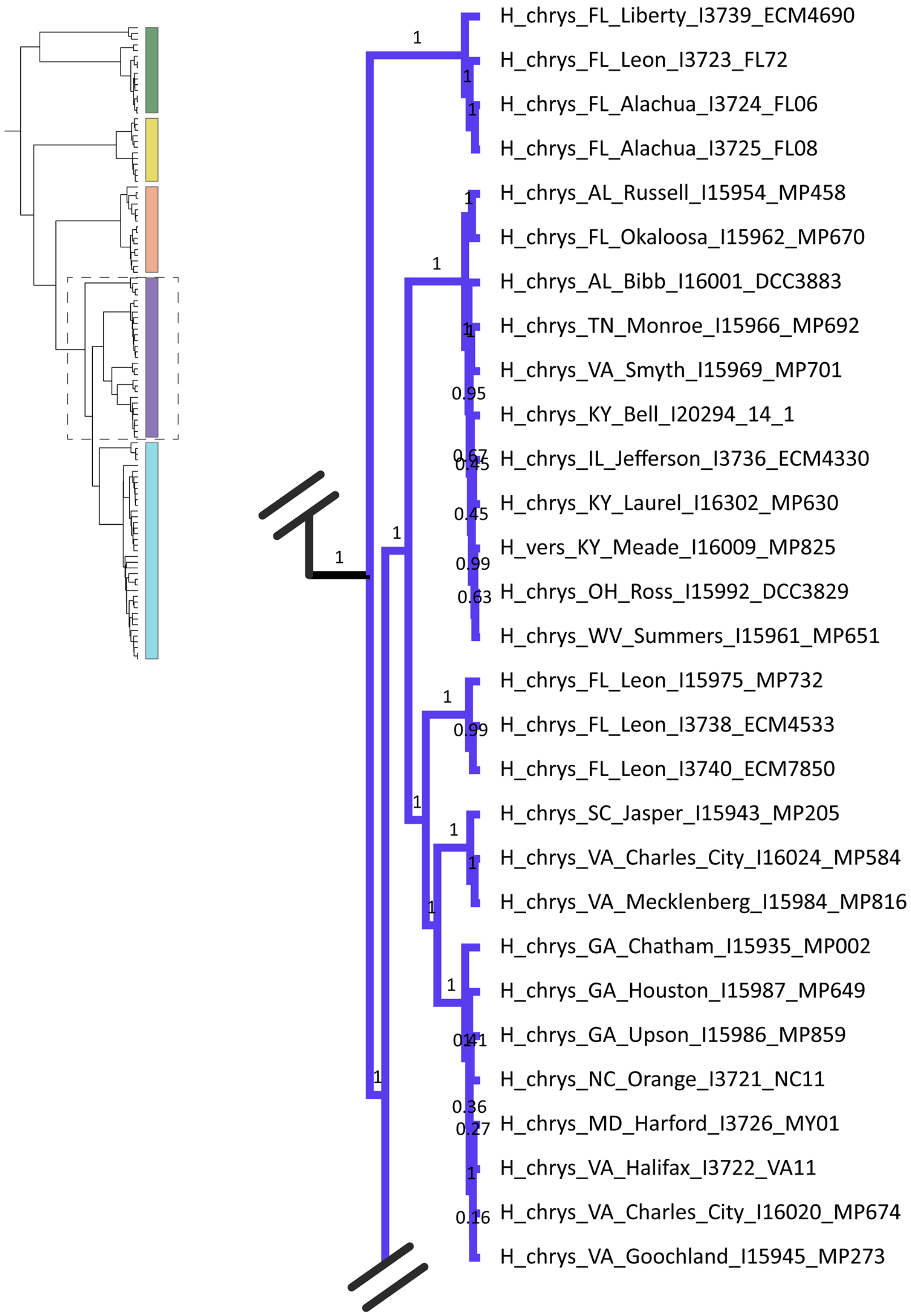

Supplemental Figure 4. Expanded mitochondrial tree shown in Fig. 2a from the BEAST 2 analysis. Branch labels show posterior probability.

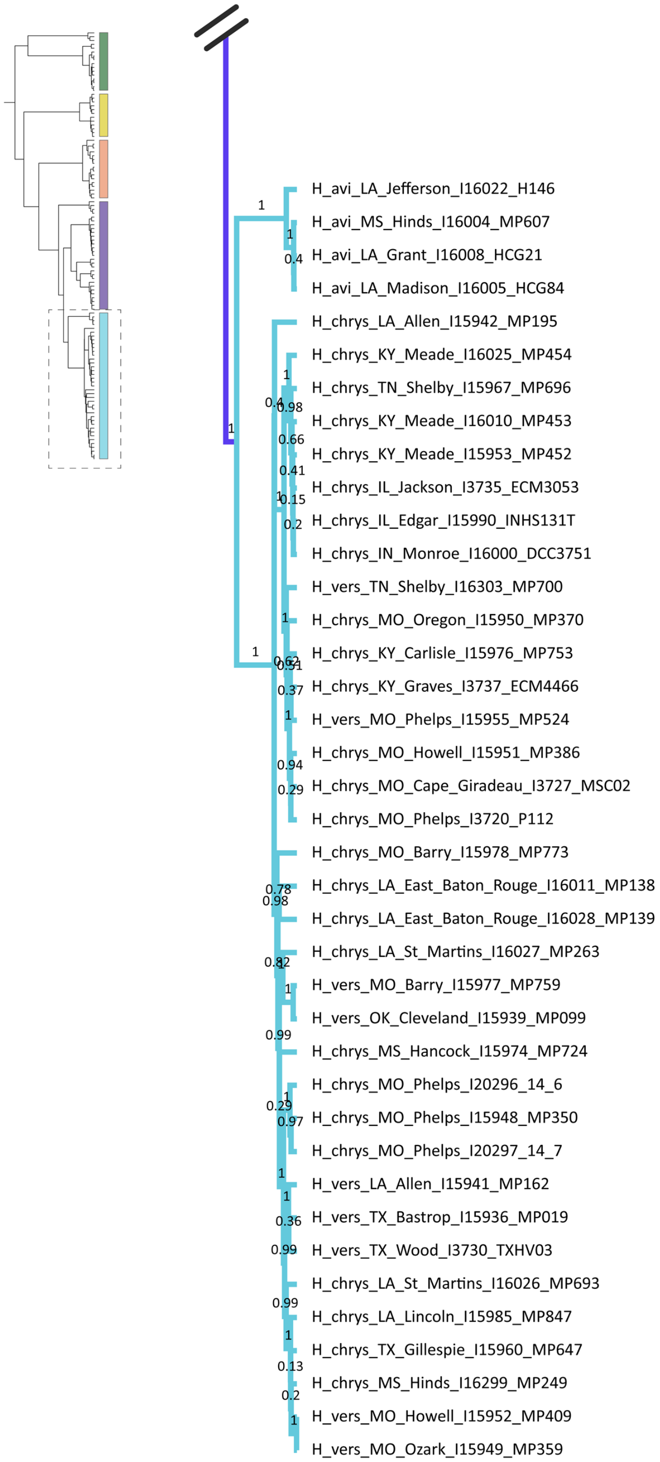

Supplemental Figure 4. Expanded mitochondrial tree shown in Fig. 2a from the BEAST 2 analysis. Branch labels show posterior probability.

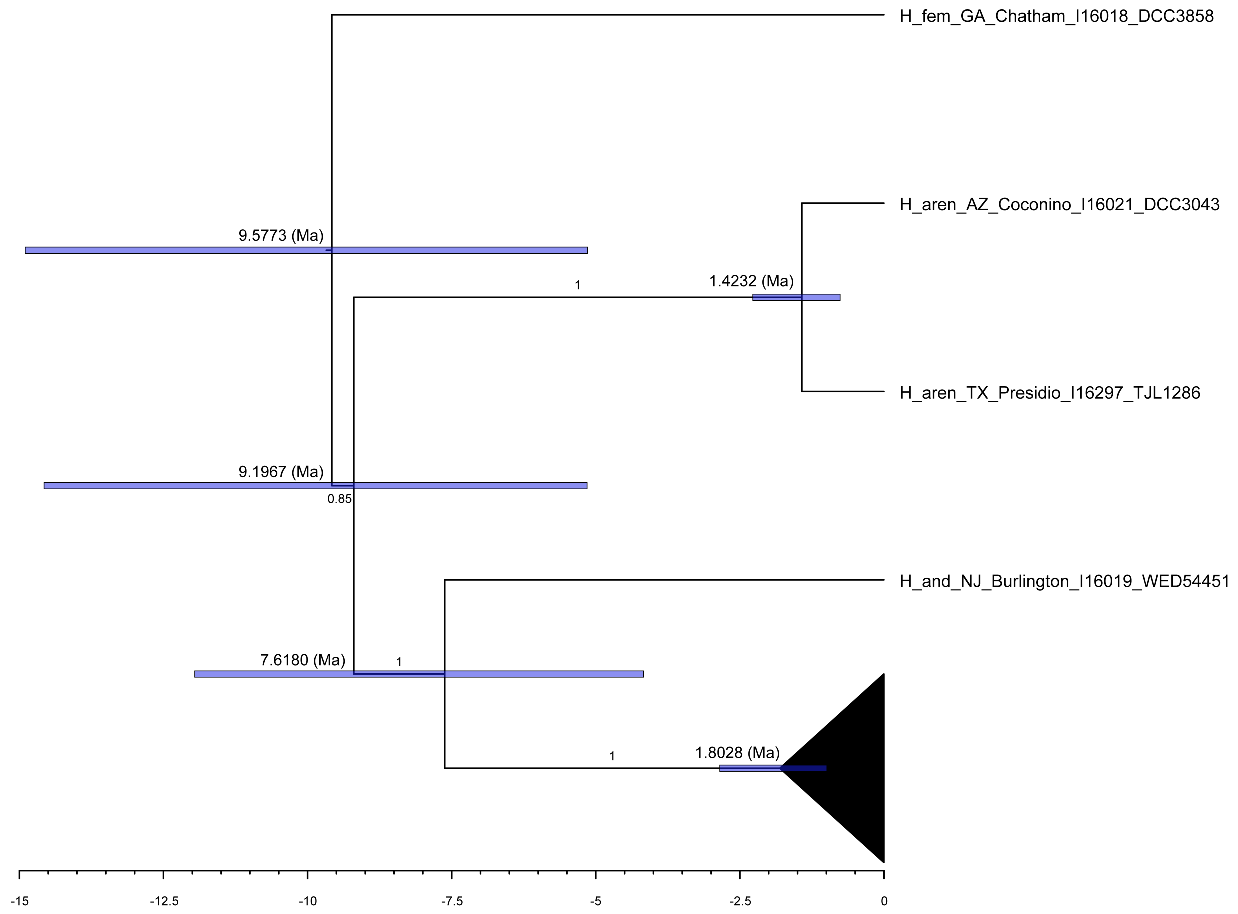

Supplemental Figure 5. Mitochondrial relationships and coalescent timing of outgroup taxa (*H. andersonii*, *H. arenicolor*, and *H. femoralis*) relative to *H. avivoca*, *H. versicolor*, and *H. chrysoscelis* (collapsed) from the Beast 2 analysis. Branch labels show posterior probability. Node bars demonstrate the 95%CI estimated coalescent timing in millions of years (Ma), with the mean estimated coalescent time above the bars.

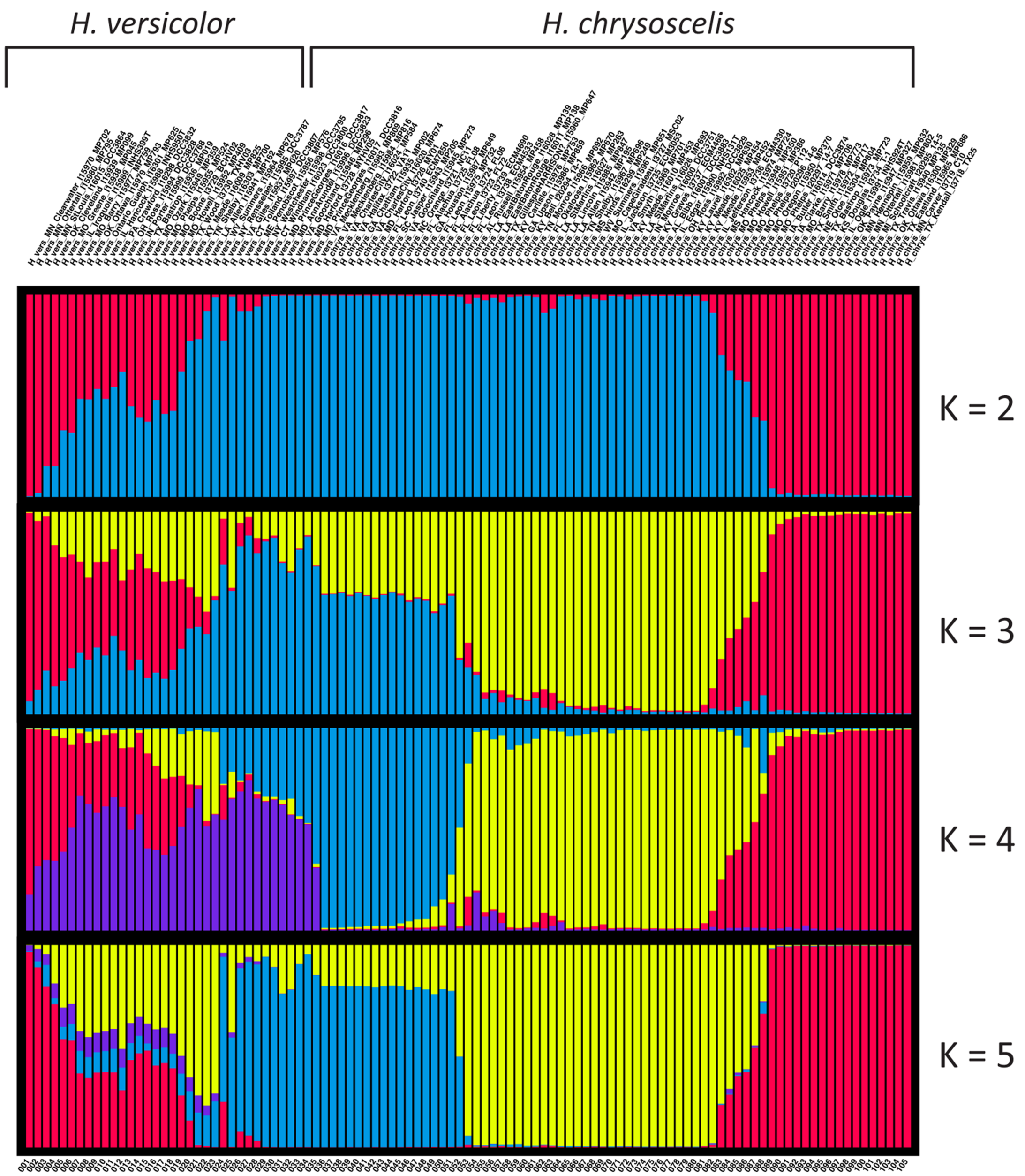

Supplemental Figure 6. STRUCTURE results from an analysis of a single SNP per locus across 244 loci and including all *H. versicolor* and *H. chrysoscelis* samples. Pictured K values stop at 5, because clusters past k=4 were of a small proportion and did not appear to be concentrated across any biologically meaningful group.

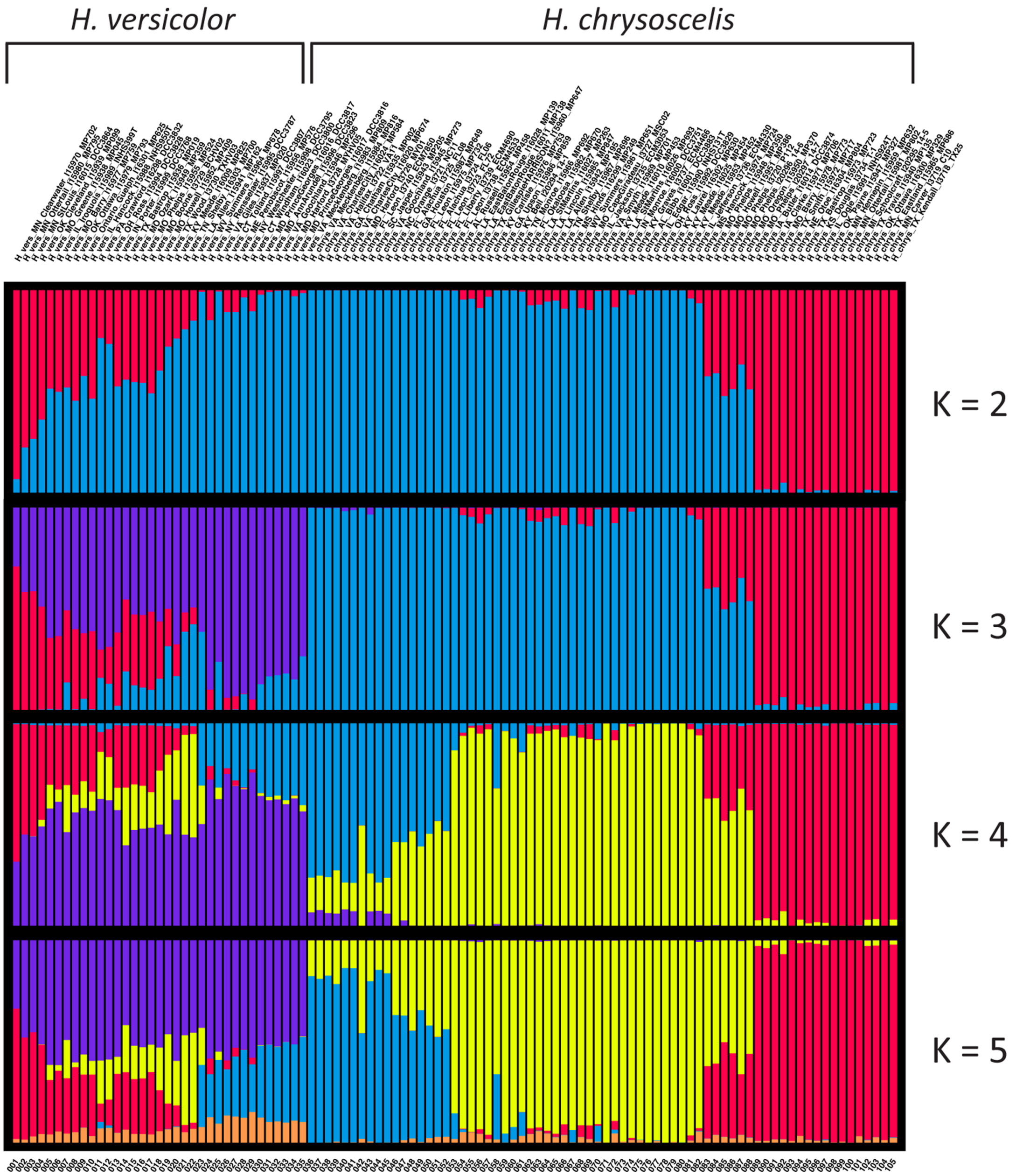

Supplemental Figure 7. STRUCTURE results from an analysis of all 8,683 SNPs across 244 loci and including all *H. versicolor* and *H. chrysoscelis* samples. Pictured K values stop at 5, because clusters past k=4 were of a small proportion and did not appear to be concentrated across any biologically meaningful group.

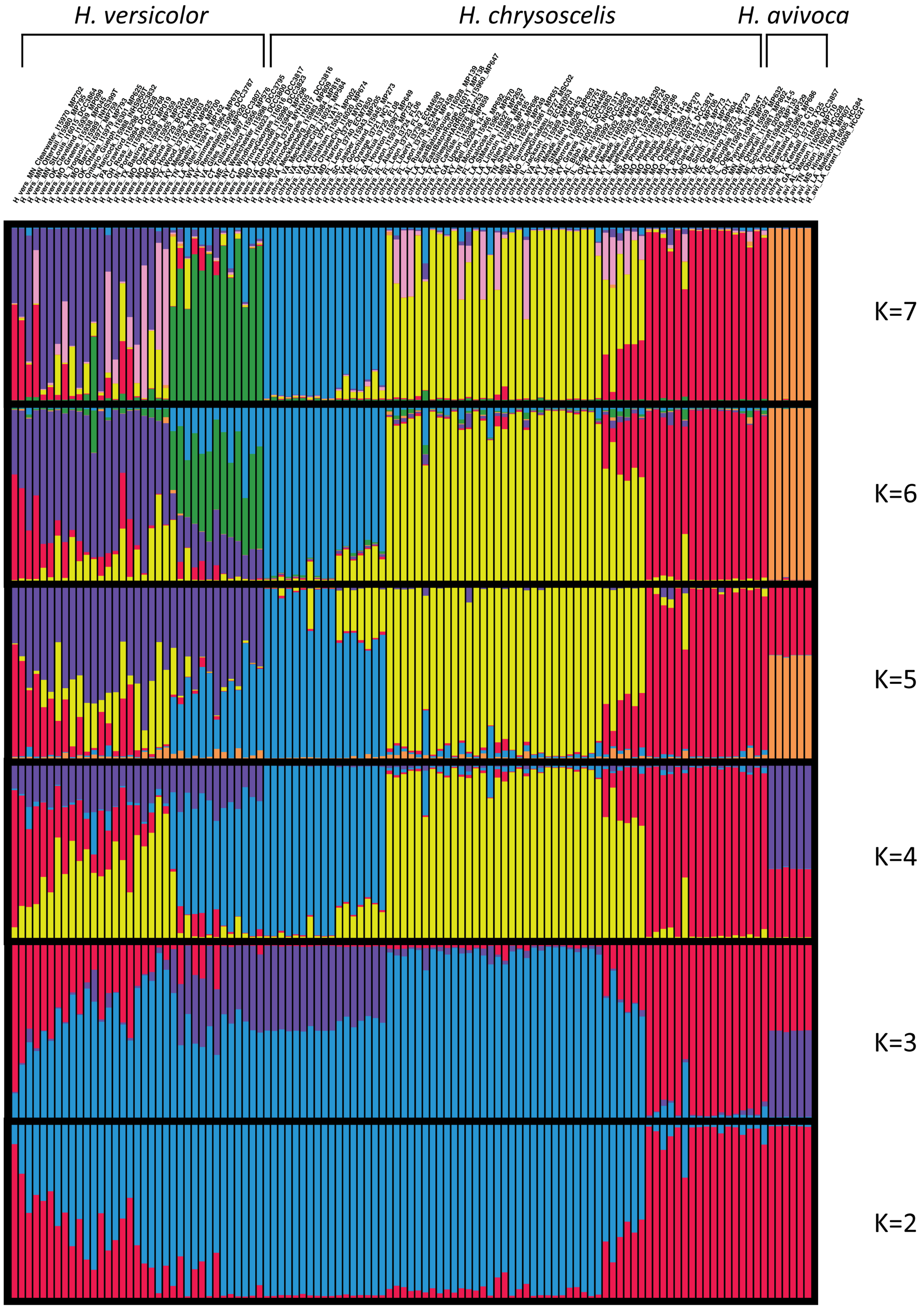

Supplemental Figure 8. STRUCTURE results from an analysis of a single SNP per locus across 244 loci and including all *H. versicolor*, *H. chrysoscelis*, and *H. avivoca* samples. Order is the same as Fig. 6 and 7, with *H. avivoca* on the far right.

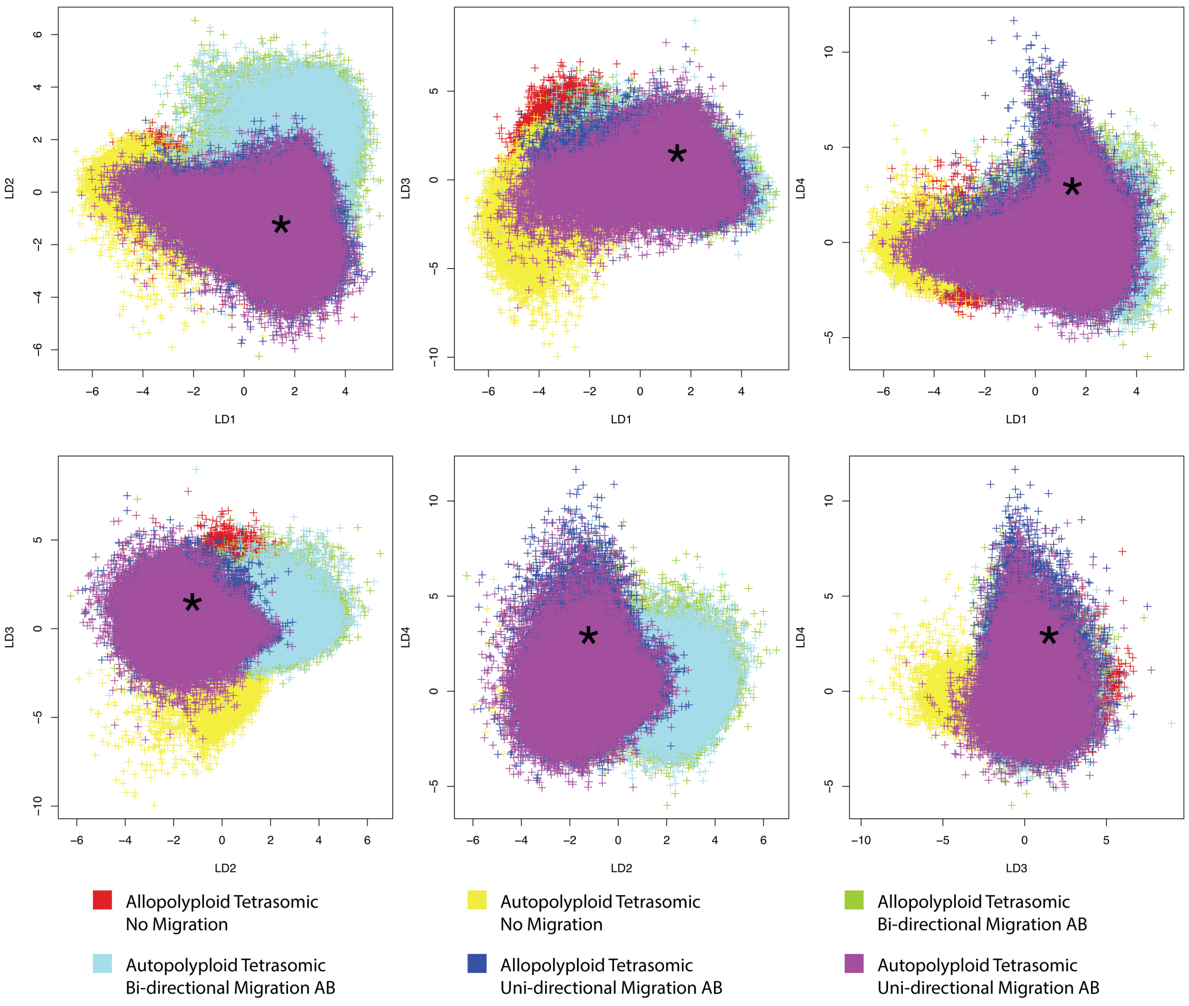

Supplemental Figure 9. Pairwise comparison of LDA axes 1-4 for each of the 6 simulated polyploid speciation and migration models. The star represents the observed LDA values for the Northeast *H. versicolor* and Eastern *H. chrysoscelis* dataset.

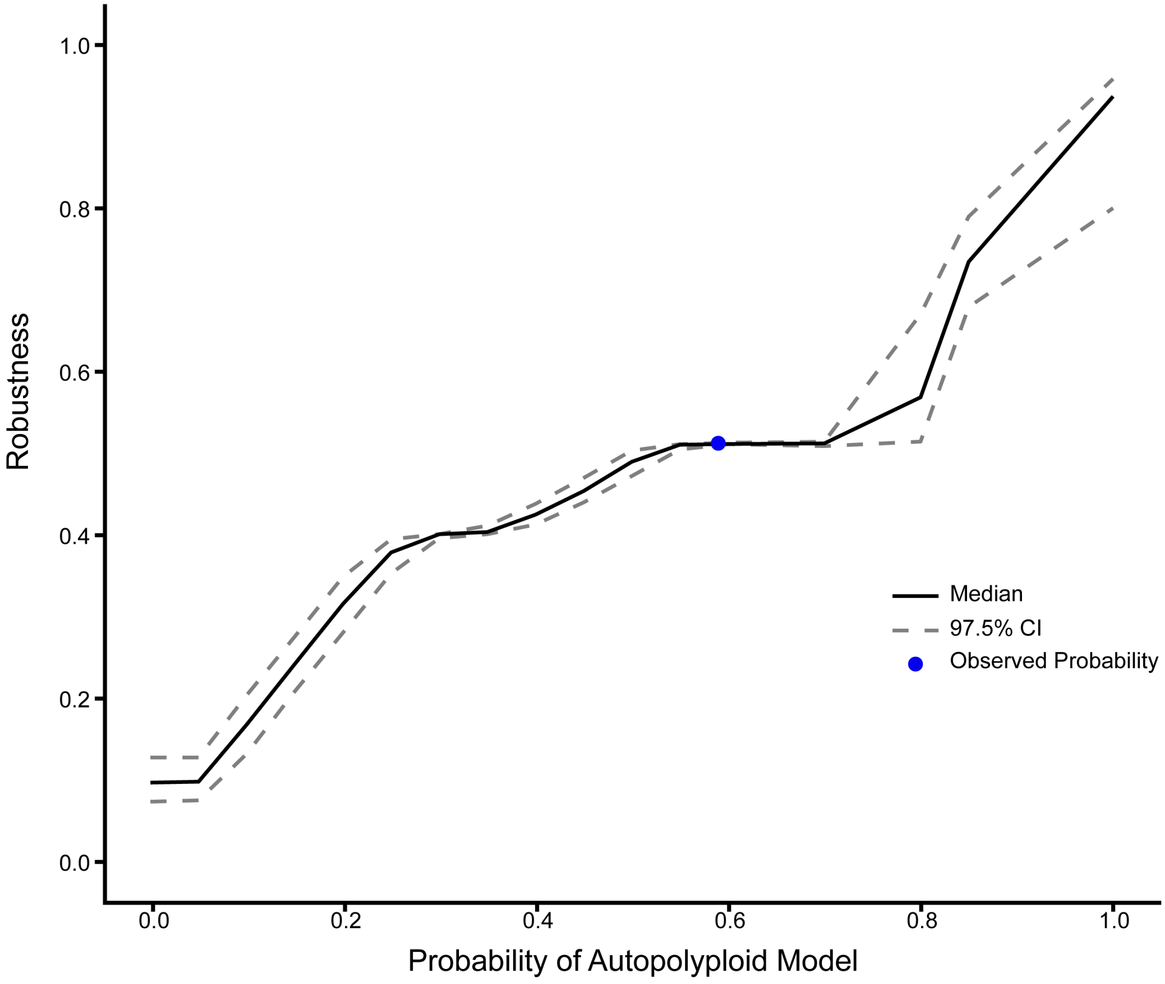

Supplemental Figure 10. Robustness of our ABC analysis to select the true model (here, autopolyploid with tetrasomic inheritance and one-way AB migration) given an estimated probability of that model. Robustness was assessed using 1000 pseudo-observed datasets and calculated as $\frac{P(M_1|M_1)}{P(M_1|M_1)+P(M_1|M_2)}$. The blue circle shows the probability of the Autopolyploid model from our analysis of the observed data.

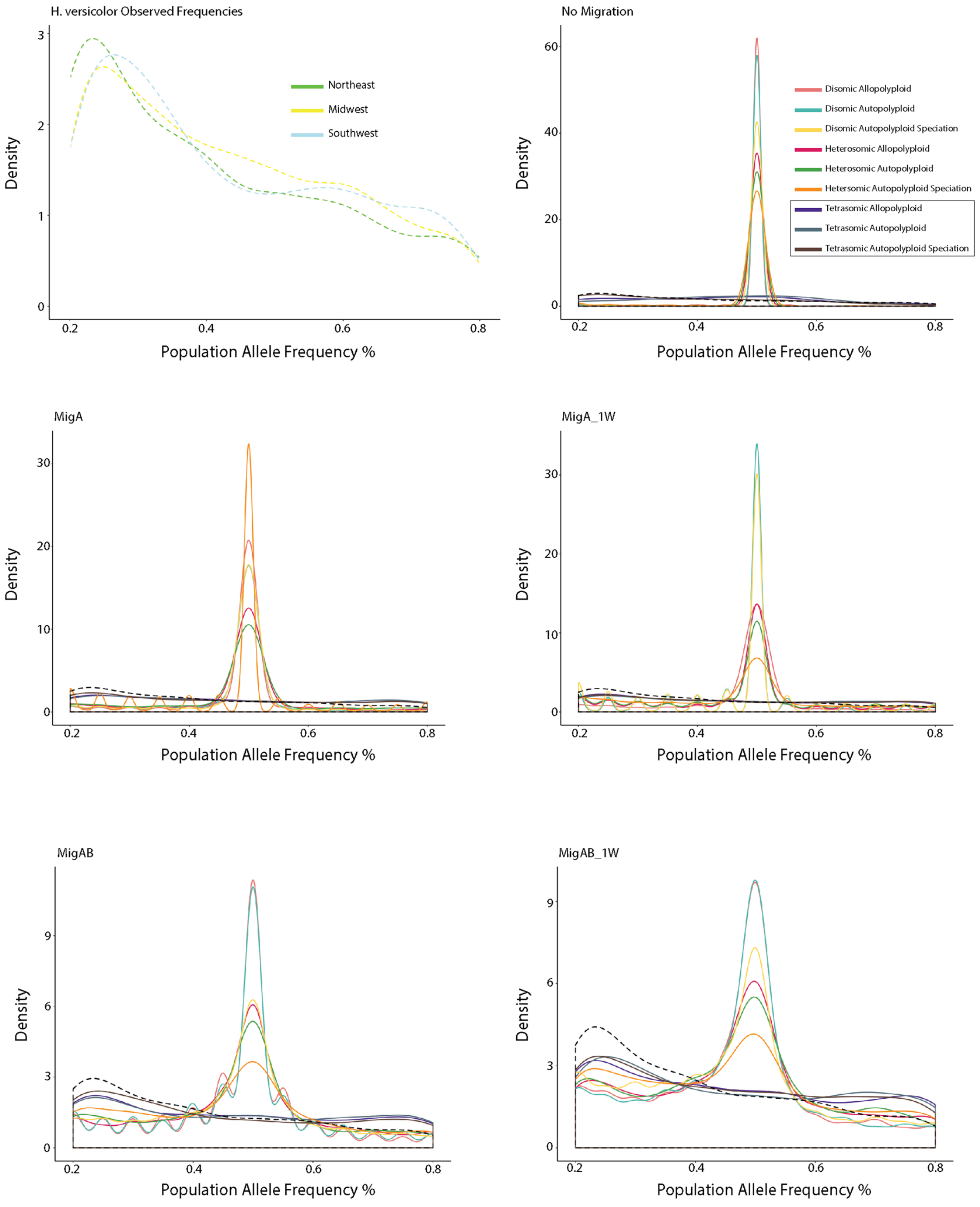

Supplemental Figure 11. Observed and simulated population allele frequencies. First panel: Observed intermediate population allele frequency density plots for each *H. versicolor* mitochondrial lineage. Following panels: Observed and simulated population allele frequency density plots under each polyploid speciation and inheritance model separated by migration model. Simulated allele frequencies are solid lines, observed allele frequency of Northeast *H. versicolor* shown by the dashed line.

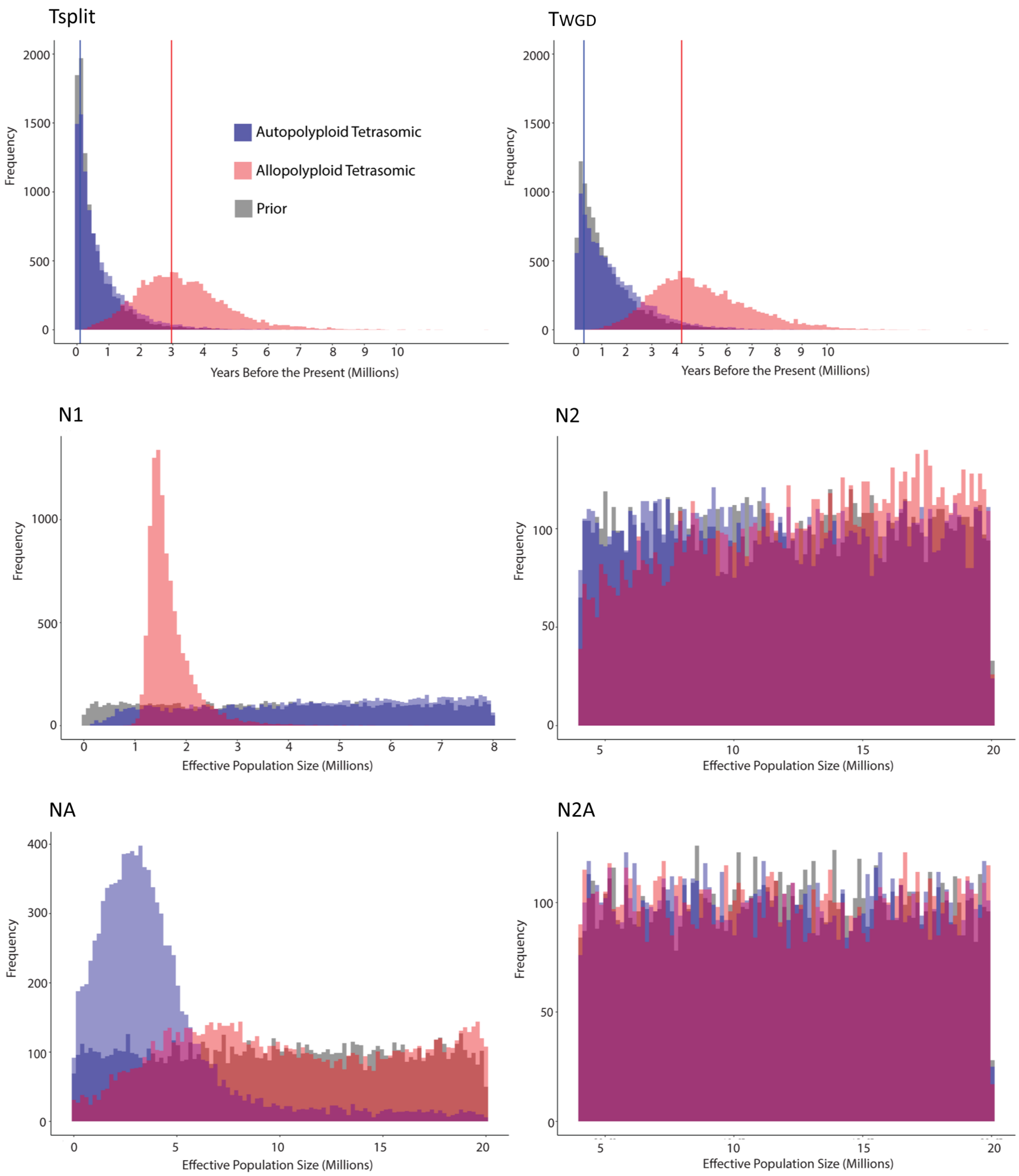

Supplemental Figure 12. Prior and posterior distributions for the two best supported models from the ABC analysis. Both models presented are with a unidirectional migration history. Prior distribution is in gray. Blue and red vertical lines represent the Autopolyploid and Allopolyploid peak distribution values, respectively.

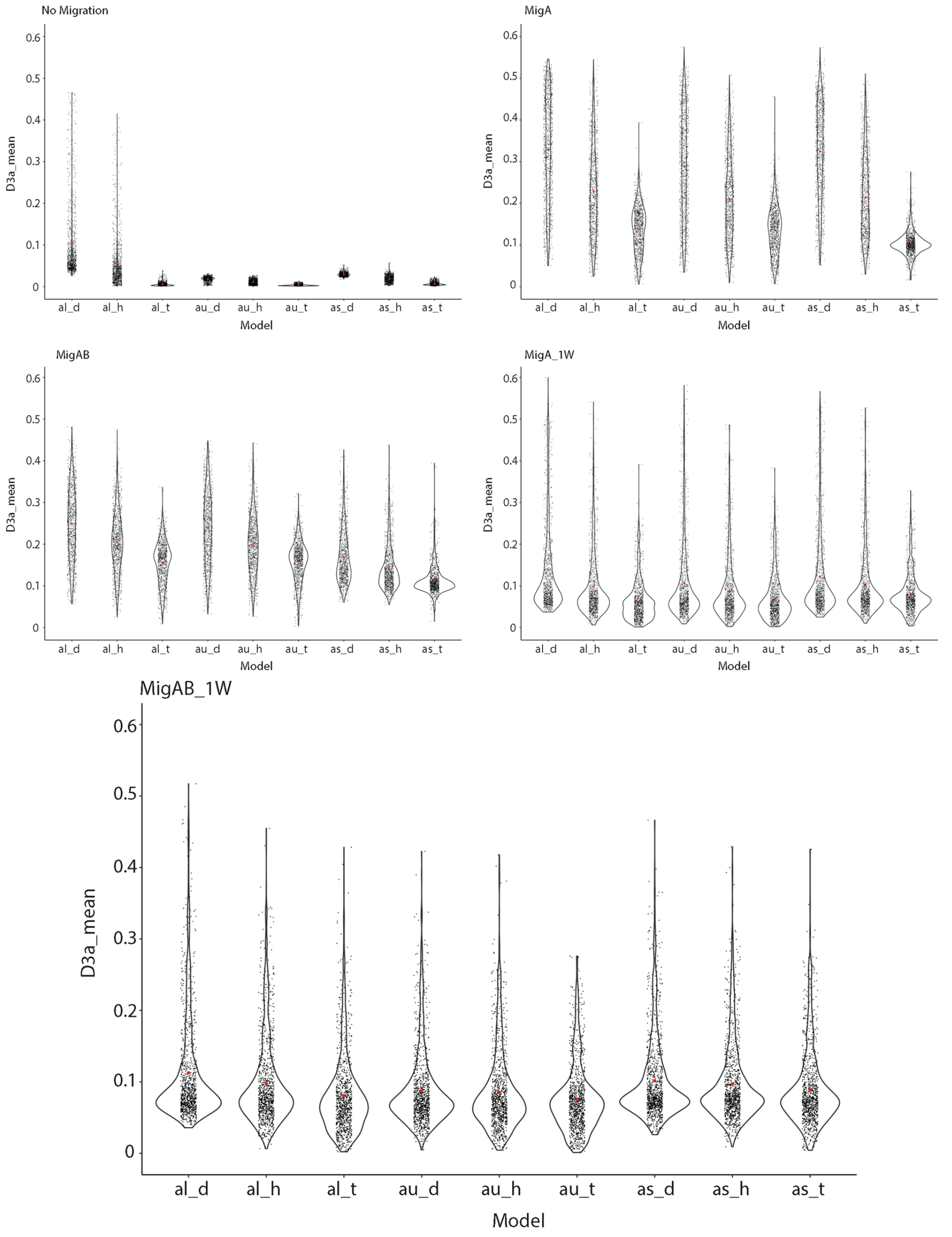

Supplemental Figure 13. Violin plots and observed values for the average of the D3a statistic under each simulated polyploid speciation, inheritance, and migration models. Red dots are the average values for each model.

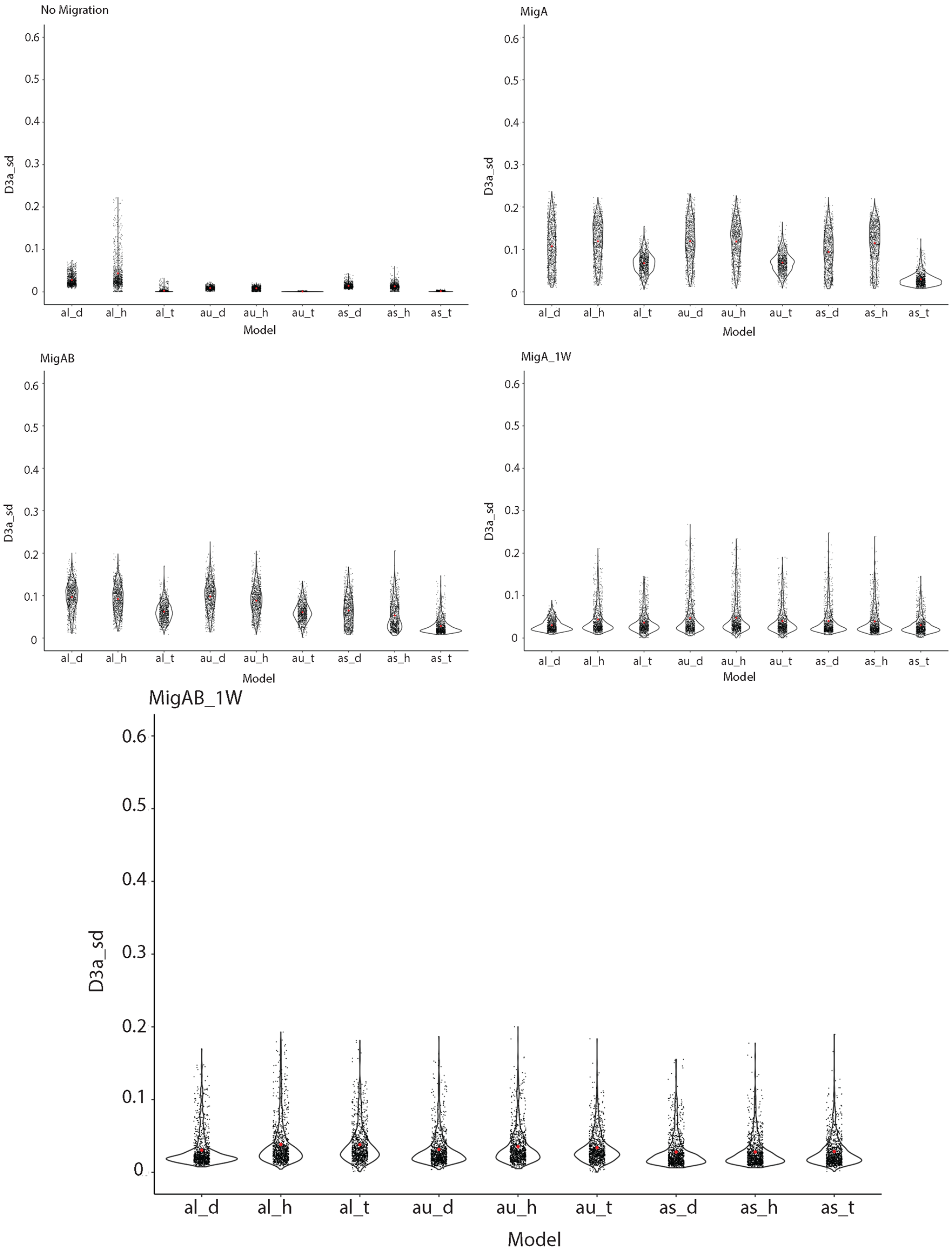

Supplemental Figure 14. Violin plots and observed values for the standard deviation of the D3a statistic under each simulated polyploid speciation, chromosomal inheritance, and migration models. Red dots are the average values for each model.
