# Supplementary figures and images for: The Complex History of Genome Duplication and Hybridization in North American Gray Treefrogs
Source: Mol Biol Evol. 2021 Nov 13;39(2):msab316. doi: 10.1093/molbev/msab316 (PMC8826561; doi:10.1093/molbev/msab316)

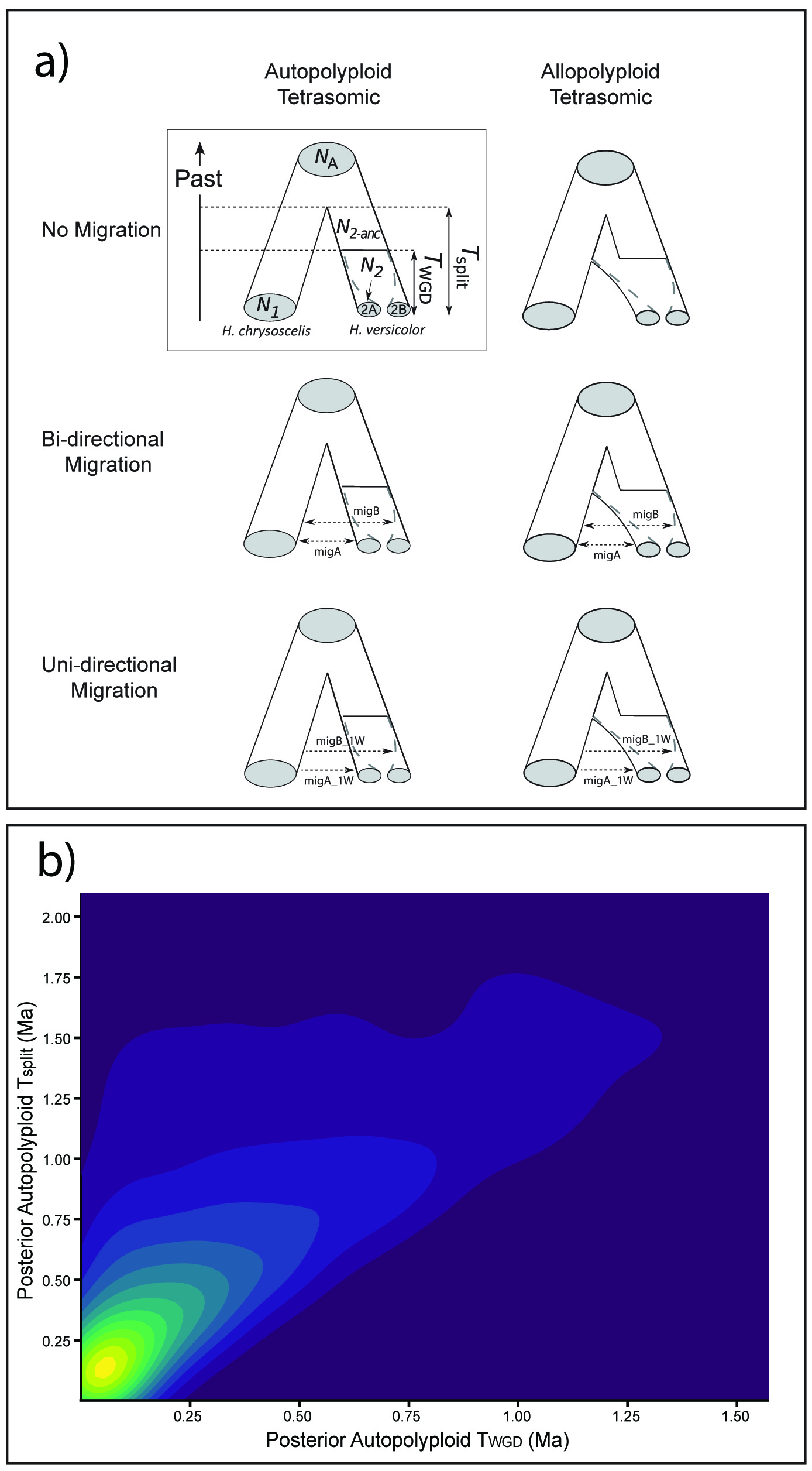

Supplement: msab316_Supplementary_Data [file msab316_supplementary_data.zip › ABC_models_Robust_2dDensity.jpg]

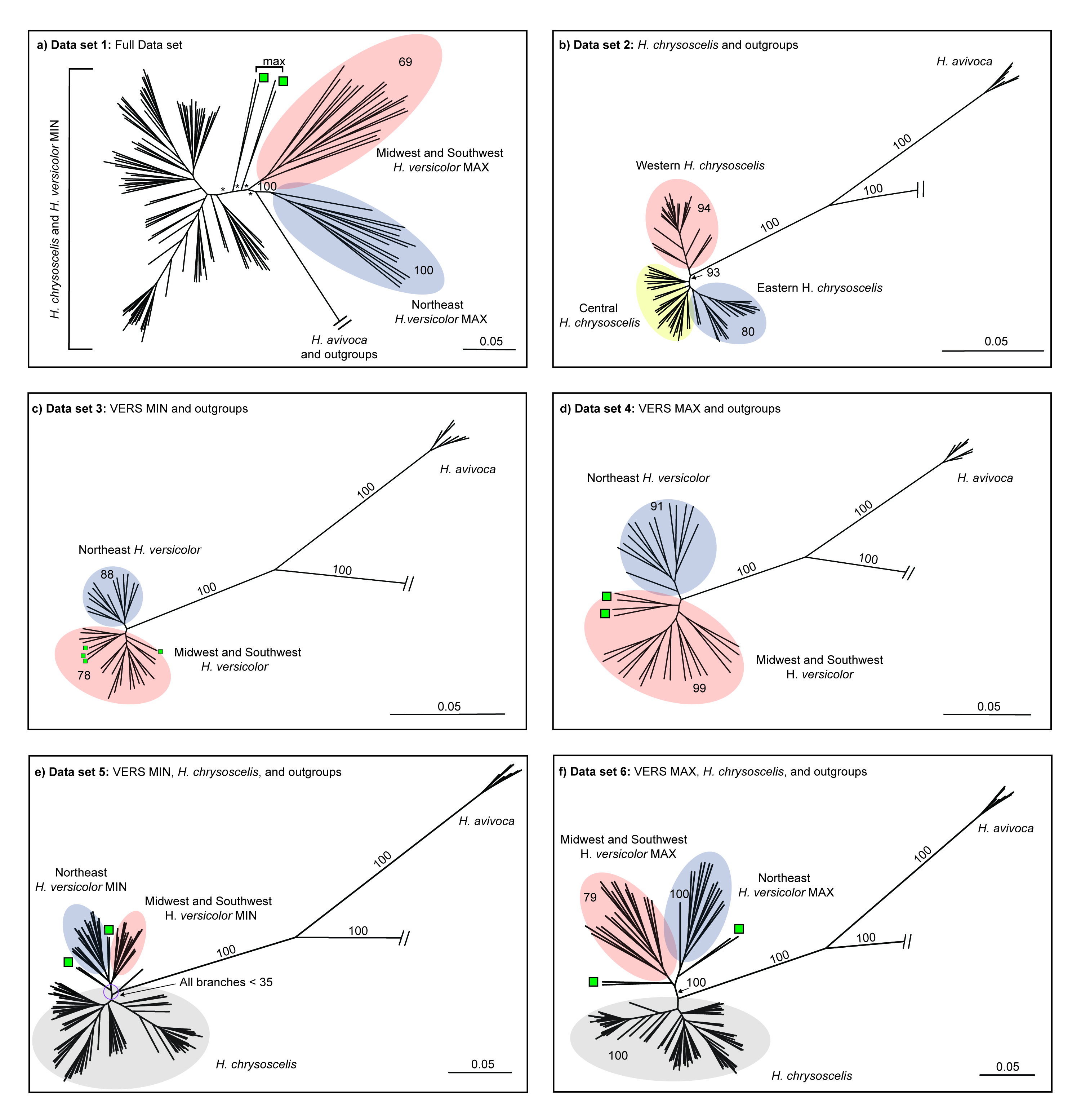

Supplement: msab316_Supplementary_Data [file msab316_supplementary_data.zip › AllTrees.jpg]

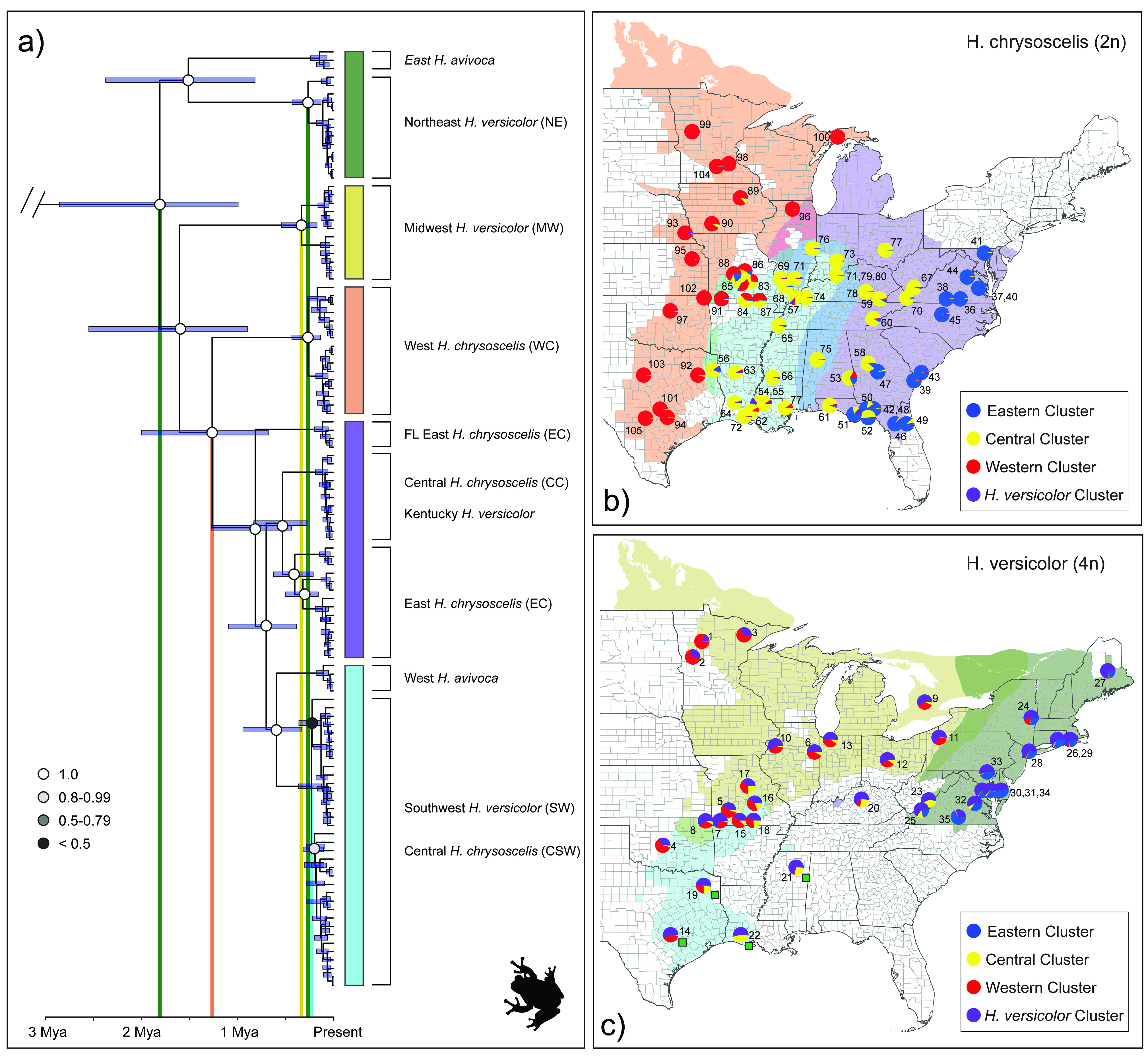

Supplement: msab316_Supplementary_Data [file msab316_supplementary_data.zip › Dated_mtTree.jpg]

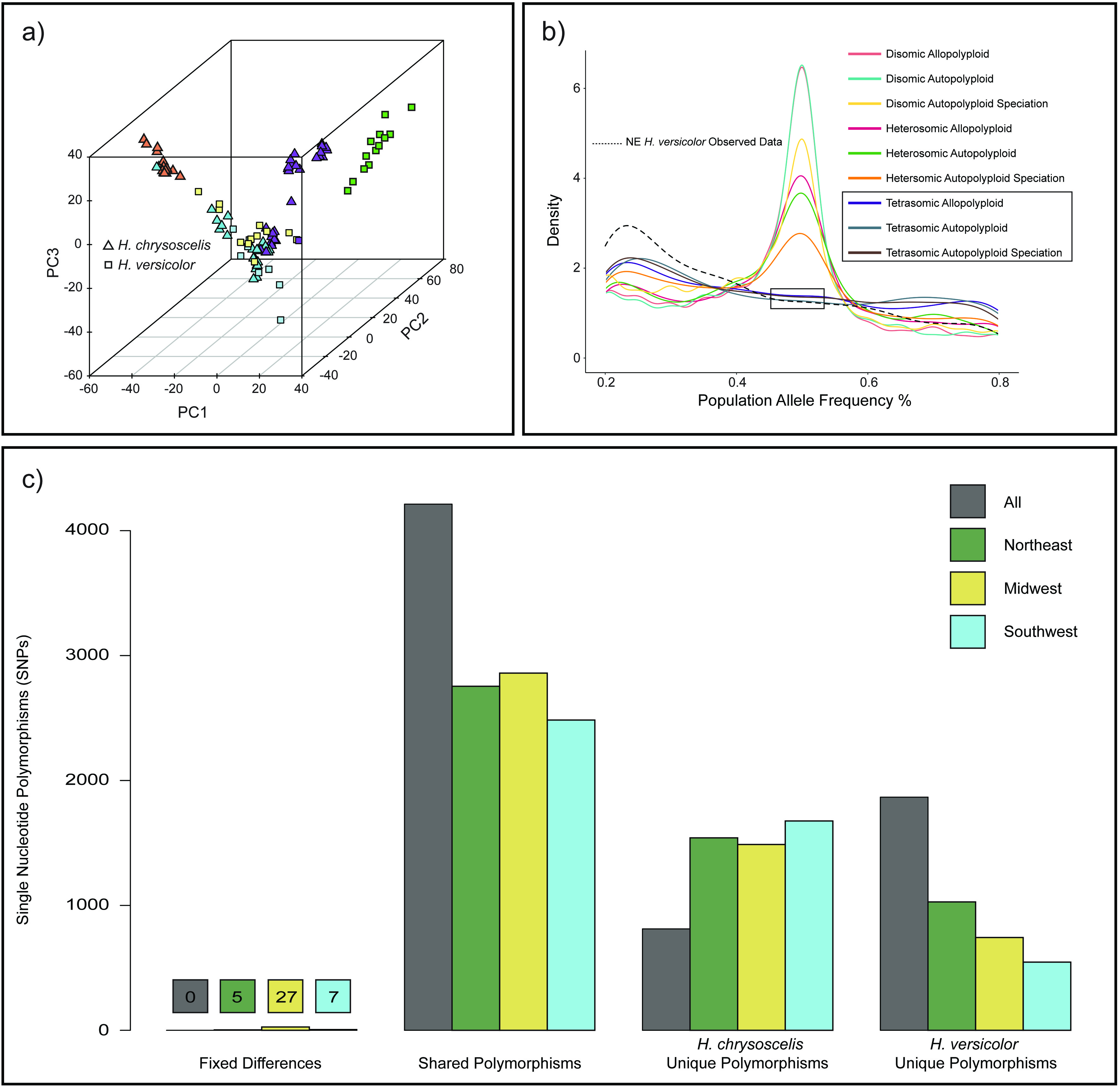

Supplement: msab316_Supplementary_Data [file msab316_supplementary_data.zip › ILS.jpg]

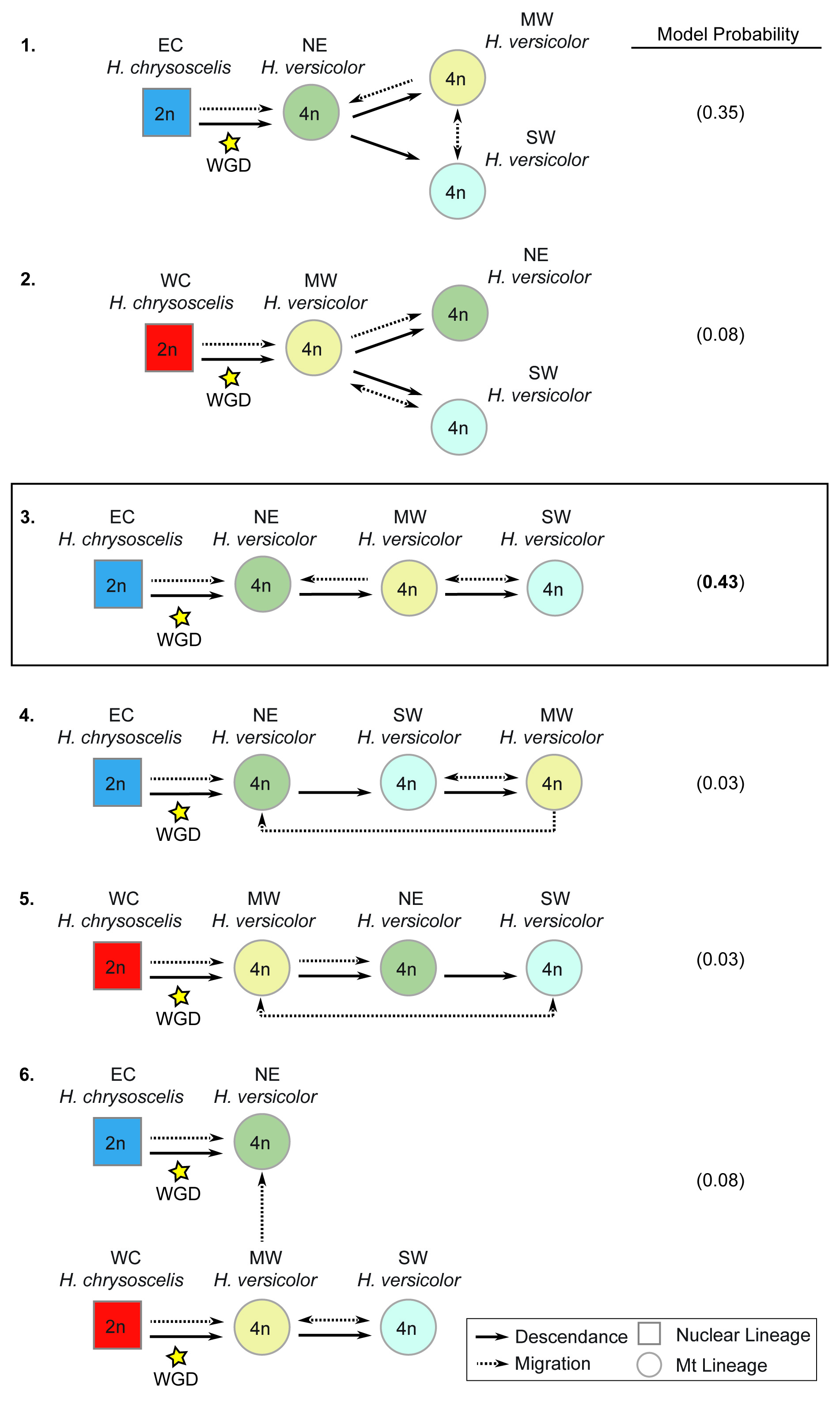

Supplement: msab316_Supplementary_Data [file msab316_supplementary_data.zip › MigrateModels.jpg]

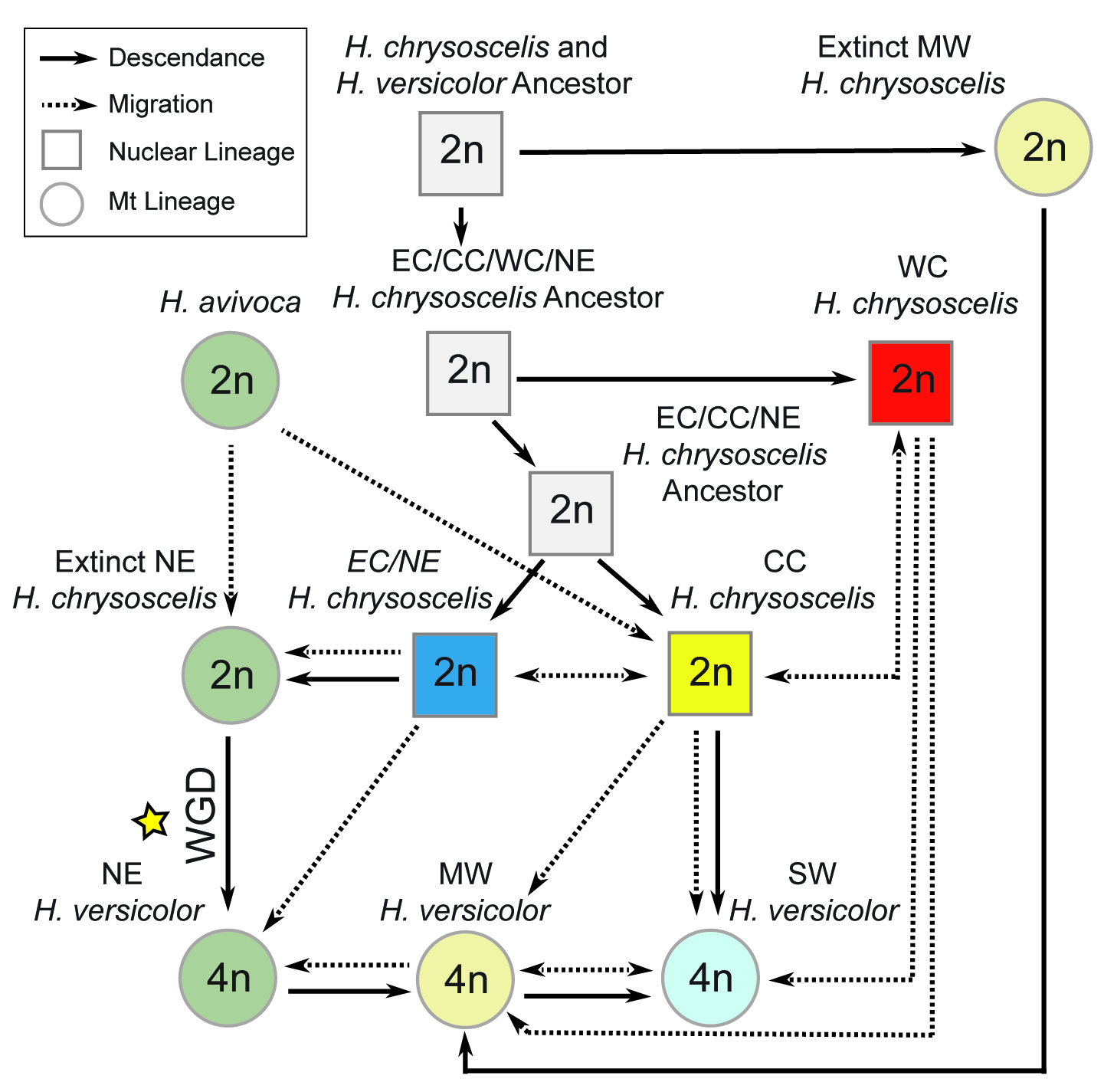

Supplement: msab316_Supplementary_Data [file msab316_supplementary_data.zip › NetworkModelEvo.jpg]
